# Targeting the senescence‒autophagy axis via p16^INK4a^ inhibition alleviates pulmonary fibrosis

**DOI:** 10.1038/s41392-026-02730-4

**Published:** 2026-06-15

**Authors:** Young Jo Yoo, Mi Zhang, Seung-Hyun Jin, Yae Rin Lee, Hee Jin, Thi-Phuong Doan, Sumin Kim, Seri Choi, Eun Jin Park, Sanga Na, Yeonjin Lee, Jiawei Sun, Jaeho Cho, Nam-Chul Cho, So-Yeon Park, Won-Keun Oh, Yun-Sil Lee

**Affiliations:** 1 Graduate School of Pharmaceutical Sciences and College of Pharmacy, Seoul, Republic of Korea; 2 Research Institute of Pharmaceutical Sciences, College of Pharmacy, Seoul National University, Seoul, Republic of Korea; 3 Innovative BioPharmChem Convergence Education and Research Program, Ewha Womans University, Seoul, Republic of Korea; 4 College of Pharmacy and Research Institute of Pharmaceutical Sciences, Sookmyung Women’s University, Seoul, Republic of Korea; 5 Department of Radiation Oncology, Yonsei University Health System, Seoul, Republic of Korea; 6 Drug Information Platform Center, Korea Research Institute of Chemical Technology, Daejeon, Republic of Korea

**Keywords:** Senescence, Target identification, Respiratory tract diseases

## Abstract

Idiopathic pulmonary fibrosis (IPF) is a progressive and fatal lung disease for which effective therapies are lacking. While cellular senescence and autophagy have been implicated in IPF pathogenesis, the intersecting mechanisms that drive fibrosis progression remain unclear. Our patient-derived transcriptomic analysis revealed a strong association between enhanced extracellular matrix remodeling, cellular senescence, and autophagic dysfunction in IPF. Among the potential driver genes associated with cellular senescence, the elevated expression of p16^INK4A^ (p16) is correlated with impaired autophagic flux in naturally aging mice, bleomycin (BLM)- and radiation-induced pulmonary fibrosis (PF) mouse models, and lung tissues from IPF patients. Genetic depletion of p16 reduced PF and preserved autophagic flux in response to fibrotic challenge, independent of p21^Cip1^. Applying this knowledge, we screened antifibrotic compounds from extracts of the medicinal fruit of *Melia azedarach* and identified toosendanin. This compound suppressed p16 promoter activity, effectively restored autophagic flux, and attenuated BLM-induced PF in vivo. We further showed that the p16 promoter inhibitor abyssinone II alleviated both autophagy dysfunction and fibrosis progression, whereas the promoter activator neorautenol exacerbated both phenotypes, demonstrating that p16 acts as a key regulatory node in the regulation of the crosstalk between senescence and autophagy dysfunction. Collectively, our results identify p16 as a mechanistically defined regulator of fibrosis progression and suggest that modulating this axis may offer new opportunities for therapeutic intervention in IPF.

## Introduction

Idiopathic pulmonary fibrosis (IPF) is a progressive and often fatal lung disease characterized by excessive extracellular matrix (ECM) deposition and tissue remodeling. Despite advancements in understanding the molecular mechanisms of fibrosis, effective treatments remain limited, and the exact pathways driving fibrosis progression are poorly understood.^1^ Recent studies have highlighted the role of cellular senescence in age-related diseases, including pulmonary fibrosis.^2^ Senescent cells accumulate in fibrotic tissue and contribute to ECM remodeling through the secretion of proinflammatory factors.^3,4^ The profibrotic role of senescent cells is mediated primarily by the senescence-associated secretory phenotype (SASP), a complex mixture of cytokines, chemokines, growth factors, and proteases secreted by senescent cells. SASP factors, such as interleukin (IL)-6, IL-1α, IL-1β, transforming growth factor (TGF)-β, and matrix metalloproteinases, not only sustain chronic inflammation but also activate neighboring fibroblasts and promote their differentiation into myofibroblasts, key effectors in ECM production and tissue stiffening. Additionally, SASP-driven paracrine senescence can amplify senescence in surrounding cells, creating a self-perpetuating loop that exacerbates tissue damage and the progression of fibrosis.^5^ Indeed, treatment with senolytic drugs, such as a combination of dasatinib and quercetin, has been shown to alleviate PF by selectively eliminating senescent cells.^6,7^ However, further studies are needed to clarify their cell type–specific mechanisms and potential off-target effects.

Autophagy, a cellular process responsible for degrading damaged organelles and misfolded proteins, plays a pivotal role in maintaining cellular homeostasis.^8^ This process involves a tightly regulated sequence of steps: initiation, formation of double-membrane vesicles called autophagosomes, fusion with lysosomes (autolysosomes), and subsequent degradation. Blockage or inefficiency at any stage of the autophagy pathway impairs autophagic flux, which has been implicated in various age-related diseases. In the context of PF, autophagy dysfunction contributes to fibroblast proliferation and migration,^9^ fibroblast-to-myofibroblast differentiation,^10,11^ and alveolar epithelial cell death.^12,13^ Although our understanding continues to evolve, autophagic flux is regulated by several cellular signaling pathways, including mammalian target of rapamycin (mTOR), AMP-activated protein kinase (AMPK), and phosphatidylinositol 3-kinase/protein kinase B (PI3K/AKT). mTOR acts as a central negative regulator of autophagy by inhibiting the unc-51-like autophagy-activating kinase 1 (ULK1) complex, whereas AMPK activates autophagy by promoting ULK1 phosphorylation and suppressing mTOR signaling. On the basis of this rationale, signaling modulators that enhance autophagic flux have been explored as potential treatments for IPF. Several agents have shown promising results in preclinical PF models, including rapamycin/rapalogs (mTOR inhibitors),^14^ metformin (AMPK activators),^15^ and spermidine (natural polyamines that stimulate cytoprotective autophagy).^16^ Nonetheless, these approaches have not yielded clinical benefit and carry the risk of adverse effects, possibly because of the difficulty of selectively modulating autophagic flux without disrupting physiological functions. Notably, recent studies suggest that cellular senescence itself may contribute to impaired autophagy, promoting age-related neurodegenerative processes.^17,18^ However, such a link has not been identified in PF. Therefore, identifying upstream regulators that integrate senescence and autophagy signaling might offer a novel therapeutic strategy to selectively target disease-specific autophagy dysfunction while preserving physiological autophagic homeostasis.

Cyclin-dependent kinase inhibitor 2A (*CDKN2A*, p16^INK4A^; hereafter p16) is a key regulator of cellular senescence that enforces G1 cell cycle arrest by inhibiting CDK4/6-mediated phosphorylation of the retinoblastoma (Rb) protein.^19^ In addition, the emergence of p16-positive fibroblasts after lung injury contributes to fibrotic remodeling^20^ and epithelial cell regeneration,^21^ underscoring the multifaceted role of p16 in PF beyond cell cycle regulation and highlighting the need for further investigation. Building on these observations, we conducted comprehensive transcriptomic analyses and identified elevated p16 expression as a consistent feature across lung tissues from IPF patients and PF model mice. To define its functional role, we investigated how p16 contributes to fibrotic remodeling and autophagy impairment during PF progression using human fibroblast and lung epithelial cells, p16 knockout mice, and bleomycin (BLM) and radiation (IR)-induced PF models. Furthermore, leveraging this mechanistic insight, we established a screening platform for bioactive natural compounds that modulate p16 promoter activity and assessed their therapeutic efficacy in PF.

Collectively, the results of this study introduce several novel aspects to the field: (1) it defines a previously unrecognized role of p16 as a molecular link between senescence and autophagy dysfunction in PF; (2) it provides mechanistic evidence that p16-driven senescence actively suppresses autophagic flux, thereby amplifying fibrotic remodeling; and (3) it establishes a translational platform for identifying natural compounds that restore autophagy by modulating p16 expression. Together, these findings clarify the functional intersection of senescence and autophagy in IPF and suggest that p16-centered regulation is a new therapeutic axis for antifibrotic drug discovery.

## Results

### Increased p16 in IPF lungs is associated with fibrotic remodeling and altered autophagy-related signatures

**Fig. 1:**
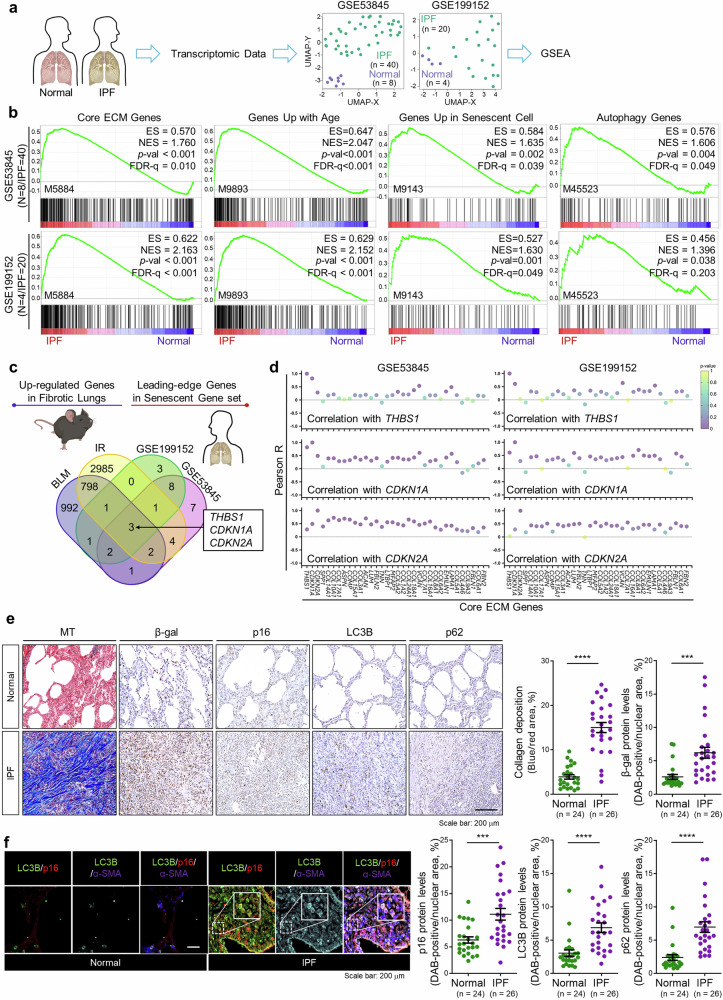
Transcriptomic and histological evidence implicating p16 in IPF. **a** UMAP plots showing clear transcriptional separation between IPF and normal lung tissues in two independent GEO cohorts (GSE53845 and GSE199152), supporting their suitability for pathway-level enrichment analysis. **b** GSEA plots demonstrating enrichment of genes related to ECM, aging, senescence, and autophagy in IPF lungs. **c** Venn diagram depicting shared senescence-associated genes upregulated in IPF patients and two PF mouse models (BLM- or IR-induced). **d** Heatmap showing Spearman’s correlation between p16 (*CDKN2A*), p21 (*CDKN1A*), and *THBS1* and core ECM genes in IPF lung samples. **e** Representative images of MT, β-gal, p16, LC3B and p62 staining and their quantification in IPF and normal lung tissues. *** indicates statistical significance <0.001; **** indicates statistical significance < 0.0001. **f** Colocalization of p16 and LC3B within α-SMA–positive fibrotic regions in IPF lungs. Data are presented as the mean ± SEM; each dot represents an independent biological replicate

To investigate the transcriptomic signatures associated with PF, we first selected two independent IPF cohorts (GSE53845 and GSE199152) that exhibited clear separation between IPF and normal lung samples on the basis of uniform manifold approximation and projection (UMAP; Fig. 1a). We subsequently performed an unbiased comparative gene set enrichment analysis (GSEA) using the C2 curated gene set collection from MSigDB to identify biological processes that were significantly activated or suppressed in IPF relative to normal lung tissues. As a result, we revealed a consistent enrichment of multiple biological processes across both cohorts (Supplementary Table 1), identifying 569 commonly upregulated and 11 commonly downregulated gene sets in IPF lungs compared with normal lungs (Supplementary Fig. 1a). The markedly greater number of upregulated genes than downregulated genes indicates that many biological programs are transcriptionally activated during fibrosis progression. Among the enriched pathways, core ECM genes (gene set ID: M5884) were significantly upregulated in IPF tissues (Fig. 1b, left). In parallel, aging-related gene sets, including genes upregulated with age (M9893) and genes upregulated in senescent cells (M9143), were also significantly enriched in IPF lungs (Fig. 1b, middle). Notably, the expression of autophagy-related genes (M45523) was concomitantly increased in IPF tissues (Fig. 1b, right). Considering that the upregulation of autophagy-related genes does not necessarily indicate increased autophagy but might instead reflect impaired autophagic flux,^22^ we conclude that the transcriptomic signature may suggest dysregulated autophagic processes in fibrotic lungs.

Since the specific senescence-associated markers that actively drive fibrotic remodeling in IPF remain unclear, we focused on identifying senescence-related genes that potentially contribute to fibrogenesis. To this end, we first extracted the leading-edge genes contributing to the enrichment of the cellular senescence gene set (M9143) in two independent IPF patient cohorts (Supplementary Table 2). We then cross-referenced these genes with transcriptomic data from fibrotic lung tissues of multiple mouse models, including BLM- or IR-induced PF models (Supplementary Table 3), from our previous study.^23,24^ We identified three candidate genes, thrombospondin-1 (*THBS1*), cyclin-dependent kinase inhibitor 1A (*CDKN1A*) and 2A (*CDKN2A*), whose expression was consistently upregulated in the fibrotic lungs of both human IPF patients and mouse models (Fig. 1c). THBS1 is a matricellular glycoprotein that modulates cell–matrix interactions and activates latent transforming growth factor (TGF)-β, a key profibrotic cytokine.^25^ The upregulation of THBS1 has been reported in fibrotic tissues and is associated with increased ECM remodeling and tissue stiffness.^26^ *CDKN1A*, also known as p21^Cip1^ (p21), is a cell cycle regulator that induces G1 phase arrest in response to cellular stress.^19^ It is a canonical marker of DNA damage-induced senescence and contributes to fibrosis by promoting the SASP in fibroblasts and epithelial cells.^27^ *CDKN2A*, also referred to as p16, is another critical senescence regulator that inhibits cyclin-dependent kinase 4/6 (CDK4/6), leading to activation of the retinoblastoma (Rb) pathway and irreversible cell cycle arrest. Cells that express p16 accumulate in fibrotic lungs and contribute to disease progression via SASP-mediated profibrotic signaling.^20,21^ In both IPF patient cohorts, we further examined the correlation between the expression of candidate driver genes (*THBS1*, *CDKN1A*, and *CDKN2A*) and that of core ECM genes (Fig. 1c and Supplementary Table 4). While most of the tested ECM genes were positively correlated with all three candidates, *CDKN2A* (p16) exhibited the strongest association (Fig. 1d). To validate this finding, we assessed *CDKN2A* expression levels in additional IPF cohorts from the Gene Expression Omnibus (GEO) database. Similarly, *CDKN2A* was significantly upregulated in fibrotic lung tissues from IPF patients across multiple independent cohorts with larger sample sizes (GSE17978, GSE47460, GSE10667, and GSE24206), whereas the other candidate genes exhibited greater variability in expression patterns (Supplementary Fig. 1b). On the basis of these findings, we focused further on p16 protein levels in human lung tissues. Histological analyses (Fig. 1e) revealed significant increases in p16 protein levels and collagen deposition, as determined by Masson’s trichrome (MT) staining. To assess cellular senescence in formalin-fixed, paraffin-embedded (FFPE) lung tissues, in which a classical senescence-associated β-galactosidase (SA-β-gal) enzymatic assay cannot be performed, we evaluated β-galactosidase (GLB1, β-gal) protein levels by immunohistochemistry (IHC) as a surrogate marker. This approach is based on previous reports demonstrating that GLB1 is the molecular source of SA-β-gal activity and closely parallels senescence-associated changes.^28^ Using this approach, we observed marked increases in β-gal staining in IPF lungs. These changes were accompanied by evidence of impaired autophagic flux, as indicated by the accumulation of microtubule-associated protein 1 light chain 3 beta (LC3B) and p62, both of which are typically degraded via autolysosomal processing under normal autophagic conditions.^29^ Interestingly, both p16 and β-gal protein levels were positively correlated with fibrosis severity, as determined by the extent of collagen deposition (Supplementary Fig. 1c). LC3B and p62 levels were positively correlated in human lung tissues (Supplementary Fig. 1d), and each was significantly positively correlated with fibrosis severity (Supplementary Fig. 1c). Consistent with these findings, the combined autophagy score, calculated as the sum of LC3 and p62 accumulation, was more strongly correlated with fibrosis severity and p16 levels than either marker alone was (Supplementary Fig. 1e). These findings corroborate previous reports suggesting that both LC3B and p62 should be evaluated together to accurately interpret autophagic flux, since both proteins are essential for autophagic flux but should be properly degraded in cells with active autophagic flux; thus, their simultaneous accumulation represents impaired autophagic degradation and abnormal autophagic flux in fibrotic lungs.^29^ Furthermore, we confirmed the concomitant increase in p16 and LC3B levels within the fibrotic area, as indicated by the expression of α-smooth muscle actin (α-SMA)^30^ in lung tissues from IPF patients compared with those from normal controls (Fig. 1f). Together, these findings suggest that p16 is a critical senescence-associated driver of fibrotic remodeling and autophagy-related abnormalities in the pathogenesis of IPF.

### p16 upregulation coincides with fibrosis and autophagy dysregulation across multiple PF mouse models

**Fig. 2:**
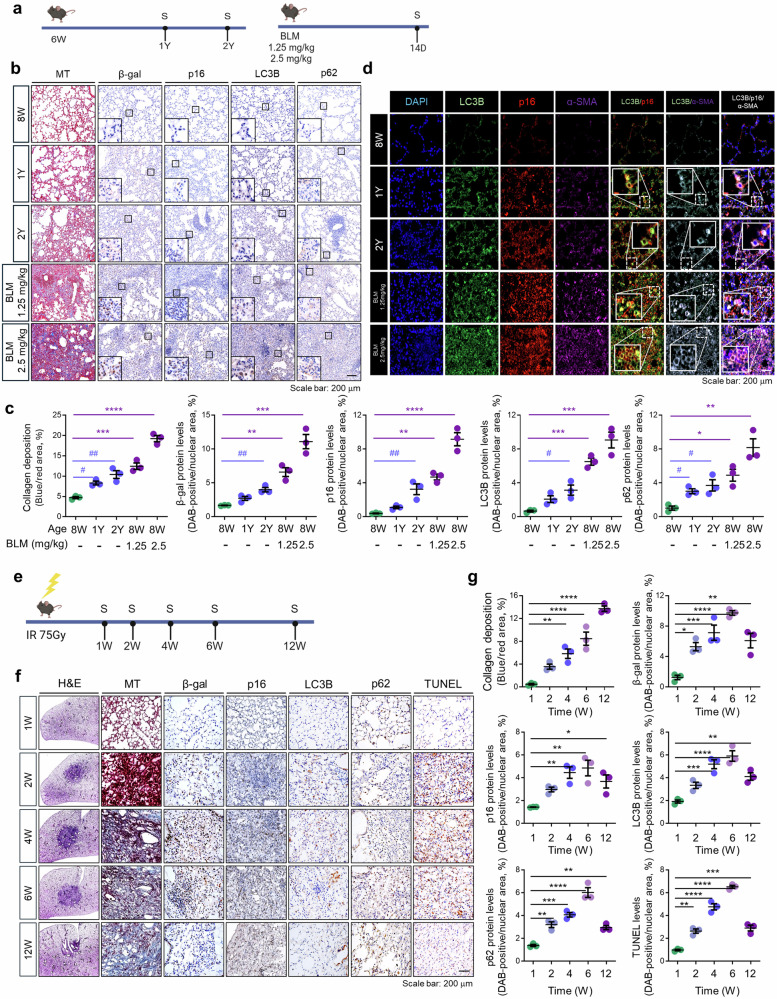
Increased p16 in multiple mouse models of PF along with alterations in autophagy and senescence markers. **a** Schematic of aging- or BLM-induced PF models. **b** Representative images of MT, p16, LC3B, p62 and β-gal staining in the lungs of aged or BLM-treated mice. **c** Quantification of the staining signals shown in (**b**). * denotes statistical significance among the BLM treatment groups (8-week-old naïve control, 1.25 mg/kg, and 2.5 mg/kg BLM), whereas # denotes statistical significance among the age groups (8 weeks, 1 year, and 2 years). **d** Immunofluorescence analysis showing colocalization of p16 and LC3B within α-SMA–positive fibrotic regions. **e** Schematic of the IR-induced PF model and **f** representative images of H&E, MT, β-gal, TUNEL and IHC staining for p16, LC3B, and p62 in IR-treated lungs. **g** Quantification of the positively stained areas shown in (**f**) as a percentage of the total tissue area. Data are presented as the mean ± SEM; each dot represents an independent biological replicate. Statistical significance: *,^#^*p* < 0.05, **^,##^*p* < 0.01^, *^**,^###^*p* < 0.001, ****p < 0.0001

To validate the associations among p16 expression, fibrosis, and autophagy dysregulation in vivo, we generated multiple mouse models of PF, including a BLM-induced model in which mice were treated with two different doses (1.25 and 2.5 mg/kg) and naturally aged at 1 and 2 years of age. The latter represents the near-maximal lifespan of a mouse (Fig. 2a). We assessed the extent of collagen deposition using MT staining and evaluated p16, LC3B, p62, and β-gal protein levels by IHC (Fig. 2b). Collagen accumulation was significantly greater in aged mice (1 and 2 years old) than in young mice (8 weeks old). In parallel, both p16 and β-gal protein levels were significantly elevated in 2-year-old mice, accompanied by a concomitant increase in LC3B and p62 protein levels (Fig. 2c). Compared with untreated control mice, BLM-treated mice exhibited marked collagen accumulation in lung tissues at both doses, along with statistically significant increases in p16, LC3B, p62 and β-gal protein levels (Fig. 2c). Furthermore, immunofluorescence staining revealed a greater degree of colocalization between LC3B and p16 within α-SMA-positive fibrotic regions (Fig. 2d). Collectively, these data suggest that the fibrotic changes induced by aging or BLM treatment are accompanied by increased cellular senescence and elevated LC3B and p62 protein levels.

Given that different fibrotic stimuli may engage distinct cellular pathways, we next used a thoracic IR-induced PF model (Fig. 2e) to evaluate whether the p16-associated changes observed earlier were conserved across fibrosis models. Hematoxylin and eosin (H&E) staining revealed a well-demarcated circular lesion with intensified nuclear staining in the irradiated lung region, indicating localized tissue damage and early fibrotic remodeling. This lesion appeared at 2 weeks post-IR and persisted for up to 12 weeks (Fig. 2f). Since IR is known to induce severe cellular damage,^31^ we assessed the extent of apoptosis using terminal deoxynucleotidyl transferase dUTP nick-end labeling (TUNEL), which can detect DNA strand breaks characteristic of apoptotic cells.^32^ While primarily associated with apoptosis, TUNEL positivity can also occur under conditions of severe cellular stress or DNA damage. We observed that TUNEL-positive areas gradually increased until 6 weeks post-IR and then decreased, after which they returned to levels comparable to those at 2 weeks (Fig. 2g). In contrast, collagen accumulation continued to increase until 12 weeks post-IR (Figs. 2f, g). These results suggest that apoptosis is not strongly correlated with the progressive fibrotic remodeling observed. In addition to progressive morphological changes, apoptosis, and fibrosis, we also observed sustained increases in cellular senescence markers (p16 and β-gal). This was accompanied by evidence of impaired autophagic flux, as indicated by the sustained accumulation of LC3B and p62, both of which are typically degraded via autolysosomal processing under normal autophagic conditions.^29^ Together, these data provide histological evidence that cellular senescence and impaired autophagic flux are concomitantly increased during PF induced by aging, IR, or BLM treatment.

### p16 depletion restores autophagic flux and mitigates fibrotic remodeling

**Fig. 3:**
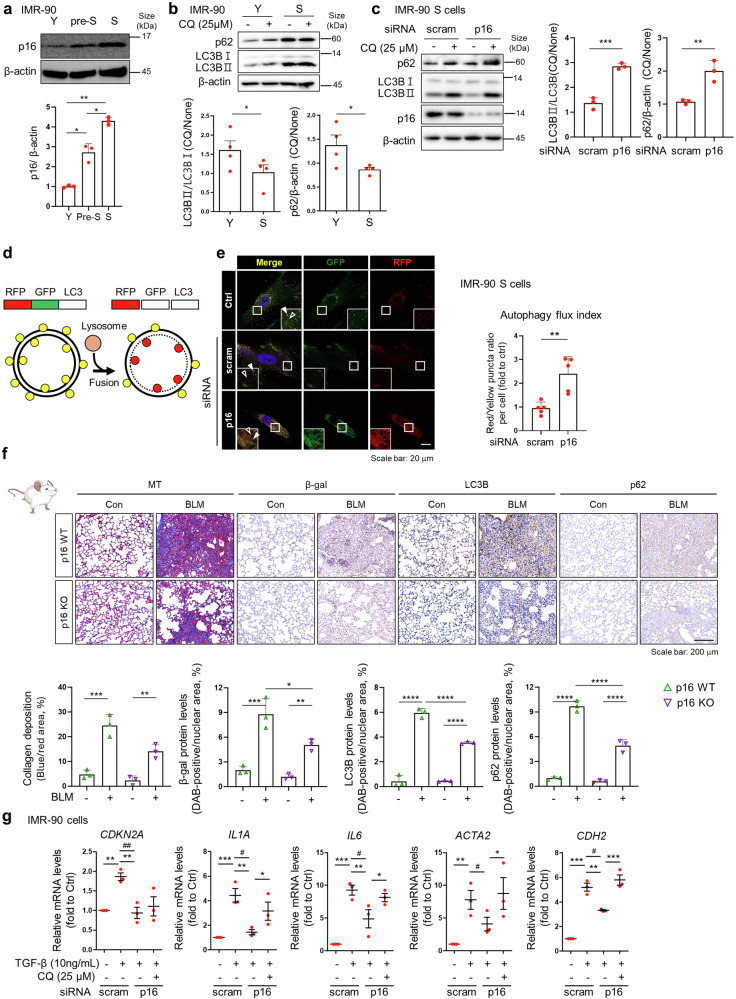
Genetic depletion of p16 restores autophagic flux and attenuates fibrosis. **a** Western blot showing increased p16 levels and **b** reduced CQ-mediated LC3B-II/I conversion and p62 accumulation in senescent (S) versus young (Y) IMR90 fibroblasts. **c** Restoration of LC3B-II/I conversion and p62 accumulation in S fibroblasts following siRNA-mediated knockdown of p16. **d** Schematic of the tfLC3 reporter construct used to distinguish autophagosomes (yellow puncta: GFP + RFP+) from autolysosomes (red puncta: RFP only). **e** Representative images and quantification of tfLC3 fluorescence showing enhanced autophagic flux in S fibroblasts upon p16 knockdown. Autophagic flux was calculated as the ratio of the number of red (RFP⁺ only) puncta to the number of yellow (RFP⁺/GFP⁺) puncta per cell. **f** Histological analysis of BLM-treated WT and p16 KO mouse lungs showing reduced p62, LC3B, and β-gal staining as well as decreased collagen deposition (MT staining). **g** Effect of autophagic flux blockade on p16 knockdown–mediated suppression of fibrotic and senescence-associated transcriptional programs. CQ was used to inhibit autophagic flux during TGF-β–induced fibrogenesis and senescence to assess whether blocking autophagy reversed the antifibrotic and anti-senescent effects of p16 knockdown. * denotes statistical significance among all experimental groups, whereas # denotes significance between the control and p16 siRNA-treated groups. Statistical significance: *, ^#^*p* < 0.05, **^, ##^*p* < 0.01^, *^**p < 0.001

To investigate whether p16 plays a pivotal role in cellular senescence and autophagic flux during fibrotic remodeling, we used a replicative senescence model,^33^ in which IMR-90 human lung fibroblasts were serially passaged until they reached a population doubling (PD) level greater than 34. At this point, the cells exhibit classical features of senescence, including growth arrest, enlarged morphology, and increased senescence-associated β-galactosidase (SA-β-gal) activity, as measured under suboptimal pH conditions (pH 6.0), reflecting the increased lysosomal content in senescent cells.^34,35^ Presenescent (Pre-S) cells were defined as those between PD 15 and 30 that retained their proliferative capacity and showed minimal senescence-associated changes. Compared with that in proliferating young (Y) cells (PD 9), SA-β-gal activity markedly increased in Pre-S IMR-90 cells (PD 17) (Supplementary Fig. 2a). The protein level of p16 was elevated in Pre-S fibroblasts and further increased in fully senescent (S) fibroblasts (greater than PD 34) compared with Y fibroblasts (Fig. 3a). To compare autophagic flux between Y and S cells, we treated both cells with or without chloroquine (CQ), a lysosomotropic agent that blocks autophagosome–lysosome fusion, to assess autophagosome formation rates by measuring the conversion of LC3B-I to LC3B-II.^36^ During autophagy, the cytosolic form of LC3B-I is lipidated to form LC3B-II, which is recruited to autophagosomal membranes; thus, the extent of LC3B-I to LC3B-II conversion upon CQ treatment serves as a dynamic indicator of autophagic flux. Y fibroblasts exhibited a robust increase in the ratio, indicating active autophagic flux, whereas S fibroblasts showed only a modest change, suggesting impaired LC3 processing and flux limitation (Fig. 3b). Consistent with this pattern, CQ treatment induced a greater accumulation of p62 in Y fibroblasts than in S fibroblasts, whereas S cells displayed only a modest increase in p62 expression, further supporting impairment of autophagic degradation. Enhanced accumulation of both LC3-II and p62 following lysosomal inhibition has been previously reported as a characteristic indicator of increased basal autophagic flux.^37^ In this context, we silenced p16 expression using small interfering RNA (siRNA; Supplementary Fig. 2b) and assessed its effect on autophagic flux in S IMR-90 cells by performing a CQ-mediated LC3B-I/II and p62 turnover analysis (Fig. 3c). The CQ-induced conversion of LC3B-I to LC3B-II was significantly enhanced upon p16 knockdown. Consistent with these findings, p16 knockdown also led to greater accumulation of p62 upon CQ treatment in S fibroblasts, providing integrated LC3–p62 evidence for p16 knockdown–mediated restoration of autophagic degradation. To further validate this observation, we used tandem fluorescent protein-tagged LC3 (tfLC3), in which LC3 is tagged with red fluorescence protein (RFP) and green fluorescence protein (GFP) at its N-terminus.^38^ In the acidic environment of autolysosomes, GFP fluorescence is quenched, while RFP remains stable, allowing the distinction between autophagosomes (yellow puncta) and autolysosomes (red puncta; Fig. 3d). The tfLC3 probe confirmed that p16 knockdown increased the numbers of both yellow and red puncta (Fig. 3e and Supplementary Fig. 2c) and significantly increased the flux index (red/yellow puncta ratio). Importantly, quantification of the total cell red/green fluorescence intensity ratio revealed no significant changes (Supplementary Fig. 2d), confirming that the observed puncta dynamics reflect true autophagic flux rather than alterations in cellular acidity. Taken together, these results indicate that p16 knockdown not only promotes autophagosome and autolysosome formation but also restores autophagic flux. However, knockdown of p21 in S IMR-90 cells did not affect the extent of LC3B-I-to-LC3B-II conversion following CQ treatment (Supplementary Fig. 2e, f). These findings suggest that, unlike p16, p21 plays a minimal role in autophagic flux in replicatively senescent cells.

To determine whether the in vitro findings regarding the role of p16 in regulating senescence and autophagic flux are conserved in vivo, we used a BLM-induced PF model with p16 knockout (KO)^39^ and wild-type (WT) FVB/N (FVB) mice. We treated both groups with BLM and conducted histological analyses. These data confirmed that p16 KO alleviated BLM-induced collagen deposition and reduced β-gal protein levels. In parallel, the aberrant accumulation of LC3B and p62 proteins in response to BLM treatment was dramatically reduced in p16 KO mice (Fig. 3f). To further address the mechanistic link between p16 inhibition and autophagy in the regulation of fibrosis, we performed complementary cellular experiments. As shown in Fig. 3g, in IMR-90 cells under TGF-β-induced senescent and fibrotic conditions, which exhibit increased p16 (CDKN2A) expression, knockdown of p16 significantly attenuated fibrotic and senescence-associated transcriptional responses, including increased expression of *IL1A* (IL-1α), *IL6* (IL-6), *ACTA2* (α-SMA), and *CDH2* (N-cadherin). Notably, pharmacological blockade of autophagic flux with CQ restored the expression of these fibrotic and senescence-associated genes, reversing the suppressive effects of p16 knockdown, despite a sustained reduction in p16 expression. Next, given the increasing evidence that lung epithelial cells can undergo EMT-like fibrogenic conversion and contribute to myofibroblast accumulation during pulmonary fibrosis,^1^ we additionally examined p16-dependent autophagy regulation in the lung epithelial cell line L132 as a complementary model. Consistently, p16 knockdown significantly suppressed TGF-β-induced fibrotic responses in lung epithelial cells, whereas CQ treatment restored the expression of fibrotic markers (Supplementary Fig. 2g). These findings indicate that the antifibrotic activity of p16 inhibition is mediated through the restoration of autophagic flux, which places autophagy downstream of p16 in the regulation of fibrotic responses. In parallel, p16 knockdown reduced SA-β-gal activity in S IMR-90 cells, which increased upon autophagy inhibition by CQ (Supplementary Fig. 2h). Collectively, these data indicate that p16 functions as an upstream regulator of autophagic flux, thereby driving cellular senescence and fibrogenic progression in PF.

### Identification of toosendanin as a dual modulator of p16-mediated senescence and autophagy

**Fig. 4:**
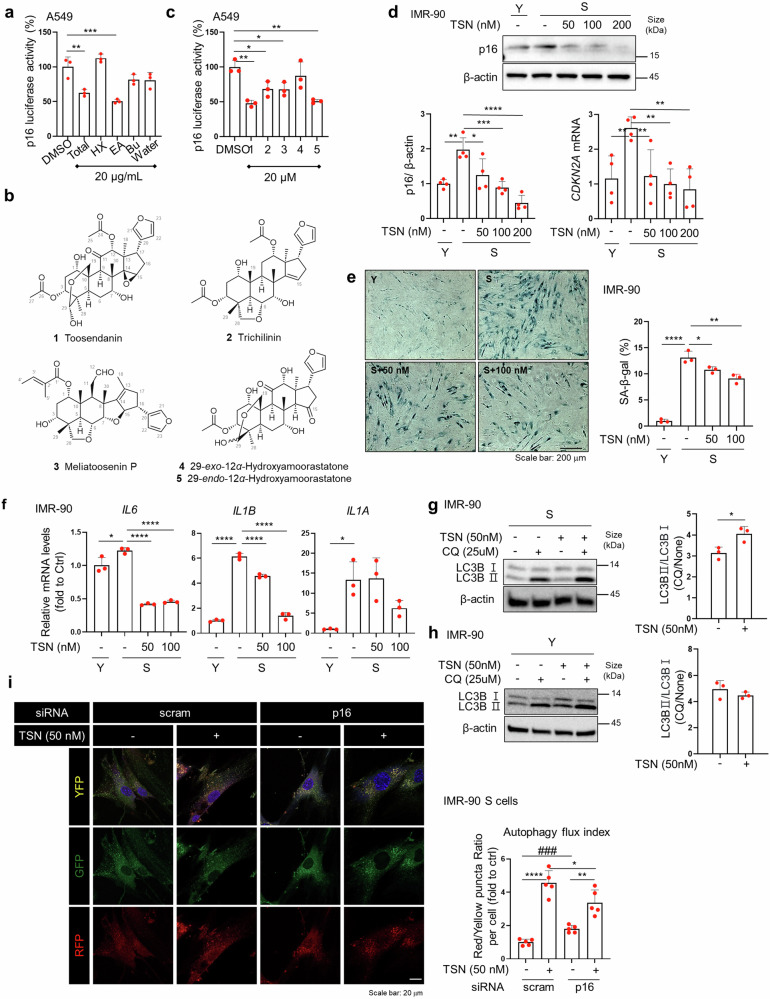
Identification of p16-targeting TSN, which reduces cellular senescence and restores autophagic flux. **a** p16 promoter-driven luciferase screening of solvent-partitioned fractions from *Melia azedarach* fruit extract revealed that the EtOAc fraction was the most potent at suppressing p16 promoter activity. **b** Structures of major compounds purified from the EtOAc fraction. **c** p16 promoter assay for single compounds #**1** ~ **5**. **d** TSN treatment reduces p16 expression at the mRNA and protein levels in senescent (S) fibroblasts in a dose-dependent manner. **e** TSN decreases SA-β-gal activity in S cells, indicating reduced senescence. **f** mRNA expression of SASP genes such as IL-6 (*IL6*), IL-1β (*IL1B*), and IL-1α (*ILA*) in S IMR-90 fibroblasts compared with Y IMR-90 fibroblasts following TSN treatment. protein levels of CQ-mediated LC3B-II/I conversion in S IMR-90 fibroblasts compared with young (Y) IMR-90 fibroblasts after TSN treatment. **g** TSN increases the CQ-mediated LC3B-II/I ratio in S fibroblasts but not in Y fibroblasts (**h**), suggesting the selective enhancement of autophagic flux in senescent cells. **i** Representative tfLC3 fluorescence images and quantification showing enhanced autophagic flux in senescent fibroblasts upon TSN treatment and reduced TSN-induced flux in p16 siRNA-treated cells. Autophagic flux was calculated as the ratio of the number of red (RFP⁺ only) puncta to the number of yellow (RFP⁺/GFP⁺) puncta per cell. * denotes statistical significance among all experimental groups, whereas # denotes significance between the control and p16 siRNA-treated groups. The data are presented as the mean ± SEM; each dot represents an independent biological replicate. Statistical significance: *p < 0.05, **p < 0.01, ***^,###^*p* < 0.001, ****p < 0.0001

Having established the role of p16 in the association between cellular senescence and impaired autophagic flux during PF, we next aimed to identify bioactive compounds capable of modulating these two processes simultaneously. Natural products represent a rich and diverse source of bioactive molecules with therapeutic potential, especially for complex diseases such as fibrosis that involve multiple interrelated cellular pathways. In particular, plants used in traditional medicine often harbor structurally unique secondary metabolites with potent bioactivities. In this context, we focused on *Melia azedarach*, a medicinal plant traditionally used in East Asia for the treatment of inflammatory and respiratory conditions.^40–42^ Based on these attributes, we hypothesized that its constituents include compounds capable of modulating PF. To identify such compounds, we first performed solvent-based fractionation of *Melia azedarach* fruit extract. Dried fruits were extracted with 70% ethanol, and the crude extract was sequentially partitioned into n-hexane, ethyl acetate (EtOAc), n-butanol (n-BuOH), and aqueous fractions (Supplementary Fig. 3a). Next, we used a luciferase reporter assay system driven by the human p16 promoter (Supplementary Fig. S3b) to evaluate the inhibitory effect of each solvent fraction on transcriptional activity. Among these fractions, the EtOAc fraction demonstrated the most potent suppression of p16 promoter activity without cytotoxicity (Fig. 4a, Supplementary Fig. 3c). This fraction was subsequently subjected to stepwise chromatographic purification, leading to the isolation of five compounds (Fig. 4b, Supplementary Tables 5, 6, and Fig. 3a). Among these compounds, compound **1** (toosendanin, TSN) exhibited the strongest inhibitory effect on p16 transcriptional activation as a single compound, with no observable cytotoxicity at effective concentrations (Fig. 4c and Supplementary Fig. 3b–d). To investigate the effect of TSN on cellular senescence, we induced replicative senescence in IMR-90 human lung fibroblasts by continuous passaging until PD 40 and subsequently treated the cells with TSN for 72 h. Treatment with 50 to 200 nM TSN led to a dose-dependent decrease in p16 expression at both the mRNA and protein levels in S cells (Fig. 4d). Notably, 100 nM TSN reduced p16 expression to levels comparable to those in Y cells, whereas 50 nM TSN led to partial suppression. In parallel, TSN significantly reduced the senescent phenotype and SA-β-gal activity in S cells (Fig. 4e). In parallel, among the SASP cytokines, we confirmed that compared with Y cells, S cells expressed significantly higher levels of *IL6*, *IL1A* and *IL1B* and that TSN treatment significantly reduced the levels of *IL6* and *IL1B* at lower concentrations, such as 50 and 100 nM (Fig. 4f). These findings suggest that partial suppression of p16 may be sufficient to alleviate senescence-associated phenotypes. Interestingly, TSN treatment significantly reduced p21 mRNA levels at 200 nM and protein levels at both 100 nM and 200 nM (Supplementary Fig. 3e, f), suggesting that TSN regulates additional senescence pathways beyond p16. However, given that p21 knockdown alone did not restore autophagic flux (Supplementary Fig. 2e,f), a reduction in p21 expression is unlikely to be the primary driver of the autophagy-modulating effect of TSN.

Next, we examined whether TSN affects autophagic flux in both Y and S cells. To specifically investigate the contribution of p16 to TSN-mediated autophagy regulation while minimizing the confounding effects of concurrent p21 suppression, autophagy flux assays were performed using 50 nM TSN. TSN at a concentration of 50 nM significantly increased autophagic flux in S lung fibroblasts, as evidenced by the increased LC3B-I to LC3B-II conversion in the presence of CQ (Fig. 4g). In contrast, there was no evident response in Y cells under the same treatment conditions (Fig. 4h). To determine whether the TSN-induced promotion of autophagic flux in S fibroblasts is mediated by p16, we transfected cells with siRNA targeting p16 and subsequently assessed autophagic flux after TSN treatment. tfLC3 imaging demonstrated that compared with the control treatment, TSN markedly increased the autophagic flux index; notably, compared with the control treatment, TSN could not fully restore the flux index under p16 knockdown conditions, although a modest residual increase was still detected (Fig. 4i, Supplementary Fig. 3g). Moreover, the total cellular RFP/GFP fluorescence intensity ratio remained unchanged (Supplementary Fig. 3h). Importantly, this residual increase was insufficient to further suppress cellular senescence, as determined by SA-β-gal activity (Supplementary Fig. 3i), indicating that the p16-independent component of TSN-mediated autophagy activation does not reach a functional effect. Collectively, these data suggest that TSN enhances autophagic flux at least in part through a p16-dependent pathway.

### TSN exerts antifibrotic effects in vivo, accompanied by changes in senescence and autophagy, with a partial dependence on p16

**Fig. 5:**
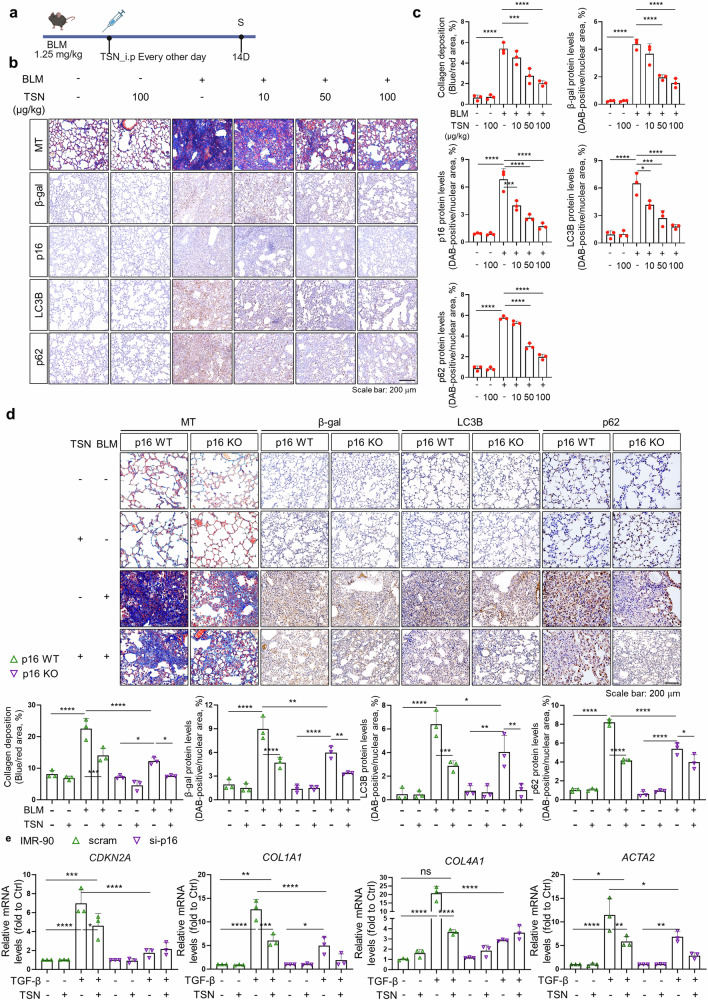
TSN treatment ameliorates fibrosis in a BLM-induced PF mouse model, at least in part through a p16-dependent mechanism. **a** Experimental scheme: PF was induced in B6 or FVB mice via intratracheal BLM administration (1.25 mg/kg), followed by intraperitoneal TSN treatment (10, 50, or 100 µg/kg) every other day for 14 days. **b** Representative lung sections stained with MT, SA-β-gal, and IHC for p16, LC3B, and p62. **c** Quantification of fibrosis-, senescence-, and autophagy-related markers shown in (**b**). **d** Histological comparison of BLM-induced fibrosis with or without TSN (100 µg/kg) in p16 WT and KO FVB mice, along with the quantification of fibrosis, senescence, and autophagy markers. **e** Inhibitory effect of TSN on the upregulation of the expression of TGF-β-induced mRNAs encoding fibrotic genes in IMR90 fibroblasts and the attenuation of its antifibrotic effect in p16 siRNA-treated cells. Data are presented as the mean ± SEM; each dot represents an independent biological replicate. Statistical significance: *p < 0.05, **p < 0.01, ***p < 0.001, ****p < 0.0001

To determine whether the senescence- and autophagy-modulating effects of TSN observed in vitro translate to therapeutic benefits in vivo, we evaluated the antifibrotic efficacy of TSN in a BLM-induced PF mouse model. In earlier experiments, we used p16-KO mice generated on an FVB background to validate the role of p16 in fibrogenesis (Fig. 3f). To further assess the therapeutic potential of TSN, we conducted drug efficacy studies in C57BL/6 (B6) mice, a strain widely used in PF models because of its consistent and robust fibrotic response to BLM. Testing in both the FVB and B6 backgrounds allowed us to evaluate the relevance of p16 and the effect of TSN across genetic contexts. One day after BLM administration, we initiated TSN treatment via intraperitoneal injection every other day at doses of 10, 50, or 100 µg/kg (Fig. 5a). Histological analysis using MT staining confirmed that BLM treatment markedly increased collagen deposition, indicating fibrotic remodeling in the lungs (Fig. 5b, c). TSN treatment significantly reduced collagen deposition at doses of 50 and 100 µg/kg, whereas the 10 µg/kg dose did not significantly affect collagen deposition. In parallel, p16 protein levels were dramatically increased by BLM, whereas TSN treatment significantly reduced levels at doses of 50 and 100 µg/kg, with only a mild reduction observed at 10 µg/kg. To investigate the effects of TSN on cellular senescence and autophagy in vivo, we next measured β-gal protein levels and observed a significant reduction following TSN treatment at 50 and 100 µg/kg. Consistently, the aberrant accumulation of LC3B and p62 proteins in BLM-treated lungs, which is indicative of impaired autophagic flux, was significantly reversed by higher doses of TSN. Overall, while 10 µg/kg TSN mildly reduced BLM-induced p16 expression, it was insufficient to reverse senescence-associated phenotypes or restore autophagic flux. To further validate the effect of TSN on autophagic flux, we conducted western blot analyses focusing on the highest TSN dose, which previously demonstrated significant therapeutic effects. We measured the ratio of LC3B-II to LC3B-I and the levels of p62, a selective autophagy substrate that is normally degraded through autolysosomes under homeostatic conditions. As expected, BLM treatment increased both the LC3B-II/LC3B-I ratio and p62 level, indicating impaired autophagic flux (Supplementary Fig. 4a–c). This was accompanied by a significant increase in the expression of α-SMA, a fibrotic marker (Supplementary Fig. 4d). Notably, treatment with high-dose TSN effectively attenuated the BLM-induced accumulation of LC3B-II/LC3B-I, p62, and α-SMA, confirming its role in restoring autophagic flux. These findings suggest that TSN exerts significant antifibrotic effects in vivo, probably through its combined effects on senescence and autophagy.

In parallel, we investigated the antifibrotic effects of TSN under physiopathological conditions using a BLM-induced senescence model. BLM treatment significantly upregulated the mRNA expression of the cellular senescence effector *CDKN2A* and SASP-associated cytokines such as *IL1A* and *IL6* (Supplementary Fig. 4e). Notably, cotreatment with TSN effectively attenuated these BLM-induced transcriptional changes, collectively suggesting that TSN exerts preventive effects against senescence-associated secretory activation.

To determine whether the antifibrotic effects of TSN are mediated through p16, we next evaluated its efficacy in p16 KO mice subjected to BLM-induced PF. Given that TSN reduced senescence and restored autophagic flux in a p16-dependent manner in vitro, we sought to investigate whether its in vivo effects are similarly dependent on p16. To this end, we administered BLM (1.8 mg/kg) to both p16 WT and KO FVB mice, followed by TSN treatment at the highest dose (100 µg/kg every other day for 2 weeks; Supplementary Fig. 5a). During the treatment period, TSN did not cause any significant changes in body weight (Supplementary Fig. 5b). Histological analyses revealed that TSN treatment attenuated fibrotic remodeling and cellular senescence and restored autophagic flux in both WT and p16-KO mice (Fig. 5d). Notably, although TSN retained antifibrotic efficacy in p16-deficient mice, its reduced potency compared with that in WT mice suggests that TSN exerts its effects at least partially via p16-dependent mechanisms. In parallel, we further analyzed the impact of TSN on the SASP signature in a mouse model of BLM-induced PF (Supplementary Fig. 5c). Both TSN treatment and p16 KO significantly reduced the mRNA levels of SASP cytokines, such as *Il6*, *Il1b*, C-X-C motif chemokine ligand 15 (*Cxcl15*), tumor necrosis factor-α (*Tnf*), and C-C motif chemokine ligand 2 (*Ccl2*), as well as the SASP growth factor, TGF-β1 (*Tgfb1*). In addition, TSN and p16 KO decreased the expression of SASP matrix-remodeling genes, including matrix metallopeptidase-1 (*Mmp1*), matrix metallopeptidase-3 (*Mmp3*), and matrix metallopeptidase-12 (*Mmp12*), in the lung tissues of mice with BLM-induced PF (Supplementary Fig. 5c). Notably, the inhibitory effect of TSN on these SASP genes was markedly reduced under p16-depleted conditions, suggesting that p16 is essential for TSN-mediated suppression of the SASP.

To further examine whether the antifibrotic effects of TSN depend on p16 during fibrogenic changes in fibroblasts and lung epithelial cells, we performed additional in vitro experiments. During TGF-β–induced fibrotic activation in human IMR90 fibroblasts, TSN treatment significantly suppressed the upregulation of *CDKN2A*, collagen type I alpha 1 chain (*COL1A1*), collagen type IV alpha 1 chain (*COL4A1*), α-SMA (*ACTA2*), fibronectin (*FN1*), and vimentin (*VIM*), indicating that TSN inhibits fibroblast-to-myofibroblast transition (FMT; Fig. 5e and Supplementary Fig. 5d). Moreover, p16 knockdown attenuated TGF-β–induced fibrotic changes, and under these conditions, the antifibrotic effect of TSN was abolished and no longer statistically significant, suggesting that p16 modulation is required for TSN activity. Similarly, under p16 knockdown conditions, the inhibitory effects of TSN on the expression of TGF-β–induced senescence-associated markers, such as *IL1A*, *IL1B*, *IL6*, and interleukin-8 *(CXCL8)*, were abolished. Together, these results suggest that the p16-independent component of TSN-mediated autophagy activation (as shown in Fig. 4i) is insufficient to suppress broader fibrogenic and senescence-associated transcriptional programs, whereas p16-dependent pathways are required to achieve functionally meaningful TSN effects targeting both fibrogenic remodeling and senescence-associated phenotypes. Consistently, during fibrogenic changes in lung epithelial cells, such as TGF-β–induced increases in fibronectin and vimentin expression, TSN treatment efficiently blocked these changes, but its inhibitory effects completely disappeared under p16-depleted conditions (Supplementary Fig. 5e). Collectively, although complex in vivo signaling networks may permit partial p16-independent anti-fibrotic and anti-senescence activity (Fig. 5d), our data clearly demonstrate that the antifibrotic and anti-senescence effects of TSN are mediated at least in part through p16.

### p16-dependent control of autophagic flux and expression of fibrotic markers in senescent cells

**Fig. 6:**
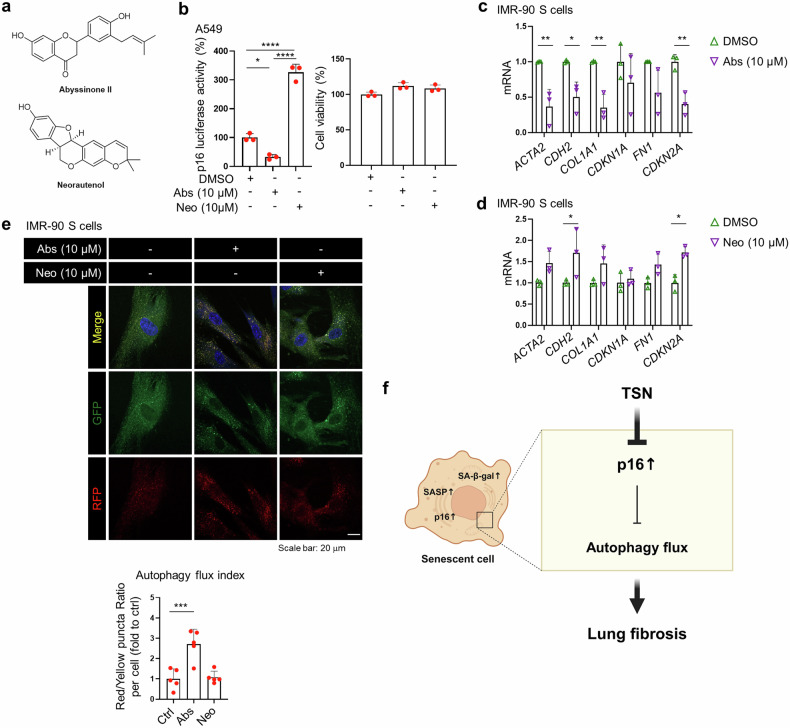
Reciprocal modulation of p16 and fibrosis by small molecules. **a** Chemical structures that were found to change p16 promoter activity (**b**); abyssinone II (Abs) and neorautenol (Neo). RT‒qPCR results showing the effects of (**c**) Abs and (**d**) Neo on the mRNA expression of p16, p21 and fibrosis-associated genes. **e** Representative tfLC3 fluorescence images and quantification showing enhanced autophagic flux in senescent fibroblasts upon Abs treatment, whereas Neo treatment had no effect. Autophagic flux was quantified as the ratio of red (RFP⁺ only) to yellow (RFP⁺/GFP⁺) puncta per cell. **f** Graphical summary: p16 integrates senescence and autophagy, and TSN attenuates fibrosis via p16-targeted pathways. Data are presented as the mean ± SEM; each dot represents an independent biological replicate. Statistical significance: *p < 0.05, **p < 0.01, ***p < 0.001

After confirming that p16 is a functionally relevant target in fibrotic progression, we sought to determine whether p16-mediated regulation of autophagic flux is specific to TSN or is shared by other small molecules that modulate p16 expression. Thus, we next screened a chemical compound library derived from natural products to identify both activators and inhibitors of p16 promoter activity. Through this screen, we identified two additional compounds, abyssinone II (Abs) and neorautenol (Neo), that significantly reduced or increased p16 promoter activity, respectively, without cytotoxic effects at the concentration tested (10 μM; Fig. 6a, b). Although the direct molecular targets of Abs remain undefined, a previous study reported that Abs exhibit an inhibitory effect on human aromatase and are being tested as chemopreventive agents against breast cancer.^43^ Neo is a natural flavonoid isolated from Erythrina species that was previously reported to inhibit neuraminidase and protein tyrosine phosphatase 1B (PTP1B), suggesting potential antibacterial and anticancer activities.^44,45^ Additionally, it has been shown to reduce ERK activation and induce DNA strand breaks in hepatoma cells at low micromolar concentrations.^46^ However, neither compound has previously been reported to regulate p16 expression or exhibit antifibrotic activity, underscoring the novelty of their functional characterization. Thus, to evaluate the functional consequences, we tested these compounds in senescent (S) IMR-90 human fibroblasts. Abs treatment markedly reduced the expression of fibrotic markers, including *ACTA2*, *CDH2*, and *COL1A1*, and significantly decreased p16 mRNA expression (Fig. 6c). In contrast, Neo treatment significantly upregulated the expression of the same fibrotic markers and was accompanied by increased p16 mRNA expression (Fig. 6d). Notably, neither Abs nor Neo affected p21 mRNA, suggesting that p16 modulation may impact a broader spectrum of fibrosis-associated pathways.

To assess autophagic activity, we measured autophagic flux using a tfLC3B construct (Fig. 6e). Abs treatment increased the numbers of both autophagosomes and autolysosomes (Supplementary Fig. 6a), and further analysis revealed a significant increase in the flux index without affecting total cellular acidity (Supplementary Fig. 6b). Together, these findings indicate that Abs enhance autophagic flux. Conversely, Neo treatment had no significant effect on the puncta number or flux index, likely because autophagy was already impaired in senescent cells. Overall, the reciprocal modulation of fibrosis by two structurally distinct p16-targeting compounds, Abs and Neo, reinforces the central role of p16 in governing the senescence–autophagy axis and underscores the broader relevance of our findings.

Taken together, our results validate p16 as a mechanistically actionable driver of fibrotic remodeling and establish a conceptual and experimental framework for discovering diverse chemical modulators beyond the TSN. These potential chemical modulators offer versatile tools for targeting fibrosis through the senescence–autophagy interface (Fig. 6f).

## Discussion

Given the limited efficacy of current IPF therapies, mechanistically grounded antifibrotic approaches are needed. In this study, we established p16 as a mechanistically actionable regulator at the senescence–autophagy interface during fibrosis progression. Through cross-species transcriptomic integration and functional validation, we prioritized p16 over other senescence-associated candidates as the most conserved driver of fibrotic remodeling. Our data demonstrate that p16 elevation constrains autophagic flux in senescent fibroblasts, whereas p16 depletion restores the expression of flux-associated markers and attenuates fibrosis in vitro and in vivo. Leveraging this mechanistic insight, we developed a screening framework that couples p16 promoter activity to autophagy and fibrosis readouts, enabling the discovery of TSN as a dual modulator with robust antifibrotic efficacy. Furthermore, bidirectional validation with additional small molecules confirmed the axis specificity. Collectively, these findings provide a new conceptual and therapeutic framework for selectively targeting senescence-associated autophagy dysfunction in IPF.

In this study, we sought to identify conserved molecular drivers of fibrosis progression by integrating patient-derived transcriptomic datasets with mouse models of pulmonary fibrosis. To ensure transparency and reproducibility, we leveraged open-source transcriptomic resources from the GEO database. Importantly, instead of selecting datasets solely on the basis of sample size, we prioritized IPF cohorts that exhibited clear transcriptional separation between diseased and healthy samples, as assessed by UMAP. This strategy allowed us to extract robust biological signatures, even from smaller cohorts, when the intergroup variance was high and the clustering was well defined, particularly in gene sets related to aging, cellular senescence, the core ECM, and autophagy. Among the senescence-related genes identified through cross-referencing human and mouse fibrosis datasets, *CDKN2A* emerged as the most robust candidate. Although *THBS1* and *CDKN1A* were also consistently upregulated and have been previously implicated in fibrosis,^25–27^ p16 was notable because of its strong positive correlation with core ECM genes upregulated in IPF lungs. In both IPF patient cohorts, p16 expression was most strongly correlated with the fibrotic transcriptome, surpassing those of *THBS1* and *CDKN1A*. This quantitative prioritization supports the idea that p16 plays a more central upstream role in orchestrating fibrotic remodeling. Furthermore, the retrieval of *THBS1* and *CDKN1A*, both of which were previously reported to be associated with fibrosis, through our selection pipeline reinforces the validity of our integrative informatic approach. Rather than relying solely on fold-change or p value thresholds, our correlation-based strategy enabled the identification of functionally relevant targets that are mechanistically aligned with fibrosis biology. To this end, when we extended our analysis to four additional IPF cohorts, the expression of *CDKN2A* consistently increased across all the datasets, whereas the expression of *THBS1* and *CDKN1A* displayed greater variability. This further underscores the robustness of p16 as a conserved marker of fibrotic remodeling. In addition, although this study focused primarily on senescence and autophagy, our unbiased gene set–based comparative framework can be broadly applied to identify upstream regulators and signaling pathways that may drive fibrotic remodeling in IPF. Gene sets commonly altered across IPF cohorts (Supplementary Table 1) provide a list of biological processes that may contribute to IPF progression, along with their corresponding candidate genes. Moreover, applying this comparative strategy separately to each mouse model (IR or BLM) in relation to human IPF data could further delineate model-specific pathways and highlight shared versus divergent mechanisms across models. Through systematic validation using IPF mouse models and patient tissues, this integrative bioinformatics approach revealed additional IPF-driving genes involved in biological processes beyond senescence and autophagy.

Although TSN was administered in a preventive paradigm during BLM-induced fibrosis, our findings provide preliminary insight into its potential therapeutic relevance. This preventive design was intended to elucidate the early mechanistic link between p16-dependent senescence and autophagy during the initiation of fibrotic remodeling. Nevertheless, our in vitro results in which senescent fibroblasts (Fig. 4e, g) and TGF-β–stimulated fibroblasts and epithelial cells (Fig. 5e and Supplementary Fig. 5d) were used indicate that TSN can attenuate profibrotic and prosenescent phenotypes even under conditions of established fibrotic signaling. These observations suggest that TSN may retain antifibrotic efficacy once fibrotic signaling is already active, although dedicated postfibrotic intervention studies are needed to substantiate this possibility. Future investigations employing delayed-treatment models will determine whether TSN can not only prevent but also therapeutically reverse fibrotic remodeling, further strengthening its translational potential.

Although GSEA revealed the upregulation of autophagy-related genes in IPF lungs (Fig. 1b), this cannot be directly interpreted as increased autophagic activity. In contrast to gene sets related to ECM remodeling or cellular senescence, which directly reflect functional pathway activation, the enrichment of autophagy-related genes in IPF lungs requires more cautious interpretation. Notably, increased transcription of autophagy-associated genes might reflect a compensatory transcriptional response to impaired autophagic flux rather than enhanced autophagic activity per se.^47^ For example, previous studies have shown that autophagic flux blockade, particularly during lysosomal degradation, can activate the transcription factor EB (TFEB). Once activated, TFEB translocates to the nucleus and upregulates the expression of autophagy and lysosomal biogenesis genes. These observations underscore the need for functional validation, such as LC3B-II/I ratio analysis or p62 degradation assays, when transcriptomic enrichment of stress-responsive pathways such as autophagy is understood.

We confirmed that p16 knockdown restored autophagic flux in senescent fibroblasts (Fig. 3e) and inhibited fibrotic changes through the promotion of autophagic flux (Fig. 3g); however, the underlying mechanism through which p16 impairs autophagy remains unclear. Notably, although TSN treatment restored autophagic flux in p16-expressing senescent cells, this effect was significantly diminished in p16-depleted cells (Fig. 4i). This functional dependency was further validated through reciprocal pharmacologic manipulation using other compounds, such as Abs and Neo, which downregulated and upregulated p16 expression, respectively (Fig. 6a, b). Treatment with Abs led to p16 suppression, restoration of autophagic flux, and a concomitant reduction in the expression of fibrotic markers (Fig. 6c–e). In contrast, Neo upregulated p16 and elevated the expression of fibrotic markers, although it failed to further reduce autophagic flux in senescent cells, likely because of preexisting impairment of the autophagy pathway. Collectively, these findings underscore the role of p16 as a targetable regulatory hub within the fibrosis signaling network.

In this study, the precise mechanisms by which TSN, Neo, and Abs regulate p16 promoter activity remain unclear. TSN retained partial efficacy even in p16-deficient mice (Fig. 5d), indicating the involvement of p16-independent pathways that act in parallel or additively. These observations align with previous reports showing that TSN modulates multiple signaling cascades, including the PI3K/Akt/mTOR,^48,49^ Nrf2/HO-1, and inflammasome pathways,^50^ each of which play critical roles in oxidative stress and fibrotic remodeling.^51,52^ At the same time, a clear difference was observed between in vivo and in vitro systems: whereas TSN still retained partial antifibrotic, autophagy-associated, and antisenescent effects in p16-deficient mice, depletion of p16 in cell-based models abolished these TSN-mediated effects. Notably, this apparent discrepancy between the in vivo findings and the in vitro observations can be explained by the functional threshold of autophagy activation. Although TSN induced a modest increase in autophagic flux even under p16-deficient conditions in vitro (Fig. 4i), this residual, p16-independent autophagy activation was insufficient to suppress senescence or fibrogenesis, as demonstrated by the loss of antifibrotic and anti-senescent effects in p16-depleted fibroblasts and epithelial cells (Fig. 5e, Supplementary Fig. 5d, 3i). In contrast, the multicellular and injury-responsive in vivo lung environment may permit partial p16-independent antifibrotic efficacy through non-cell-autonomous compensatory mechanisms, thereby allowing TSNs to retain measurable activity in p16-knockout mice (Fig. 5d). However, we confirmed that the inhibitory effects of TSN on fibrogenic changes in human lung epithelial cells and fibroblasts were markedly attenuated upon p16 knockdown (Fig. 5e and Supplementary Fig. 5d). Consistently, the TSN-mediated suppression of SASP markers in the BLM-induced PF mouse model was significantly diminished in the p16-knockout background (Supplementary Fig. 5c). Collectively, these findings indicate that the antifibrotic activity of TSN results from the integrated modulation of interconnected stress‒response networks, within which p16 functions as a key actionable node that links cellular senescence and autophagy.

To identify a possible explanation for p16-mediated autophagy regulation, we conducted a sequence analysis using the eukaryotic linear motif database, which revealed a putative LIR-like motif ([W/F/Y]-X-X-[L/I/V]) located at the C-terminal region of p16 (Supplementary Fig. 7a). Structural modeling suggested that this motif is buried within the α-helical structure of the fourth ankyrin (ANK) repeat domain under basal conditions but can be exposed under stress (Supplementary Fig. 7b). In particular, proximity to the autophagosomal membrane, a condition frequently associated with autophagic activation, can induce local unfolding and β-strand conversion of the LIR-like segment, rendering it competent for LC3 binding. This mechanism resembles the “induced LIR exposure” observed in canonical autophagy cargo receptors. In support of this hypothesis, coimmunoprecipitation experiments revealed that the p16–LC3 interaction was markedly enhanced upon BLM-induced stress in L132 lung epithelial cells (Supplementary Fig. 7c). While minimal interaction was observed under basal conditions, BLM treatment led to increased p16–LC3 binding. These findings suggest that the p16–LC3 interaction represents a disease-induced binding event, is selectively activated under fibrotic stress conditions, and could serve as a novel targetable mechanism at the senescence–autophagy interface. Moreover, TSN not only reduced p16 promoter activity but also attenuated BLM-induced p16–LC3 binding (Supplementary Fig. 8). Although the exact binding pocket of the TSN within the p16–LC3 interface remains to be determined, we are currently investigating this possibility through the development of peptide-based modulators designed to disrupt the interaction.

Taken together, our findings position the senescence–autophagy interface not only as a conceptual framework for understanding fibrotic remodeling but also as a remediable axis with translational potential. By identifying compounds that modulate this axis in opposite directions, we demonstrate a practical toolkit for identifying candidate therapeutics for fibrosis.

## Materials and methods

### Identification of gene expression signatures from public genomic databases

Publicly available gene expression datasets from the GEO database were analyzed to identify transcriptomic alterations in IPF as described in our previous study.^51^ Briefly, to identify conserved transcriptomic alterations in IPF, we analyzed publicly available datasets (GSE53845 and GSE199152) from the GEO database, selected for their clear UMAP-based separation of IPF and control samples. GSEA was performed using fully ranked gene lists and the C2 curated gene set collection (1000 permutations; nominal *p* < 0.05, FDR < 0.05). To prioritize senescence-associated drivers, leading-edge genes from the M9143 senescence signature were cross-referenced with genes upregulated (>2-fold) in two previously characterized PF mouse models (BLM- and IR-induced fibrosis).^53^ Common genes were visualized using a Venn diagram. Correlations between senescence genes (*CDKN2A*, *CDKN1A*, and *THBS1*) and core ECM genes were assessed by Spearman’s rank correlation. The expression of candidate genes was validated in four additional IPF cohorts (GSE17978, GSE47460, GSE10667, and GSE24206). Detailed parameters and gene lists are provided in the Supplementary Materials and Methods.

### Microarray

Total RNA from mouse lung tissues was extracted using an Easy-Spin^TM^ total RNA extraction kit according to the manufacturer’s instructions (iNtRON Biotechnology, Seoul, Republic of Korea). RNAs from 3 mice per group were pooled to exclude experimental bias. Detailed information is provided in the Supplementary Materials and Methods.

### Tissue histology and immunohistochemical staining

Tissue microarrays (US Biomax, Derwood, MD, USA; LC561a) containing human fibrotic (*n* = 16) and normal (*n* = 10) lung samples were used for collagen and protein detection by MT and IHC, following previously described protocols.^51^ Briefly, after standard fixation and paraffin embedding, the sections were stained with MT or probed with antibodies against p16 and LC3B. Images were acquired using Zeiss microscopy systems, and quantitative image analysis was performed using ImageJ software. MT was performed to visualize collagen deposition, with collagen fibers stained blue and noncollagenous tissue stained red. The collagen-to-tissue ratio was quantified by calculating the area of blue-stained regions relative to that of red-stained regions using ImageJ software. IHC staining was quantified by normalizing the DAB-positive area to the nuclear area. Briefly, DAB-positive signals were segmented and quantified using ImageJ software, and the resulting area was divided by the total nuclear area (hematoxylin-stained) within the same field. The normalized values are expressed as a percentage (% of DAB-positive area per nuclear area), as previously described.^54^ Detailed staining procedures are provided in the Supplementary Materials and Methods. All antibodies used in the study are listed in Supplementary Table 7. The tissue slides were sectioned using a semiautomated rotary microtome (RM2245; Leica Biosystems, Wetzlar, Germany) at the Ewha Drug Development Research Core Center (NFEC-2024-03-295118).

### Animal models

All procedures were approved by the Animal Care and Use Committee of Ewha Womans University (IACUC 23–004) and were performed in accordance with the relevant guidelines. B6 mice (males, 8 weeks old; *n* ≥ 3 per group) were purchased from JAbio, Inc. (Seoul, Korea) and maintained in a specific pathogen-free barrier facility under a 12-h light cycle. The mice were randomized into groups before the start of the experiments. In the BLM-induced PF model, mice received a single intratracheal instillation of BLM (Santacruz Biotechnology, Dallas, TX, USA) at 1.8 mg/kg for FVB and at either 1.25 or 2.5 mg/kg for B6 mice. TSN (10, 50, and 100 µg/kg) was intraperitoneally injected every other day for 14 days. FVB (p16 WT and KO) mice were a gift from Professor Nam Ki-Taek at the Department of Biomedical Sciences, Yonsei University College of Medicine.^39^ Briefly, p16^*-*^KO mice were generated by TALEN-mediated targeting downstream of the p16^Ink4a^ start codon to introduce frameshift mutations. Blinding methods were not used for the animal studies in this research.

### Autophagic flux evaluation

Detailed information is provided in the Supplementary Materials and Methods. Briefly, autophagic flux was assessed using three complementary approaches: LC3B conversion, tfLC3 imaging, and p62 degradation. First, for LC3B conversion, the cells were treated with chloroquine (CQ, 25 μM) for 4 h to inhibit autophagosome–lysosome fusion. LC3B-I and LC3B-II protein levels were measured by western blotting, and autophagic activity was quantified by calculating the fold increase in the LC3B-II/LC3B-I ratio between CQ-treated and untreated samples. An increase in LC3B-II accumulation upon CQ treatment was interpreted as an increase in autophagic flux. Second, cells were transfected with tfLC3, in which LC3 is tagged with RFP and green fluorescence protein (GFP) at its N-terminus.^38^ The tfLC3 plasmid (Addgene, Cambridge, MA, cat#:21074) was transfected into IMR90 cells using Lipofectamine 2000 (Invitrogen) according to the manufacturer’s instructions. After treatment, the cells were washed twice with PBS, fixed with 3.7% formaldehyde for 20 min and then washed three times. The nuclei were stained with DAPI. Images were obtained with a confocal microscope (Leica TCS8). Yellow puncta (merged GFP + RFP) indicate autophagosomes, whereas red puncta (RFP-only) indicate autolysosomes. We quantified (i) the number of yellow puncta (autophagosomes), (ii) the number of red puncta (autolysosomes), and (iii) the autophagy flux index (red/yellow puncta ratio) as described in previous reports.^55–57^ Puncta detection and classification were performed using Image-Pro Premier™ (Media Cybernetics, MD, USA) with the preset puncta-counting algorithm, which automatically identifies individual puncta and distinguishes RFP-only (red) from GFP + RFP (yellow) signals, followed by per-cell normalization of puncta counts. Third, p62/SQSTM1 protein levels were measured by western blotting as an indirect marker of autophagic flux. Given that p62 is selectively degraded by autophagy, reduced p62 levels were interpreted as an indication of enhanced autophagic degradation. The antibodies used for western blotting are listed in Supplementary Tables 8 and 9. Fluorescence imaging was performed using a fluorescence microscope (Axio Observer 7, Carl Zeiss, Oberkochen, Germany) at the Ewha Drug Development Research Core Center (NFEC-2021-08-272462).

### RNA extraction and quantitative real-time polymerase chain reaction

NucleoZOL (Macherey-Nagel) was used to extract total RNA, and the RNA concentration and purity were measured using a NanoDrop ND-2000 spectrophotometer (Thermo Fisher Scientific, Waltham, MA, USA) at the Ewha Drug Development Research Core Center (NFEC-2023-03-286005). For cDNA synthesis, 1 µg of total RNA was processed with an M-MLV Reverse Transcriptase kit (Bioneer, Korea). The reaction was conducted in a 20 µL volume and then diluted to 60 µL, which was used as a template in the following real-time qPCRs. Real-time qPCR was performed using AccuPower® 2X GreenStar™ qPCR Master Mix (Bioneer). All the reactions were performed in triplicate. The expression levels of all the markers were normalized to that of 18S ribosomal RNA. The primer information is included in Supplementary Table 10. Real-time qPCR was conducted using a CFX96 Touch Real-Time PCR System (Bio-Rad Laboratories Inc., Hercules, CA, USA) at the Ewha Drug Development Research Core Center (NFEC-2021-08-272451).

### Compound extraction and isolation

Dried *Melia azedarach* fruits (1.8 kg) were extracted with 70% ethanol and then sequentially partitioned with n-hexane, ethyl acetate (EtOAc), and n-butanol. The EtOAc fraction, which exhibited the strongest activity in the p16 promoter assay, was subjected to multistep chromatographic separation using silica gel, reversed-phase C18, Sephadex LH-20, and semipreparative HPLC to isolate five bioactive compounds, including TSN. After solvent evaporation, all the fractions and isolated compounds were redissolved in DMSO and subsequently used for biological assays conducted under identical vehicle conditions. Detailed purification protocols and compound structures are provided in the Supplementary Materials and Methods.

### Cell culture

IMR-90 human lung fibroblasts (ATCC, CCL-186) were cultured in MEM supplemented with 10% FBS, 100 U/mL penicillin, and 100 μg/mL streptomycin under standard humidified conditions (37 °C, 5% CO₂). Cells were passaged at a 1:8 ratio, and population doubling levels (PDLs) 7–11 were defined as young, whereas PDLs 36–40 were defined as replicative senescent fibroblasts. L132 human lung epithelial cells (ATCC, CCL-5) were cultured in Roswell Park Memorial Institute medium supplemented with 10% fetal bovine serum and 1% penicillin‒streptomycin. A549 lung epithelial cells (KCLB) were maintained in RPMI 1640 supplemented with 10% FBS and antibiotics. To induce BLM-induced senescence, A549 cells were treated with 5 μg/mL BLM for 4 days. The detailed protocols are described in the Supplementary Materials and Methods.

### Cell viability assay

Cell viability was assessed using the 3-(4,5-dimethylthiazol-2-yl)-2,5-diphenyltetrazolium bromide (MTT) assay, as described in the Supplementary Materials and Methods. The absorbance was measured using a multifunctional microplate reader (Spark, Tecan Group Ltd., Mannedorf, Switzerland) at the Ewha Drug Development Research Core Center (NFEC-2024-09-299675).

### Senescence-associated β-galactosidase (SA-β-gal) staining

Cells were stained using a Senescence β-Galactosidase Staining Kit (#9860, Cell Signaling Technology, Danvers, MA) according to the manufacturer’s instructions. After staining, the cell samples were observed with a microscope (Olympus IX70; Olympus Corporation, Tokyo, Japan).

### p16 promoter activity-related luciferase reporter assay

To assess p16 promoter activity, a luciferase reporter construct driven by the human p16 promoter (−722 to −180) was generated and transfected into A549 cells. Luciferase activity was measured after compound treatment and normalized to cell viability, as determined by the MTT assay, to account for potential differences in the number of viable cells. The detailed cloning, transfection, and assay conditions are described in the Supplementary Materials and Methods.

### Western blotting

Protein lysates were extracted using RIPA buffer supplemented with protease and phosphatase inhibitors. Equal amounts of protein were resolved by SDS‒PAGE, transferred to PVDF membranes, and incubated with primary and HRP-conjugated secondary antibodies. The protein bands were visualized by chemiluminescence and quantified using ImageJ software. Detailed buffer compositions, antibody information, and detection conditions are provided in the Supplementary Materials and Methods. The total list of antibodies used for western blotting can be found in Supplementary Tables 8 and 9. Western blot images were visualized using a chemiluminescence imaging system (GBOX CHEMI XX9; Syngene, Cambridge, UK) at the Ewha Drug Development Research Core Center (NFEC-2022-12-283636).

### Statistical analysis

All the data were analyzed using GraphPad Prism 10 (GraphPad Software, La Jolla, California, USA). For comparisons between two groups, the data were assessed for a normal distribution and analyzed using Student’s *t* test. One-way ANOVA was used for multiple-group comparisons, followed by Tukey’s post hoc test for all pairwise comparisons or Dunnett’s post hoc test when multiple groups were compared with a single positive control. Pearson’s correlation coefficients (*r*) were calculated to evaluate the linear relationships between variables. All data were obtained from a minimum of three independent biological replicates; each dot represents an independent biological replicate. The data are presented as the mean ± SEM. Statistical significance was defined as *P* < 0.05 (*), *P* < 0.01 (**), *P* < 0.001 (***), and *P* < 0.0001 (****).

## Supplementary information

Supplementary Materials

List of gene sets upregulated and downregulated in IPF versus normal lungs from IPF patient cohorts

List of leading-edge genes in senescence gene set (M9143)

List of up-regulated genes in pulmonary fibrosis mouse models

Correlation analyses of CDKN1A, CDKN2A, and THBS1

Other Supplementary Materials for this manuscript: Uncropped western blots

## Data Availability

All the protocols, statistical analyses, and other relevant materials used in the current study are available within the paper and its Supplementary Information. The datasets generated and analyzed during the current study are not publicly available because of ethical and regulatory restrictions, but may be made available from the corresponding author upon reasonable request.
