# Supplementary material for: Targeting the senescence‒autophagy axis via p16INK4a inhibition alleviates pulmonary fibrosis
Source: Signal Transduct Target Ther. 2026 Jun 15;11:234. doi: 10.1038/s41392-026-02730-4 (PMC13270108; doi:10.1038/s41392-026-02730-4)
Supplement: Supplementary file 1 — Supplementary Materials [file 41392_2026_2730_MOESM1_ESM.pdf]

Supplementary Materials for

Targeting the senescence-autophagy axis via p16^{INK4a} inhibition alleviates pulmonary fibrosis

Young Jo Yoo[#], Mi Zhang[#], Seung-Hyun Jin[#], Yae Rin Lee, Hee Jin, Thi-Phuong Doan, Sumin Kim, Seri Choi, Eun Jin Park, Sanga Na, Yeonjin Lee, Jiawei Sun, Jaeho Cho, Nam-Chul Cho, So-Yeon Park[*],Won-Keun Oh[*], Yun-Sil Lee[*]

[#,] *These authors contributed equally to this work

Correspondence to: Yun-Sil Lee, yslee0425@ewha.ac.kr

This PDF file includes:

Materials and Methods
Supplementary Figures 1 to 6
Supplementary Tables 1 to 10

Other Supplementary Materials for this manuscript include the following:

Uncropped western blots

Materials and Methods

Identification of gene expression signatures from public genomic database

Publicly available gene expression datasets from the GEO database were analyzed to identify transcriptomic alterations in IPF as described in our previous study. [1] Briefly, two independent IPF cohorts were selected based on their clear transcriptional separation between disease and control samples, as visualized by UMAP: GSE53845 and GSE199152. UMAP was generated by using GEO R2 webserver (https://www.ncbi.nlm.nih.gov/geo/geo2r/). Raw expression matrices, obtained from GEO R2, were processed to include all annotated genes, rather than differentially expressed gene (DEG) subsets. These full ranked gene lists were used as input for GSEA using the Java implementation of the Broad Institute's GSEA software (https://www.gsea-msigdb.org/gsea/index.jsp). Analyses were performed using the C2 curated gene set collection, with 1,000 permutations, a minimum gene set size of 15, and a maximum of 500. Pathway enrichment was considered statistically significant if it met both of the following criteria: nominal p-value < 0.05 and FDR q-value < 0.05. For gene-level correlation analysis, expression values of senescence-associated genes (*CDKN2A*, *CDKN1A*, *THBS1*) were correlated with core ECM genes using Spearman's rank correlation, implemented in R. To identify conserved upstream regulators, leading-edge genes from the M9143 senescence gene set, enriched in each human IPF cohort, were intersected with upregulated genes from two previously published PF mouse models: BLM-induced and IR-induced pulmonary fibrosis, which have been reported in our previous study by microarray. [1] Genes that were upregulated by more than 2-fold in fibrotic mice compared to naïve controls were classified as upregulated. The overlapping genes across human and mouse datasets were visualized using a four-way Venn diagram generated via an online tool (https://bioinformatics.psb.ugent.be/webtools/Venn/). The expression patterns of three candidate genes were validated using additional IPF cohorts available at GEO (GSE17978, GSE47460, GSE10667, GSE24206)

Microarray

Total RNA from the mouse lung tissues was extracted using the Easy-SpinTM total RNA extraction kit according to the manufacturer's instructions (iNtRON Biotechnology, Seoul, Republic of Korea). Isolated total RNA was amplified and labeled using the Low RNA Input Linear Amplification kit PLUS (Agilent Technologies) and hybridized to a microarray containing approximately 44,000 probes (~ 21,600 unique genes), in accordance with the manufacturer's instructions (Agilent Mouse whole genome 44K, Agilent Technologies). The arrays were scanned using an Agilent DNA Microarray Scanner (Agilent Technologies). Analysis was performed by Ebiogen Inc. (Seoul, Republic of Korea).

Tissue histology and immunohistochemical staining

Tissue microarrays (TMAs) containing pulmonary interstitial fibrosis samples were obtained from US Biomax (Derwood, MD, USA; LC561a). This array includes 10 normal and 16 fibrotic lung tissue samples. To assess the presence of specific proteins and collagen within these tissues, we conducted immunohistochemistry (IHC) and collagen staining analyses based on previously established protocols. [1] Briefly, collected tissues were fixed in 10% neutral buffered formalin and subsequently processed into paraffin blocks. Tissue sections were cut from these blocks, deparaffinized, and subjected to standard staining procedures using hematoxylin and eosin (Sigma-Aldrich, St. Louis, MO, USA), Masson's trichrome (Sigma-Aldrich), or picrosirius red (Polysciences, Warrington, PA, USA; #24901–500) to visualize collagen structures. For immunohistochemical staining, antigen retrieval was performed by boiling the slides in 0.1 M citrate buffer (pH 6.0) for 20 minutes. Endogenous peroxidase activity was blocked by treating the slides with 0.3% hydrogen peroxide in methanol for 15 minutes. Non-specific binding was minimized by incubating the sections with normal goat serum at 37°C for one hour. Slides were then incubated overnight at 4°C with primary antibodies targeting P16 (1:200, Proteintech) and LC3B (1:200, Santa Cruz Biotechnology). Signal detection was achieved using the avidin-biotin-peroxidase complex method (ABC kit; Vector Laboratories) followed by visualization with 3,3′-diaminobenzidine (Zymed Laboratories, Montrouge, France). Counterstaining was performed with hematoxylin. The stained sections were examined under a light microscope (Carl Zeiss, Jena, Germany)

or a Zeiss Apotome microscope (Carl Zeiss) installed at the Ewha Drug Development Research Core Center, and the positive staining areas were quantitatively analyzed using ImageJ software (NIH, Bethesda, MD, USA) based on standard image processing algorithms. All antibodies used in the study were listed in Supplementary Table 6.

Animal models

All procedures were approved by the Animal Care and Use Committees of the Ewha Womans University (IACUC 23–004) and were performed in accordance with the relevant guidelines. C57BL/6J (male, 8 weeks old, n≥3 per group) were purchased from JAbio Inc (Seoul, Korea), maintained in a specific pathogen-free barrier facility under a 12-hour light cycle. The mice were randomized into groups before starting experiments. For a BLM-induced PF model, mice received a single intratracheal instillation of BLM at 1.8 mg/kg for FVB, and 1.25 or 2.5 mg/kg for B6 mice (Santacruz Biotechnology, Dallas, TX, USA). TSN (10, 50 and 100 µg/kg) were intraperitoneally injected every other day for 14 days. FVB (p16 WT and KO) mice were gifted by Prof Professor Nam Ki-Taek at the Department of Biomedical Sciences, Yonsei University College of Medicine. [2] Briefly, p16 KO mice were generated by TALEN-mediated targeting a downstream of the $p16^{Ink4a}$ start codon to introduce frameshift mutations.

Autophagic flux evaluation

Autophagic flux was assessed using three complementary approaches: LC3B conversion, tfLC3 imaging, and p62 degradation. First, for LC3B conversion, cells were treated with chloroquine (CQ, 10 µM) for 4 hours to inhibit autophagosome–lysosome fusion. LC3B-I and LC3B-II protein levels were measured by Western blotting, and autophagic activity was quantified by calculating the fold increase in the LC3B-II/LC3B-I ratio between CQ-treated and untreated samples. An increase in LC3B-II accumulation upon CQ treatment was interpreted as enhanced autophagic flux. Second, cells were transfected with tfLC3, in which LC3 is tagged with red fluorescence protein (RFP) and green fluorescence protein (GFP) at its N-terminus. [3] tfLC3 plasmid (Addgene, Cambridge, MA, cat#:21074) was transfected into IMR90 cells using lipofectamine 2000 (Invitrogen) according to the manufacturer's instructions. After treatment, the cells were washed twice with PBS and fixed with 3.7% formaldehyde for 20 min and repeated wishing three times. The nuclei were stained with DAPI. Images were obtained with a confocal microscope (Leica TCS8). Yellow puncta (merged GFP+RFP) indicated autophagosomes, while red puncta (RFP-only) indicated autolysosomes. The total fluorescence intensity per cell for each channel was quantified to evaluate autophagosome formation and autolysosomal maturation. Third, p62/SQSTM1 protein levels were measured by Western blotting as an indirect marker of autophagic flux. Since p62 is selectively degraded by autophagy, reduced p62 levels were interpreted as an indication of enhanced autophagic degradation. The antibodies used in Western blotting were listed in Supplementary Tables 7 and 8.

Compound extraction and isolation

The dried fruits of *Melia azedarach* (1.8 kg) were extracted with 70% EtOH three times at room temperature. The combined extracts were evaporated under reduced pressure, suspended in water, and successively partitioned with *n*-hexane, EtOAc, and *n*-BuOH, respectively. The EtOAc-soluble fraction (13 g) was subjected to medium-pressure liquid chromatography (MPLC) on a reversed-phase (RP) C18 column, eluted with a $MeOH/H_2O$ gradient system, to afford seven subfractions (M1− M7). Among them, fraction M1 exhibited the strongest biological activity and was further separated on a Sephadex LH-20 column with 100% MeOH, yielding four subfractions (M1L1−M1L4). Fraction M1L2 was subsequently chromatographed on RP-C18 chromatography using a gradient of $MeOH/H_2O$ (20/80 → 100/0, v/v), producing 18 subfractions (R1-R18). Subfraction R10 was repeatedly chromatographed on Sephadex LH-20, yielding seven fractions (L1-L7). Fraction L5 was then purified by HPLC (CH_3CN/H_2O = 63:37, v/v) to yield compounds **4** (6.9 mg) and **5** (2.0 mg). Similarly, subfraction R16 was chromatographed on Sephadex LH-20 and further purified by semi-preparative HPLC (CH_3CN/H_2O = 63:37, v/v) to yield compound **1** (70.0 mg). Fraction M2, which showed moderate activity, was also subjected to Sephadex LH-20 column chromatography to give four subfractions

(M2L1-M2L4). Fraction M2L2 was further separated on a silica gel column using n-hexane/EtOAc (4:1 → 0:1, v/v) as the eluent to produce six fractions (N1-N6). Finally, fraction N5 was purified by semi-preparative HPLC (CH_3CN/H_2O = 63:37, v/v) to yield compounds **2** (4.2 mg) and **3** (4.5 mg).

Physicochemical properties of isolated compound 1

Toosendanin (TSN): White amorphous powder; $[\alpha]_D^{25} = -31$ (*c* 0.75, MeOH); UV (MeOH) λ_{max} (log ε) 208 (1.70); IR (KBr) ν_{max} 3560, 1750, 1510, 880 cm^{-1}; ^{1}H-NMR (400 MHz, methanol-d_4) and ^{13}C NMR (100 MHz, methanol-d_4): Tables 5 and 6; HRESIMS: found *m/z* 597.2418 $[M + Na]^+$ (calcd for $C_{30}H_{38}O_{11}Na$, 597.2414).

Trichilinin (**2**): White amorphous powder; $[\alpha]_D^{25} = +11$ (*c* 0.005, MeOH); UV (MeOH) λ_{max} (log ε) 195 (1.70), 280 (0.57); IR (KBr) ν_{max} 3409, 2932, 2853, 1731, 1598, 1436, 1377, 1249, 1160, 1077, 1027, 954, 870 cm^{-1}; ^{1}H-NMR (600 MHz, methanol-d_4) and ^{13}C NMR (150 MHz, methanol-d_4): Tables 5 and 6; HRESIMS: found *m/z* 551.2650 $[M + Na]^+$ (calcd for $C_{30}H_{41}O_8Na$, 551.2639).

Meliatoosenin P (**3**): White solid; $[\alpha]_D^{25} = +40$ (*c* 0.005, MeOH); UV (MeOH) λ_{max} (log ε) 206 (1.70); IR (KBr) ν_{max} 3423, 2932, 1717, 1647, 1436, 1377, 1259, 1146, 1027, 949, 875, 732 cm^{-1}; ^{1}H-NMR (600 MHz, methanol-d_4) and ^{13}C NMR (150 MHz, methanol-d_4): Tables 5 and 6; HRESIMS; found *m/z* 547.2678 $[M + Na]^+$ (calcd for $C_{31}H_{40}O_7Na$, 547.2672).

12-hydroxy, 29-*exo*-amoorastatone (**4**); White amorphous powder; $[\alpha]_D^{25} = +35$ (*c* 0.005, MeOH); IR (KBr) ν_{max} 3399, 2932, 2366, 2312, 1726, 1662, 1377, 1165, 1052, 1023, 949, 875, 796 cm^{-1}; ^{1}H-NMR (600 MHz, methanol-d_4) and ^{13}C NMR (150 MHz, methanol-d_4): Tables 5 and 6; HRESIMS found *m/z* 532.2274 $[M + H]^+$ (calcd for $C_{28}H_{37}O_{10}$, 532.2308)

12-hydroxy, 29-*endo*-amoorastatone (**5**); White amorphous powder; $[\alpha]_D^{25} = +21$ (*c* 0.005, MeOH); UV (MeOH) λ_{max} (log ε) 209 (1.48), 292 (1.70); IR (KBr) ν_{max} 3379, 2927, 2375, 2317, 1726, 1668, 1613, 1377, 1298, 1259, 1165, 1141, 1023, 949, 870, 791, 702 cm^{-1}; ^{1}H-NMR (400 MHz, methanol-d_4) and ^{13}C NMR (100 MHz, methanol-d_4): Table 6; HRESIMS found *m/z* 532.2274 $[M + H]^+$ (calcd for $C_{28}H_{37}O_{10}$, 532.2308).

Cell culture

Human lung fibroblast (IMR 90; ATCC, CCL-186) cells were obtained from the American Type Culture Collection (ATCC) and maintained in Minimum Essential Medium (MEM) supplemented with 10% fetal bovine serum (FBS; Gibco, Grand Island, NY, USA), 100 U/mL penicillin, and 100 μg/mL streptomycin (Hyclone, GE Healthcare Life Sciences, UK) in a humidified incubator with 5% CO2 at 37 °C. The cells were routinely subcultured at a 1:8 ratio until they failed to reach confluency within 3 weeks. Young IMR90 fibroblasts were used at population doubling levels (PDL) 7-11, while replicatively senescent cells were used at PDL 36-40. The human lung carcinoma epithelial cell line A549 (type II alveolar epithelial-like cells) was obtained from the Korean Cell Line Bank (Seoul, Korea) and maintained in RPMI-1640 medium (Welgene, Deajeon, Korea) supplemented with 10% FBS (Gibco), 100 U/mL penicillin, and 100 μg/mL streptomycin (Hyclone) under the same culture conditions. L132 human pulmonary epithelial cells were purchased from ATCC (CCL-5) and maintained in Eagle's Minimum Essential Medium supplemented with 10% FBS.

MTT assay

The MTT assay was performed to measure cell viability. Briefly, proliferating or senescent cells were seeded into 96-well plates. After treatment, the medium was discarded, and 50 μL of MTT (3-[4,5-dimethylthiazol-2-yl]-2,5-diphenyl tetrazolium bromide) solution (500 μg/mL in serum-free medium) was added to each well. After 4 h incubation, the MTT solution was removed, and the resulting formazan crystals were dissolved in DMSO, and the absorbance was measured at 495 or 570 nm using a microplate reader.

Senescence-associated β-galactosidase (SA-β-gal) staining

Cells were stained by using Senescence β-galactosidase staining Kit (#9860, Cell Signaling Technology, Denver, MA) according to the manufacturer's instructions. After staining, the cells were observed under a light microscope (Olympus ix70, Olympus Corporation, Tokyo, Japan).

p16^{INK4a} promoter activity related luciferase reporter assay

To generate a reporter construct of the human p16^{INK4A} promoter (GeneBank Accession No. NM_000077.4), a 430-bp DNA fragment including the promoter of the human p16INK4A gene (-722 to -180) was PCR-amplified using genomic DNA from 293T as a template, nPfu-Forte DNA polymerase (Enzynomics, Daejeon, Korea), and the primer pair 5'-CCCGGTACCGTGGAAGAAAAGGGGAGGAG-3' (KpnI site) and 5'-CCCCTCGAGCCGGACTAGGTAGGTGGAGTC-3' (XhoI site). The PCR product was digested with KpnI and XhoI and cloned into the pGL3-Basic luciferase reporter vector (Promega, Madison, WI, USA) to generate the pGL3_p16^{INK4A} promoter construct. The recombinants plasmid was transformed into *Escherichia coli* and confirmed by DNA sequencing. A549 cells (8,000 per well) were seeded in 96-well plates, and after 24 h of incubation, the medium was replaced with a transfection mixture containing 0.07 μg of pGL3-p16^{INK4A} promoter plasmid (encoding firefly luciferase driven by the p16^{INK4A} promoter), 0.03 μg of RSV-β-galactosidase plasmid (internal standard), and 0.2 μL of Lipofectamine 2000 (Invitrogen, Carlsbad, CA, USA) in 20 μL Opti-MEM. An additional 20 μL of Opti-MEM was added to ensure full medium coverage. After 4 h of transfection, the medium was replaced with RPMI-1640 containing 10% FBS, and the cells were incubated overnight. The cells were then treated with vehicle, navitoclax (positive control), or test compounds for 24 h before measuring luciferase and β-galactosidase activities. Cell lysates were prepared using Cell Culture Lysis Buffer (Promega, WI, USA), and the Firefly Luciferase Assay Kit (Promega) was used to assess luciferase activity. The luciferase activity was normalized to cell viability, determined by MTT assay, to account for potential differences in the number of viable cells.

Western Blotting (WB)

Proteins were extracted using RIPA buffer containing 1% protease inhibitor cocktail (Roche Diagnostics, Basel, Switzerland). After protein quantification (Bio-Rad Protein Assay, Hercules, CA, USA), the samples were denatured in SDS sample buffer at 95 °C for 5 minutes. Protein samples were separated by gradient SDS-polyacrylamide gel electrophoresis (SDS-PAGE; Nacalai Tesque, USA) and transferred onto a polyvinylidene difluoride (PVDF) membrane (Millipore, Billerica, MA, USA). The membranes were blocked with 5% skim milk and incubated overnight at 4 °C with specific primary antibodies. After washing, membranes were incubated with HRP-conjugated secondary antibodies (Cell Signaling Technology), and protein bands were visualized using ECL detection reagent (AB Frontier) with an ImageQuantTM LAS 4000 Imaging System (GF Healthcare). Band intensities were quantified using ImageJ software (GE Healthcare).

For tissue samples, lung tissue was homogenized and lysed in RIPA buffer (50 mM Tris-HCl [pH 7.5], 150 mM NaCl, 1% Nonidet P-40, 0.1% SDS, and 1% sodium deoxycholate) supplemented with 1 mM Na 3 VO 4, 1 mM DTT, 1 mM NaF, and a protease/phosphatase inhibitor cocktails (GenDEPOT, TX, USA; Roche Applied Science, Penzberg, Germany). After centrifugation at 13,000 rpm for 30 min, the supernatant was collected, and protein concentration was determined by Bradford assay (Bio-Rad, CA, USA). Equal amounts of protein were separated by 6-12% SDS-PAGE, transferred, and blocked with 5% skim milk or 5% BSA (in PBS). The membranes were incubated with primary antibodies overnight at 4 °C and visualized using enhanced chemiluminescence (EzWestLumi, Tokyo, Japan). Protein bands were detected with a ChemiDoc Imaging System (Bio-Rad) and quantified using ImageJ software 1.45 (NIH). A complete list of antibodies is provided in Supplementary Tables 8 and 9.

Supplementary Figure 1

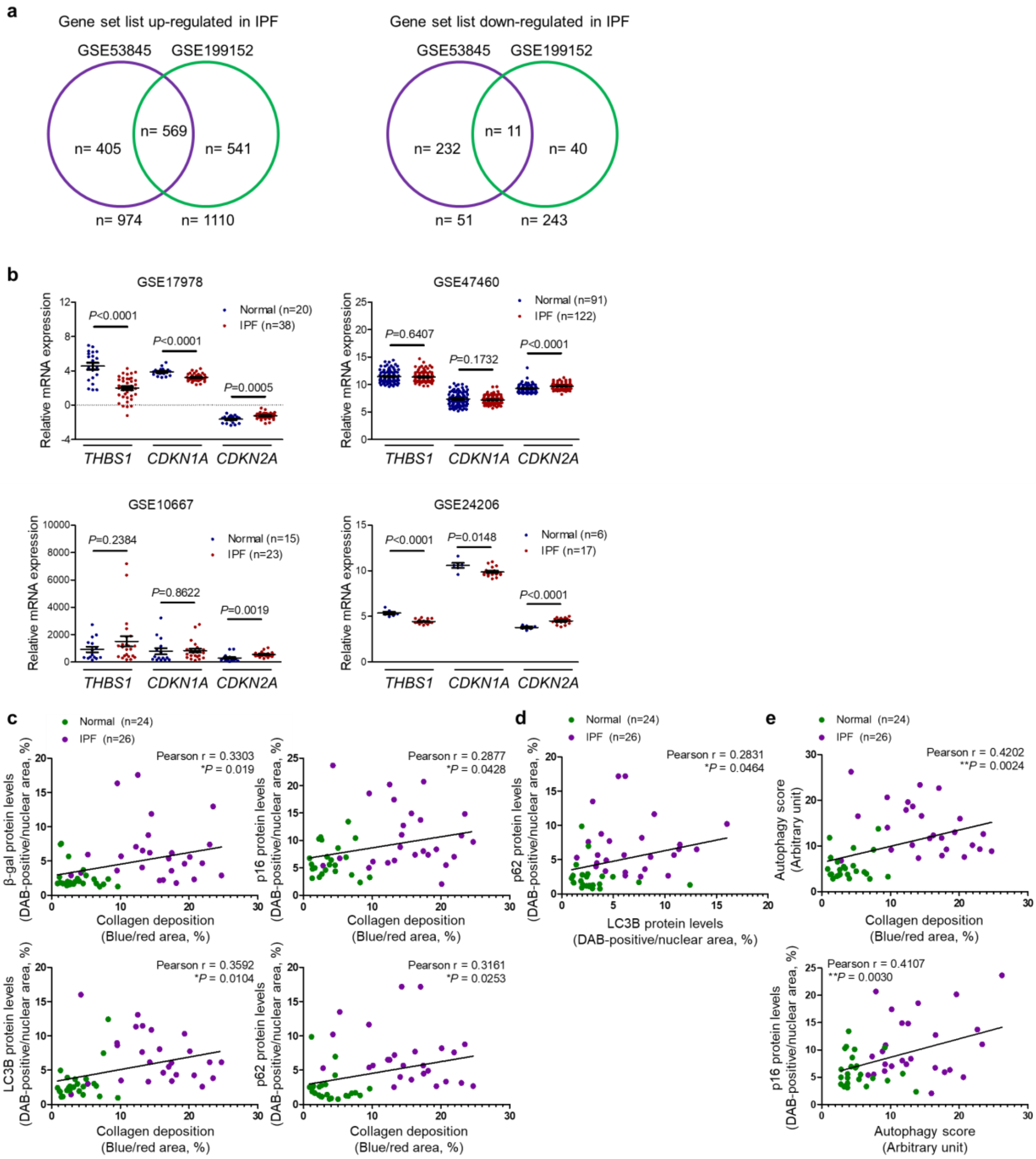

Supplementary Fig. 1. Validation of p16 as a fibrosis-associated gene in human IPF cohorts.

(a) Venn diagrams showing the overlap of up-regulated and down-regulated gene sets in lung tissues from IPF patients, obtained from gene set enrichment analyses of GSE53845 and GSE199152 datasets. (b) Scatter plots of gene expression for *THBS1*, *CDKN1A*, and *CDKN2A* across four additional IPF cohorts (GSE17978, GSE47460, GSE10667, GSE24206), confirming consistent upregulation of *CDKN2A* in fibrotic lungs. (c-e) Correlation plots showing Pearson's correlation coefficients (R) and p-values based on histological analyses of lung tissues from IPF patients. The autophagy score represents the sum of p62 and LC3B protein levels (percentage of stained area). Data are presented as mean ± SEM; each dot represents an independent biological replicate.

Supplementary Figure 2

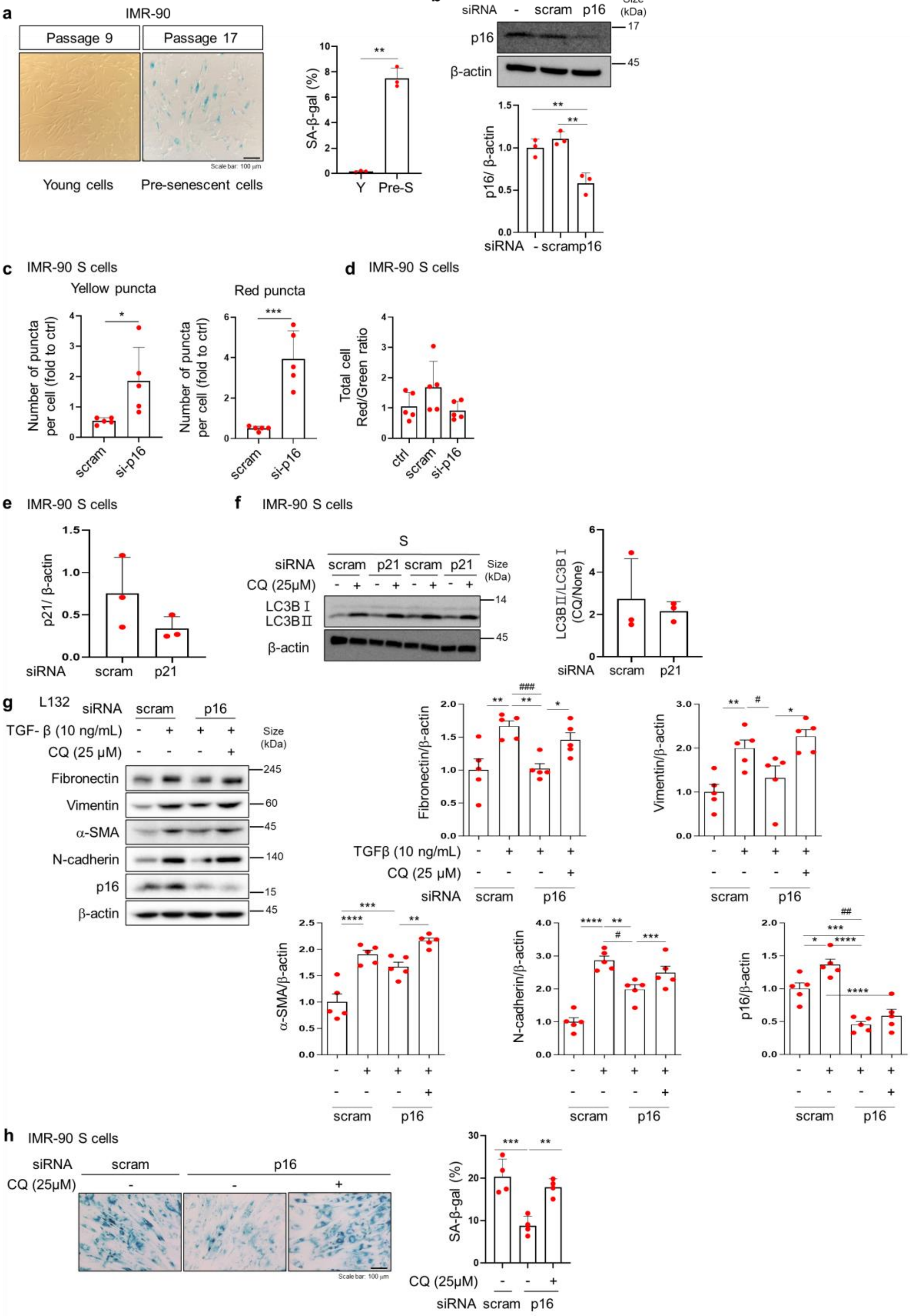

Supplementary Fig. 2. Validation of senescence status and p16/p21 knockdown status in IMR90 fibroblasts.

(a) Representative SA-β-gal staining images (left) of young (Y, PD9) and pre-senescent (pre-S, PD17) IMR90 fibroblasts. Quantification (right) of SA-β-gal–positive area shows a marked increase in senescent cells in pre-S fibroblasts. (b) Western blotting confirming efficient knockdown of p16 using siRNAs in senescent (S) and pre-S IMR90 fibroblasts. Quantification of p16 normalized to β-actin. (c) Quantification of the total number of yellow (RFP^+/GFP^+) puncta and red (RFP^+ only) puncta per cell shown in Fig. 3e. (d) Graph indicates the red-to-green (RFP/GFP) ratio across all cells shown in Fig. 3e. (e) Validation of p21 protein levels after transfection with siRNAs targeting p21. (f) Validation of CQ-mediated LC3B-II/I conversion assay following p21 knockdown shows no significant restoration of autophagic flux. Western blot images of LC3B-I and LC3B-II in senescent (S) fibroblasts treated with CQ (25 μM) with or without p21 knockdown. (g) Effect of autophagic flux blockade on the antifibrotic activity of p16 knockdown. CQ was used to inhibit autophagic flux during TGF-β–induced fibrogenic stimulation to evaluate whether blocking autophagy could reverse the antifibrotic effects of p16 knockdown. (h) p16 knockdown decreases SA-β-gal activity in S cells, indicating reduced senescence, and this reduction is reversed by autophagic flux blockade with CQ. Data are presented as mean ± SEM; each dot represents an independent biological replicate. * denotes statistical significance among all experimental groups, whereas # denotes significance between the control and p16 siRNA-treated groups. Statistical significance: $*,^{\#}p < 0.05$, $**,^{\#\#}p < 0.01$, $***,^{\#\#\#}p < 0.001$.

Supplementary Figure 3

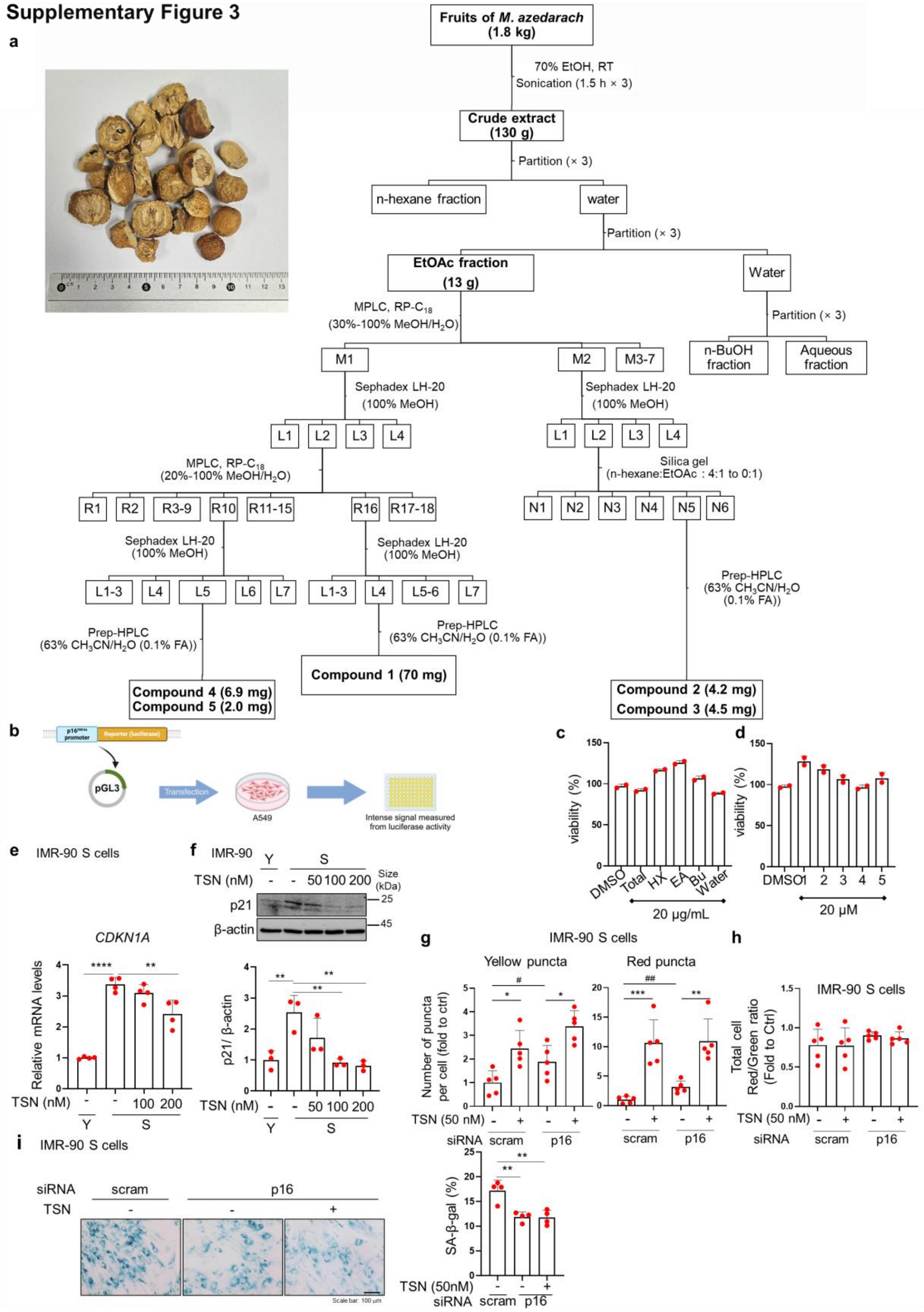

Supplementary Fig. 3. Purification scheme and p16 promoter-based screening of *Melia azedarach*–derived fractions and compounds.

(a) Photograph of dried *Melia azedarach* fruits used for extraction (left). Schematic workflow (right) of solvent partitioning, chromatographic separation, and purification of the EtOAc fraction leading to isolation of five compounds (#1–5). (b) Schematic diagram of the p16 promoter-driven luciferase reporter assay pipeline used to screen natural fractions and isolated compounds. (c) Luciferase assay results showing p16 promoter activity after treatment with samples from different solvent fractions and subfractions. (d) Screening of individual purified compounds identified compound #1 (TSN) as the most potent inhibitor of p16 promoter activity (e and f) Effect of TSN treatment on p21 (*CDKN1A*) expression at (e) mRNA and (f) protein levels in senescent (S) fibroblasts. (g) Quantification of the total number of yellow (RFP^+/GFP^+) puncta and red (RFP^+ only) puncta per cell shown in Fig. 4i. (h) Graph indicates the red-to-green (RFP/GFP) ratio across all cells shown in Fig. 4i. (i) TSN treatment decreases SA-β-gal activity in S cells, and this reduction is abolished by p16 knockdown. Data are presented as mean ± SEM; each dot represents an independent biological replicate. Statistical significance: *$p < 0.05$, **$p < 0.01$, ***$p < 0.001$, ****$p<0.0001$.

Supplementary Figure 4

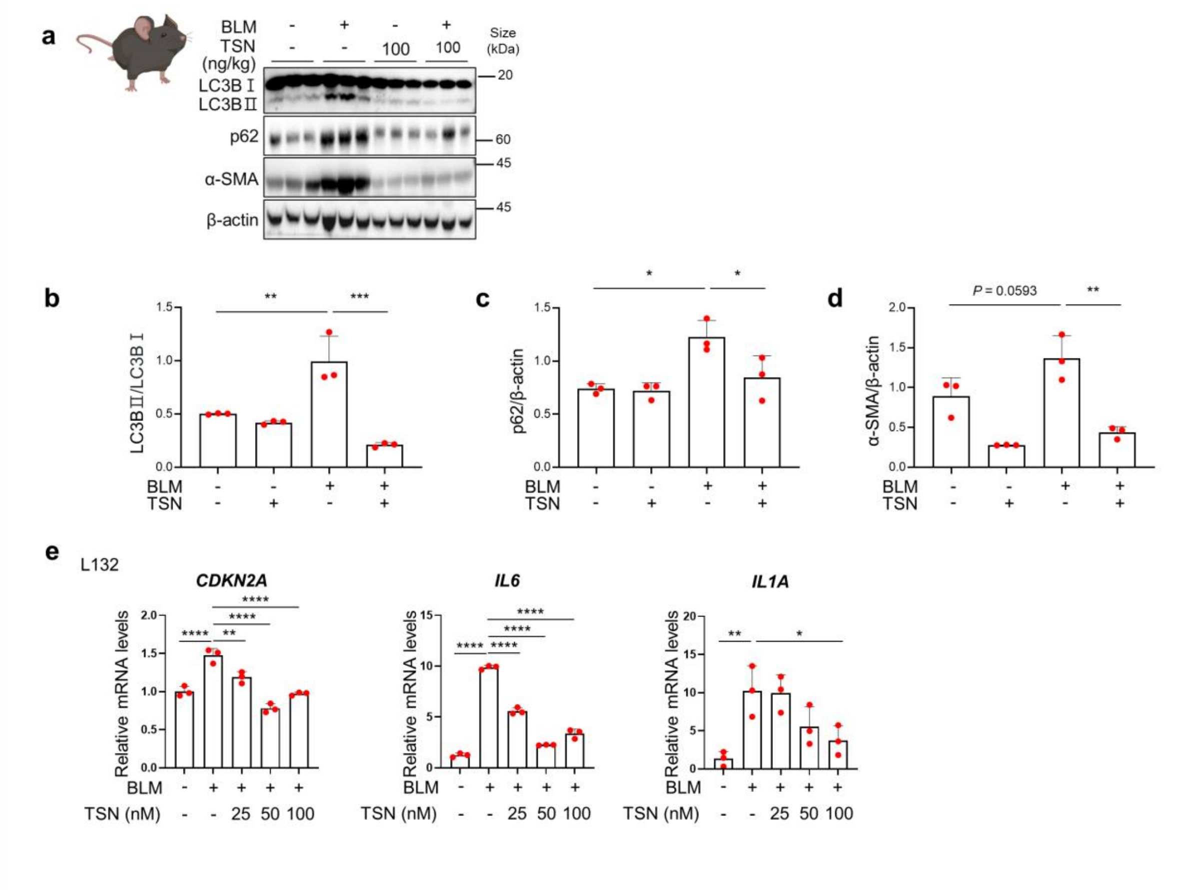

Supplementary Fig. 4. Autophagy and fibrosis markers in BLM-treated mice following TSN intervention, and TSN-mediated modulation of BLM-induced cellular senescence

(a) Representative immunoblots showing protein levels of LC3B-I/II, p62, and α-SMA in lung tissues from mice treated with BLM and increasing doses of TSN (10, 50, 100 μg/kg) or vehicle control. β-actin was used as a loading control. (b-d) Quantification of protein band intensities for LC3B-II/I ratio (b), p62 (c), and α-SMA (d). (e) L132 human pulmonary epithelial cells were treated with BLM for 24 hours. TSN was co-treated with BLM to evaluate its preventive effect on BLM-induced cellular senescence. Data are presented as mean ± SEM; each dot represents an independent biological replicate. Statistical significance: *p < 0.05, **p < 0.01, ***p < 0.001, ****p < 0.0001.

Supplementary Figure 5

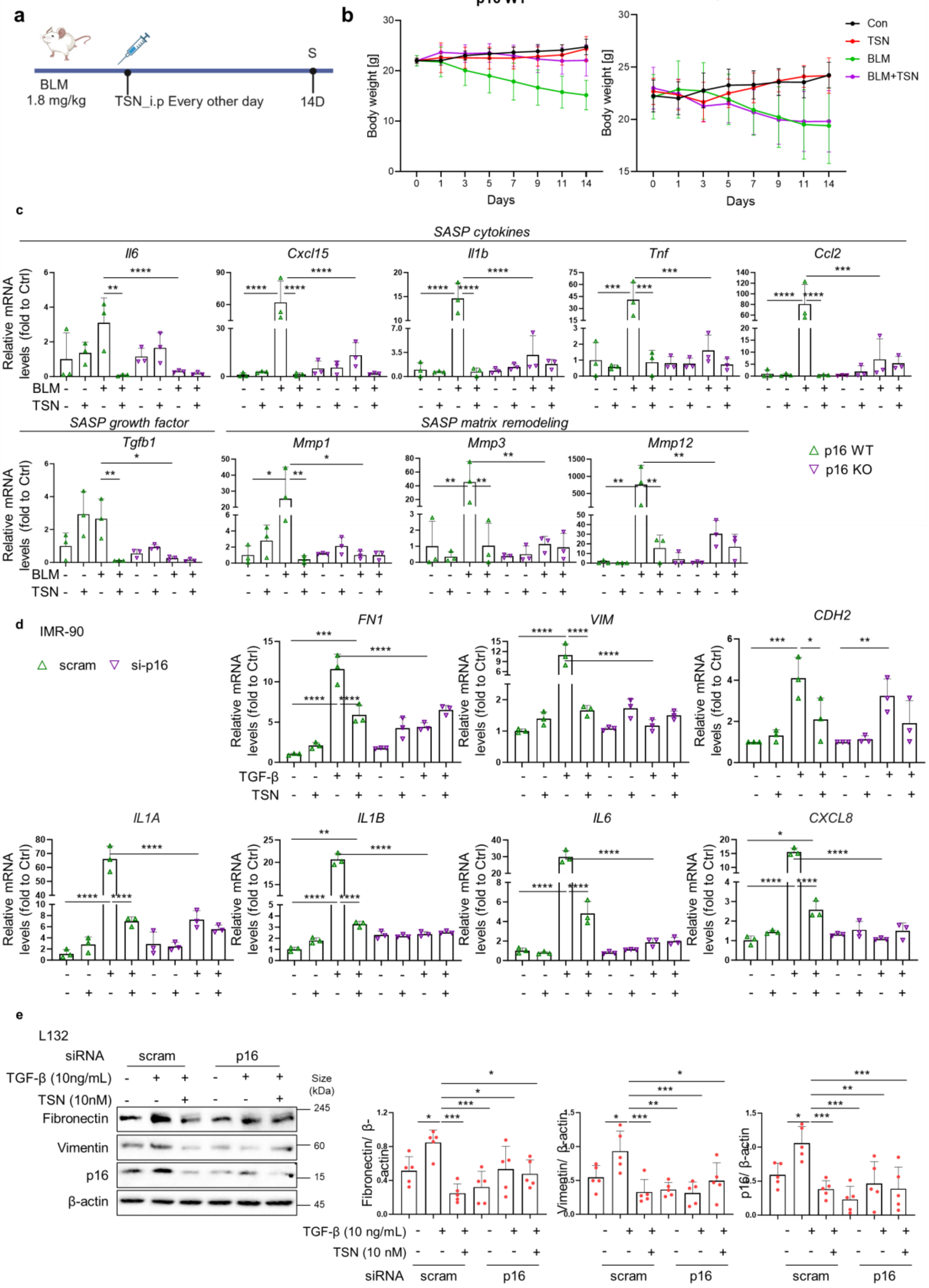

Supplementary Fig. 5. TSN does not cause significant weight loss in p16 WT and KO mice, and suppresses BLM-induced SASP expression in vivo and TGF-β–induced fibrogenic markers in L132 cells.

(a) Schematic of the experimental timeline: bleomycin (BLM, 1.8 mg/kg) was administered intratracheally to both p16 WT and KO FVB mice to induce PF. TSN (100 μg/kg) was injected intraperitoneally every other day for 14 days. (b) Body weight changes monitored throughout the experiment. No significant weight loss was observed in TSN-treated groups, indicating acceptable tolerability. WT and KO groups showed similar trends in weight change upon TSN administration. (c) Both TSN treatment and p16 knockout significantly reduced SASP-associated cytokines (Il6, *Cxcl8*, *Il1b*, *Tnf*, *Ccl2*), growth factor (*Tgfb1*), and matrix-remodeling genes (*Mmp1*, *Mmp3*, *Mmp12*) in lung tissues of BLM-induced PF mice. The inhibitory effect of TSN was attenuated in p16 KO mice, indicating that TSN suppresses SASP partly through a p16-dependent pathway. (d) Inhibitory effect of TSN on TGF-β-induced mRNA upregulation of fibrotic genes and SASP-associated genes in IMR90 fibroblasts, and attenuation of its antifibrotic effect in p16 siRNA-treated cells. (e) L132 cells transfected with scrambled or p16-targeting siRNAs were treated with TGF-β for 24 hours with or without TSN co-treatment. Target protein levels were analyzed by western blotting. Data are presented as mean ± SEM; each dot represents an independent biological replicate. Statistical significance: *$p < 0.05$, **$p < 0.01$, ***$p < 0.001$, ****$p < 0.0001$.

Supplementary Figure 6

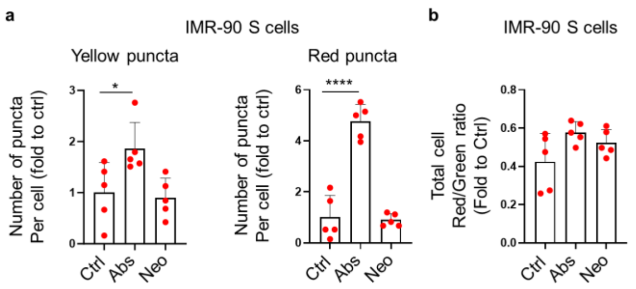

Supplementary Fig. 6. Evaluation of abyssinone II (Abs) and neorautenol (Neo) effects on autophagic flux using the tandem fluorescent LC3 (tfLC3) reporter.

(a) Quantification of the total number of yellow (RFP^+/GFP^+) puncta and red (RFP^+ only) puncta per cell shown in Fig. 6e. (b) Graph indicates the red-to-green (RFP/GFP) ratio across all cells shown in Fig. 6e. Data are presented as mean ± SEM; each dot represents an independent biological replicate. Statistical significance: *$p < 0.05$ and ****$p < 0.0001$.

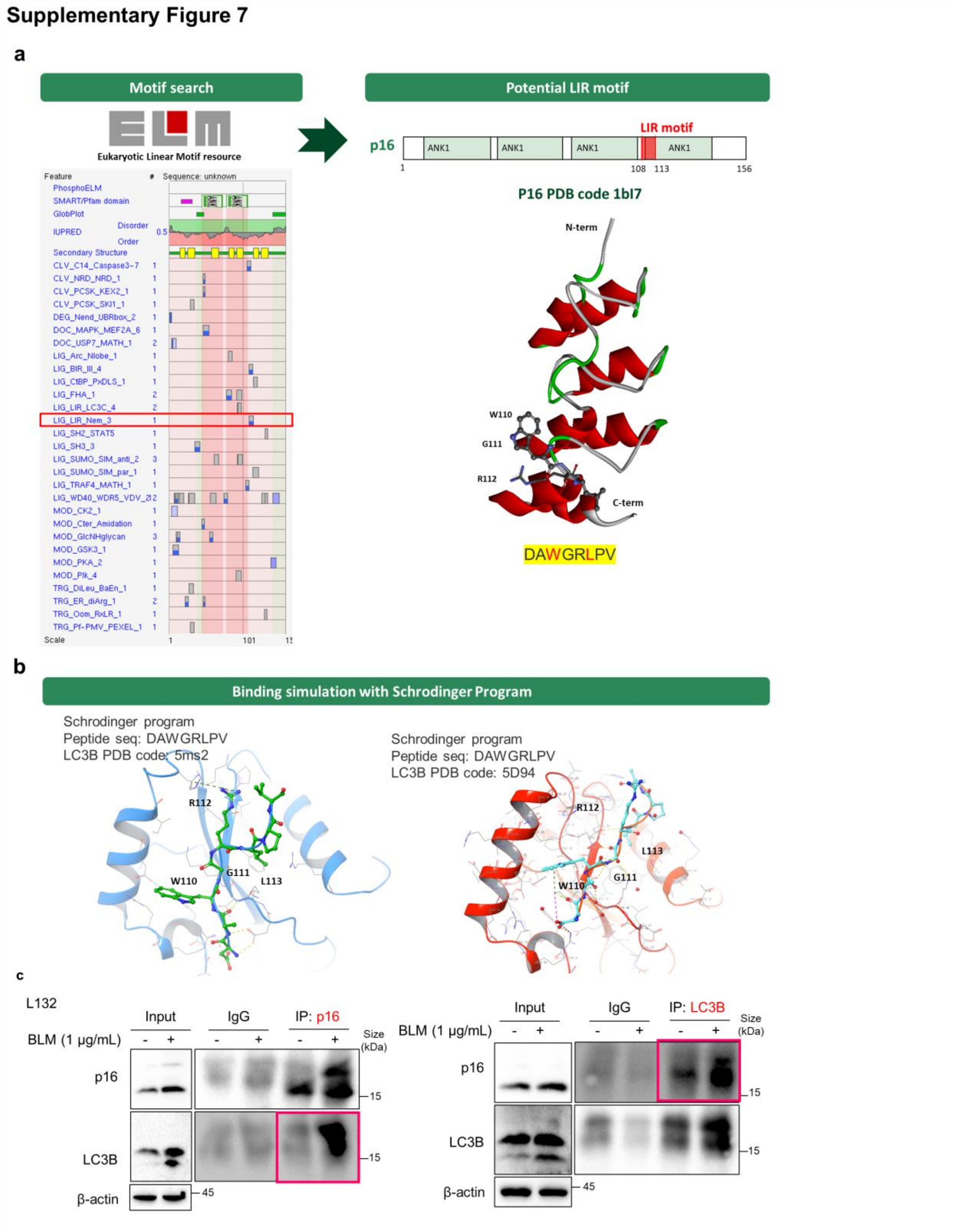

Supplementary Fig. 7. *In silico* and experimental evidence supporting p16–LC3B interaction through a putative LIR motif.

(a) Motif search using the ELM database identified a potential LC3-interacting region (LIR)-like motif in the C-terminal region of p16. (b) Structural modeling (PDB ID: 1bi7) revealed that this motif resides within the ankyrin repeat domain and is structurally buried under basal conditions. Binding simulation using Schrödinger software predicted favorable docking of the putative LIR motif with the LC3B hydrophobic pocket (LC3B PDB codes: 5ms2 and 5D94), with W110, G111, and L113 as key contact

residues. (c) Co-immunoprecipitation (co-IP) assays showing enhanced interaction between p16 and LC3B in BLM-treated L132 lung epithelial cells, supporting stress-induced p16–LC3B binding. Boxed bands highlight BLM-induced co-IP signals.

Supplementary Figure 8

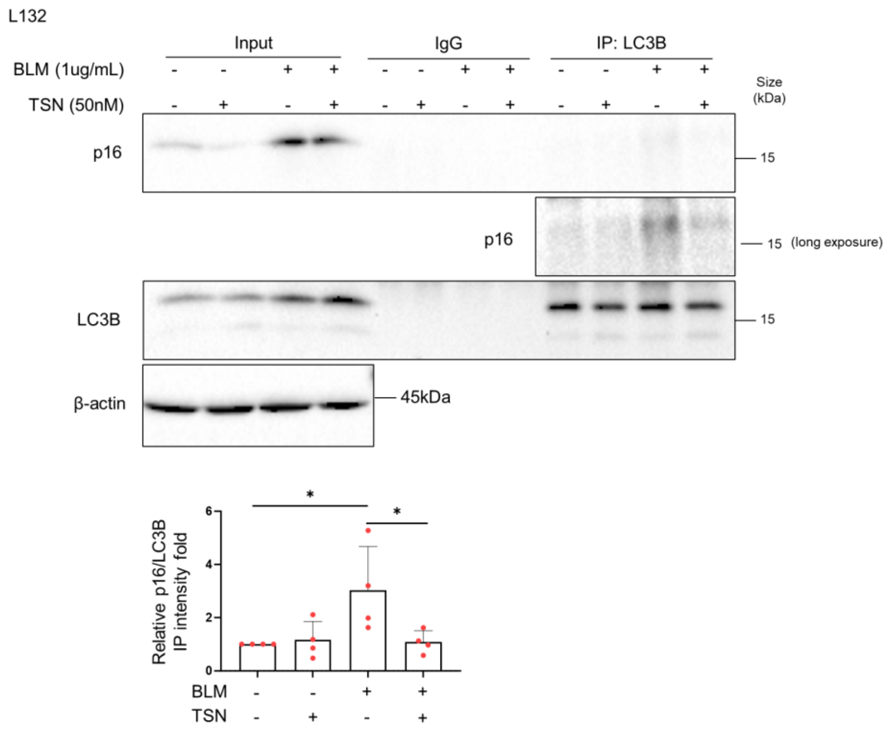

Supplementary Fig. 8. Inhibitory effect of TSN on BLM-induced LC3B–p16 interaction in L132 cells

L132 cells were co-treated with bleomycin (BLM) and TSN, followed by co-immunoprecipitation (co-IP) as described in Fig. 7c. TSN markedly decreased the interaction between LC3B and p16.

Supplementary Table 1. List of gene sets upregulated and downregulated in IPF versus normal lungs from IPF patient cohorts

Attached as a separate excel file.

Supplementary Table 2. List of leading-edge genes in senescence gene set (M9143)
Attached as a separate excel file.

Supplementary Table 3. List of up-regulated genes in pulmonary fibrosis mouse models.
Attached as a separate excel file.

Supplementary Table 4. Correlation analyses of *CDKN1A*, *CDKN2A*, and *THBS1*.
Attached as a separate excel file.

Supplementary Table 5. ^{1}H NMR data of Compounds 1, 2, and 3 (δ_H in ppm, *J* in Hz)

Position	**1** (MeOD-*d4*)[b]		**2** ($CDCl_3$)[a]		**3** ($CDCl_3$)[b]	
	δ_C	δ_H *m* (J in Hz)	δ_C	δ_H *m* (J in Hz)	δ_C	δ_H *m* (J in Hz)
1	70.9	4.26, m	74.2	3.59, d (7.6)	73.3	5.06, t (2.5)
2	37.2	2.80, dd (16.2, 3.7)<br>1.81, d (16.2)	30.2	2.30, dt (16.2, 3.2)<br>1.97, dt (16.2, 2.8)	30.8	2.05, m<br>2.22, d (3.0)
3	74.8	5.30, d (4.1)	72.0	5.07, t (1.6)	70.8	3.90, s
4	41.2	-	42.6	-	44.3	-
5	29.6	2.54, t (13.6)	38.6	2.42, d (12.3)	38.9	2.72, dd (12.3, 2.4)
6	26.2	1.72, m<br>2.02, m	73.8	4.16, dd (12.3, 3.1)	72.6	4.02, dt (12.1, 2.9)
7	70.7	3.56, m	72.6	4.21, d (2.9)	85.9	4.21, d (2.0)
8	42.9	-	45.4	-	48.7	-
9	50.1	4.69, s	34.9	2.88, dd (12.3, 7.7)	38.3	2.51, d (11.2)
10	43.8	-	40.4	-	40.7	-
11	209.1	-	24.1	2.18, dt (12.0, 3.2)<br>1.48, dd (13.9, 7.7)	41.1	2.33, d (2.7)<br>2.18, br s
12	79.7	5.34, s	77.9	5.09, d (2.8)	199.5	9.11, dd (4.9, 2.6)
13	46.9	-	51.8	-	136.7	-
14	73.6	-	157.7	-	145.7	-
15	59.9	3.82s	122.8	5.69, dd (3.2, 1.4)	88.0	5.24, t (7.4)
16	34.8	2.00, m<br>2.12, dd (13.4, 6.4)	36.9	2.48, dd (7.9, 4.5)<br>2.56, dd (11.0, 1.3)	42.0	2.14, m<br>2.29, d(3.1)
17	39.8	2.88, dd (11.0, 6.4)	50.4	3.04, dd (10.8, 7.9)	49.6	3.60, s
18	20.9	1.37, s	15.6	1.11, s	13.7	1.59, s
19	65.4	4.30, d (12.8)<br>4.25, d (12.8)	15.8	0.93, s	15.5	0.98, s
20	124.2	-	124.8	-	126.9	-
21	143.7	7.40, s	140.4	7.23, s	138.5	7.13, s
22	113.0	6.16, s	111.9	6.27, br d (1.6)	110.1	6.10, s
23	142.0	7.19, s	142.2	7.35, t (1.6)	143.4	7.34, br t (2.3)
24	172.2	-	171.2	-	-	-
25	21.4	1.97, s	21.5	1.91, s	-	-
26	172.9	-	169.8	-	-	-
27	23.1	2.08, s	21.3	2.12, s	-	-
28	19.9	0.85, s	78.2	3.45, d(7.5)	77.5	3.64, dd (7.1, 4.3)<br>4.15, d (8.5)
29	97.3	4.84, overlapped	19.3	1.19, s	20.0	1.16, s
30	15.8	1.13, s	27.2	1.12, s	17.0	1.26, d (7.0)
1’	-	-	-	-	166.2	
2’	-	-	-	-	128.4	
3’	-	-	-	-	139.3	6.93, dd (14.0, 7.1)
4’	-	-	-	-	12.4	1.79, d (7.0)
5’	-	-	-	-	14.7	1.87, s

[a] Measured in 600 MHz for ^{1}H and 150 MHz for ^{13}C NMR.
[b] Measured in 400 MHz for ^{1}H and 100 MHz for ^{13}C NMR.

Supplementary Table 6. ^{1}H NMR data of Compounds 4 and 5 (δ_H in ppm, *J* in Hz)

Position	**4** (MeOD-*d4*)[a]		**5** (MeOD-*d4*)[b]	
	δ_C	δ_H *m* (J in Hz)	δ_C	δ_H *m* (J in Hz)
1	71.5	4.57, d (4.2)	71.4	4.51, d (4.5)
2	37.2	2.72, d (3.8)<br>1.84, d (16.0)	37.2	2.72, d (3.8)<br>1.84, d (16.0)
3	74.9	5.21, d (3.8)	74.9	5.14, d (4.1)
4	41.0	-	41.0	-
5	29.3	2.80, dd (14.0, 3.1)	29.3	2.80, dd (14.0, 3.1)
6	24.0	1.72, dt (14.0, 3.9)	24.0	1.72, dt (14.0, 3.9)
7	70.8	4.08, d (1.7)	70.6	4.08, d (1.7)
8	42.7	-	42.7	-
9	49.0	3.67, d (16.8)	49.0	3.67, d (16.8)
10	43.7	-	43.7	-
11	214.0	-	214.0	-
12	78.8	4.26, s	78.8	4.26, s
13	47.5	-	47.5	-
14	59.5	3.41, dd (15.1, 1.5)	59.5	3.41, dd (15.1, 1.5)
15	220.7	-	220.7	-
16	46.3	2.72, d (9.4)<br>2.54, ddd (17.1, 7.2, 1.9)	46.3	2.72, d (9.4)<br>2.54, ddd (17.1, 7.2, 1.9)
17	39.7	3.49, dd (16.0, 9.0)	39.7	3.49, dd (16.0, 9.0)
18	22.1	0.93, d (6.5)	22.1	0.93, d (6.5)
19	65.0	4.21, d (12.3)<br>4.15, d (12.3)	65.0	4.15, d (12.3)<br>4.08, d (12.3)
20	126.3	-	126.3	-
21	141.5	7.36, s	141.5	7.36, s
22	111.9	6.38, s	111.9	6.38, s
23	144.2	7.44, t (1.8)	144.2	7.44, t (1.8)
24	-	-	-	-
25	-	-	-	-
26	172.8	-	172.9	-
27	21.3	2.07, d (4.8)	21.3	2.07, d (4.8)
28	19.9	0.85, s	19.9	0.85, s
29	97.2	4.85, s	97.2	4.80, s
30	22.2	1.15, s	22.2	1.15, s

[a] Measured in 600 MHz for ^{1}H and 150 MHz for ^{13}C NMR.
[b] Measured in 400 MHz for ^{1}H and 100 MHz for ^{13}C NMR.

Supplementary Table 7. List of antibodies, their source and working dilutions in immunohistochemistry and immunofluorescence.

Antibody	Staining	Source	Host Species	Working Concentration
α-SMA	IF	Sigma, A5228	Mouse	1:200
LC3B	IHC, IF	CST, #2775	Rabbit	1:100
p16	IHC, IF	SC-1661	Rabbit	1:100
β-gal	IHC	SC-377257	Mouse	1:100
p62	IHC	Invitrogen, MAB8028	Mouse	1:100

Supplementary Table 8. List of antibodies, their source and working dilutions in immunoblotting for *in vitro* samples

Antibody	Source	Host Species	Working Concentration
LC3B	CST #2775	Rabbit	1:4000
p16	Invitrogen, PA1-30670	Rabbit	1:500
p21	Invitrogen, AHZ0422	Mouse	1:200
β-actin	Santa Cruz Biotechnology, sc-E1012	Mouse	1:5000
Fibronectin	BD Biosciences, 610077	Mouse	1:1000
N-cadherin	Santa Cruz Biotechnology, sc-271386	Mouse	1:1000
α-SMA	Sigma-Aldrich, A5228	Mouse	1:1000
Vimentin	CST #5471	Rabbit	1:1000

Supplementary Table 9. List of antibodies, their source and working dilutions in immunoblotting for *in vivo* samples

Antibody	Source	Host Species	Working Concentration
LC3B	CST #2775	Rabbit	1:1000
p62	Invitrogen, MAB8028	Mouse	1:1000
α-SMA	Sigma, A5228	Mouse	1:1000
β-actin	Santa Cruz Biotechnology, sc-47778	Mouse	1:1000

Supplementary Table 10. Primer sequences for the qRT-PCR

Gene	Species	Primers 5'-3'
ACTA2	human	F: CCGACCGAATGCAGAAGGA
		R: ACAGAGTATTTGCGCTCCGCA
CDH2	human	F: CCTCCAGAGTTTACTGCCATGAC
		R: GTAGGATCTCCGCCACTGATTC
COL1A1	human	F: GTGCGATGACGTGATCTGTGA
		R: CGGTGGTTTCTTGGTCGGT
CDKN1A	human	F: ATATGCCTTCCCCCACTACC
		R: CGTGAGTGCTCACTCCAGAA
FN1	human	F: GAAGCCGAGGTTTTAACTGC
		R: CCCACTCGGTAAGTGTTCC
CDKN2A	human	F: ATGAAATTCACCCCCTTTCC
		R: CCCTAGGCTGTGCTCACTTC
18S	human	F: GTAACCCGTTGAACCCCATT
		R: CCATCCAATCGGTAGTAGCG
IL1A	human	F: ATCAGTACCTCACGGCTGCT
		R: TGGGTATCTCAGGCATCTCC
IL1B	human	F: CTGTCCTGCGTGTTGAAAGA
		R: TTCTGCTTGAGAGGTGCTGA
IL6	human	F: AGGAGACTTGCCTGGTGAAA
		R: CAGGGGTGGTTATTGCATCT
CXCL8	human	F: GTGCAGTTTTGCCAAGGAGT
		R: CTCTGCACCCAGTTTTCCTT
Il6	mouse	F: GAG GAT ACC ACT CCC AAC AGA
		R: AAG TGC ATC ATC GTT GTT CAT ACA
Cxcl15	mouse	F: GAT GCT CCA TGG GTG AAG G
		R: TCT CCC GAA TTG GAA AGG G
Il1b	mouse	F: CTG GTG TGT GAC GTT CCC ATT A
		R: CCG ACA GCA CGA GGC TTT
Tnf	mouse	F: AGG GTC TGG GCC ATA GAA CT
		R: CCA CCA CGC TCT TCT GTC TAC
Tgfb1	mouse	F: CAC TCCCGT GGC TTC TAG TG
		R: GCG GGT GAC CTC TTT AGC AT
Mmp1	mouse	F: ATG GCA TCC AAA GAG GTG ACA
		R: CTG TTG GCA CGT AAC TGC G
Mmp3	mouse	F: TTA AAG ACA GGC ACT TTT GGC G
		R: CCC TCG TAT AGC CCA GAA CT
Mmp12	mouse	F: CAT GAA GCG TGA GGA TGT AGA C
		R: TGG GCT AGT GTA CCA CCT TTG
Ccl2	mouse	F: TAA AAA CCT GGA TCG GAA CCA AA
		R: GCA TTA GCT TCA GAT TTA CGG GT
Gapdh	mouse	F: AGG TCG GTG TGA ACG GAT TTG
		R: TGT AGA CCA TGT AGT TGA GGT CA

Supplementary References

1 Jin, H. *et al.* GTSE1-driven ZEB1 stabilization promotes pulmonary fibrosis through the epithelial-to-mesenchymal transition. *Mol Ther.* **32**, 4138-4157 (2024).

2 Kim, K. H. *et al.* Sexually dimorphic leanness and hypermobility in p16 Ink4a/CDKN2A-deficient mice coincides with phenotypic changes in the cerebellum. *Sci Rep.* **9**, 11167 (2019).

3 Mizushima, N. & Murphy, L. O. Autophagy assays for biological discovery and therapeutic development. *Trends Biochem Sci.* **45**, 1080-1093 (2020).
