# Supplementary material for: Targeting the senescence‒autophagy axis via p16INK4a inhibition alleviates pulmonary fibrosis
Source: Signal Transduct Target Ther. 2026 Jun 15;11:234. doi: 10.1038/s41392-026-02730-4 (PMC13270108; doi:10.1038/s41392-026-02730-4)
Supplement: Supplementary file 6 — Other Supplementary Materials for this manuscript: Uncropped western blots [file 41392_2026_2730_MOESM6_ESM.pdf]

Figure 3 a, N=1

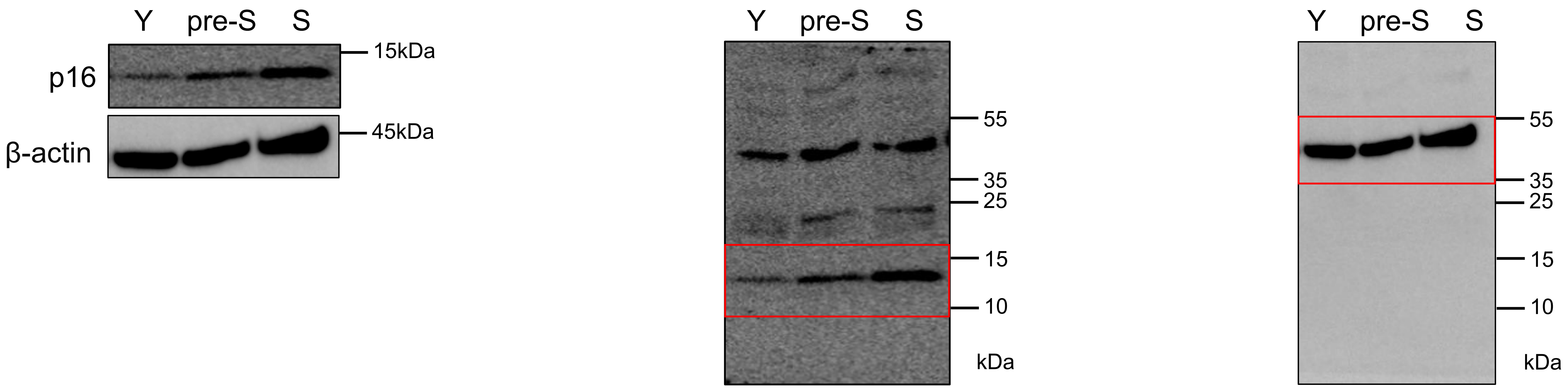

IB: p16 (16kDa) **IB: β-actin (45kDa)**

Figure 3 a, N=2

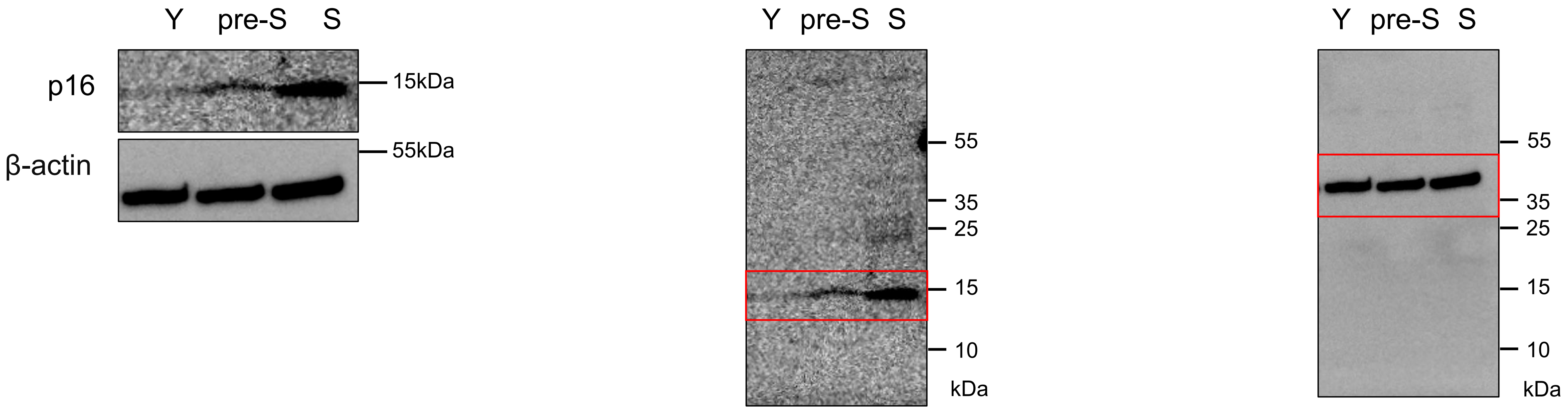

IB: p16 (16kDa) **IB: β-actin (45kDa)**

Figure 3 a, N=3

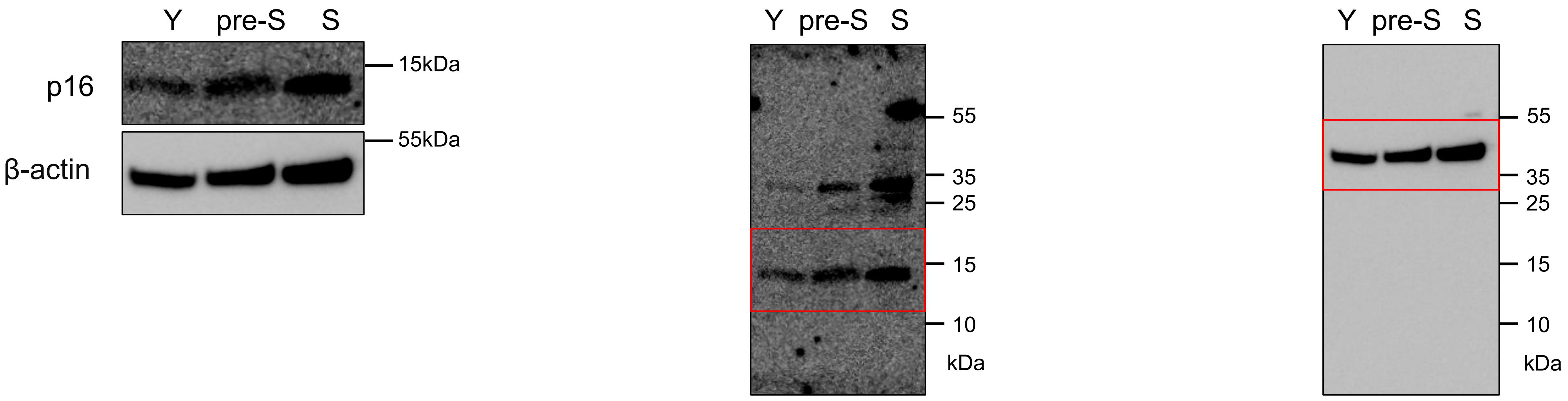

IB: p16 (16kDa) **IB: β-actin (45kDa)**

Figure 3 b, N=1

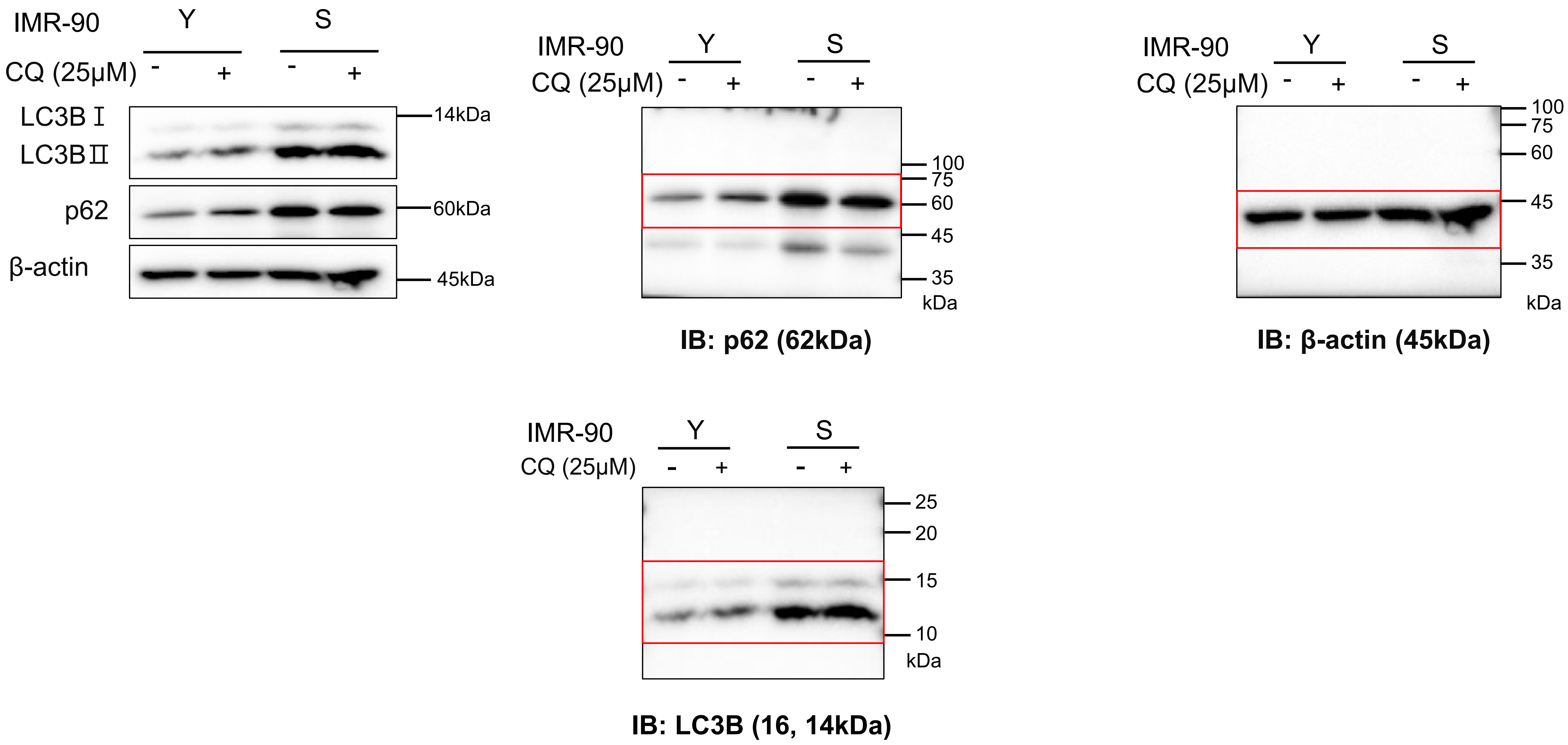

Figure 3 b, N=2

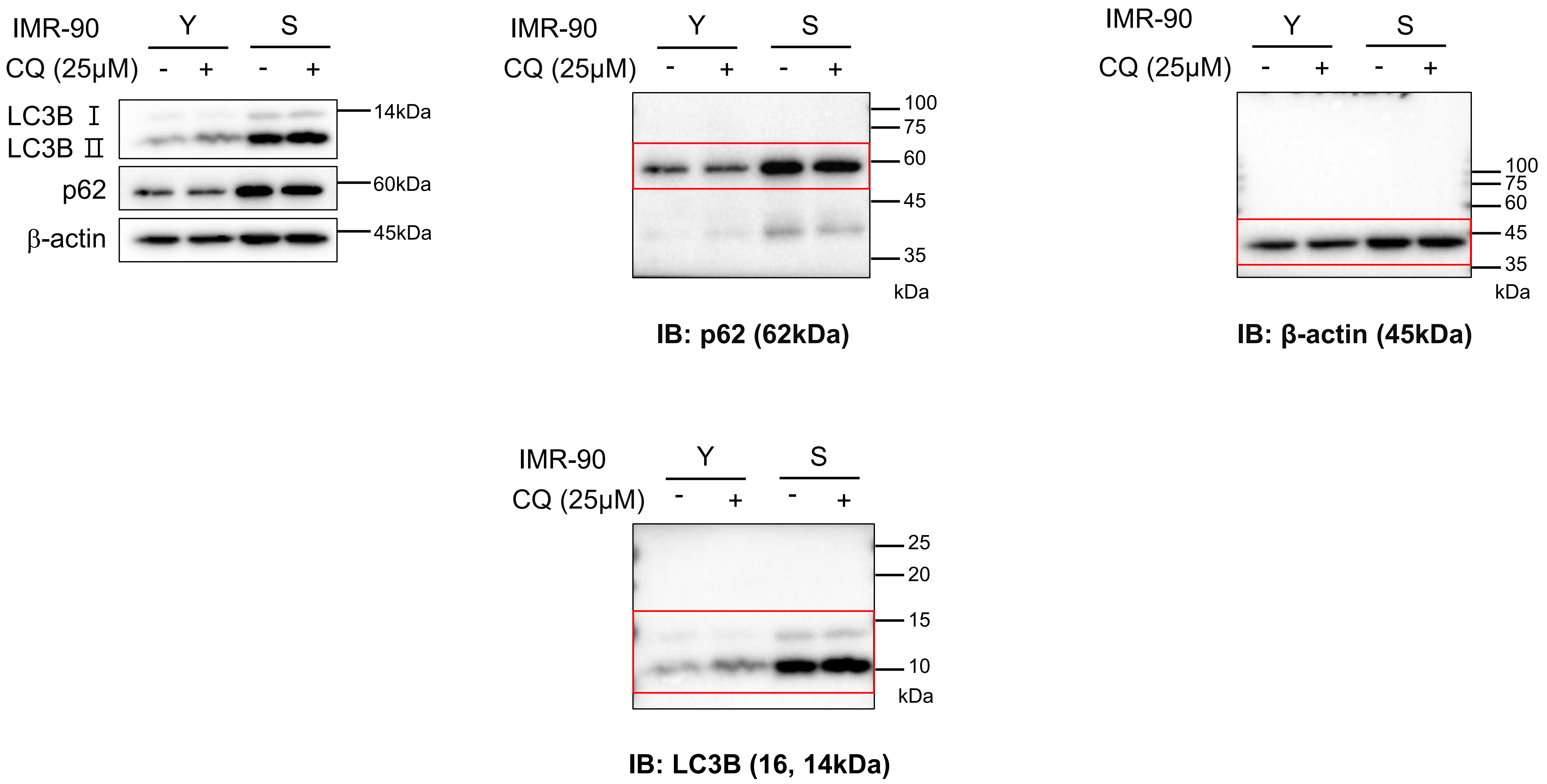

Figure 3 b, N=3

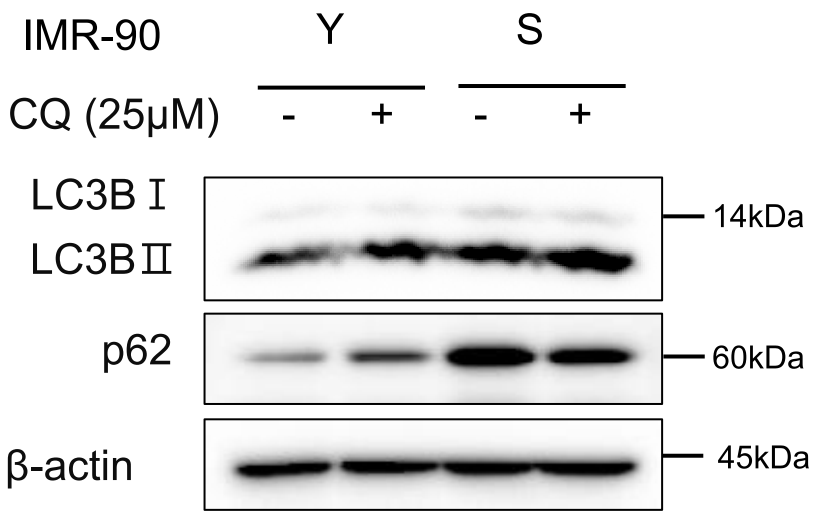

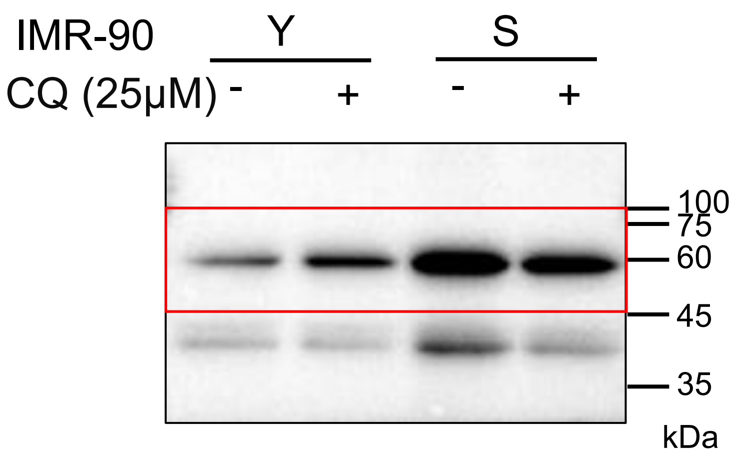

IB: p62 (62kDa)

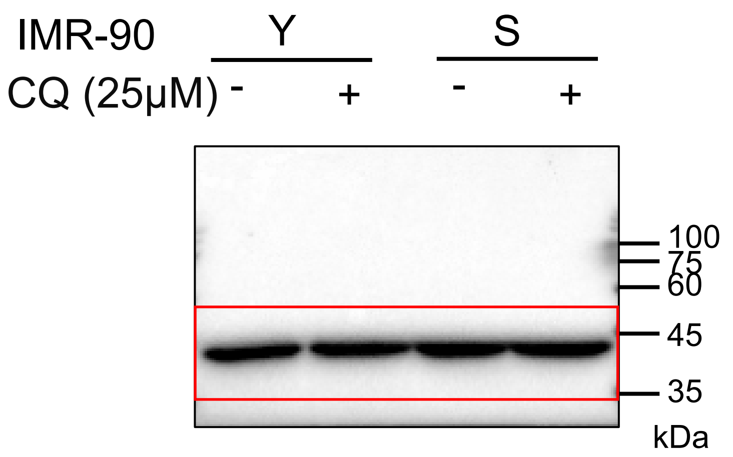

IB: β-actin (45kDa)

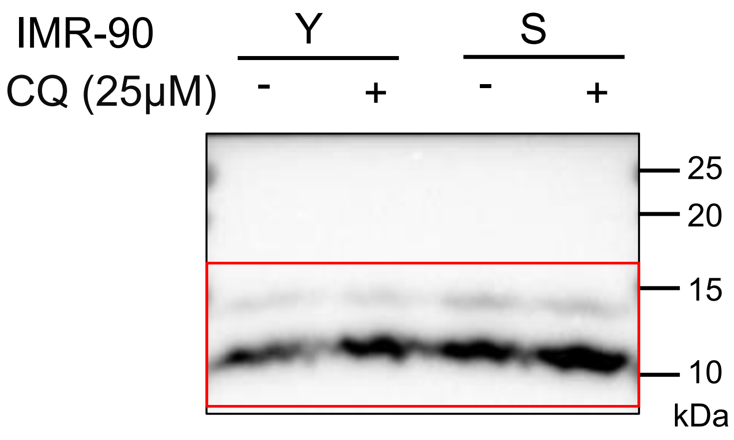

IB: LC3B (16, 14kDa)

Figure 3 b, N=4

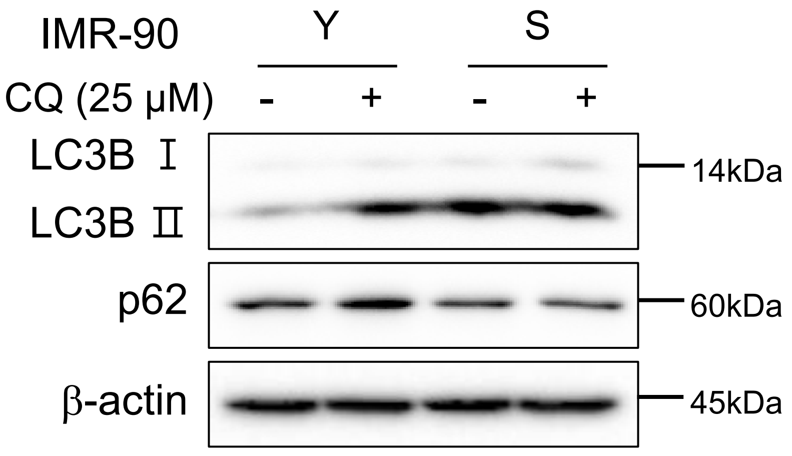

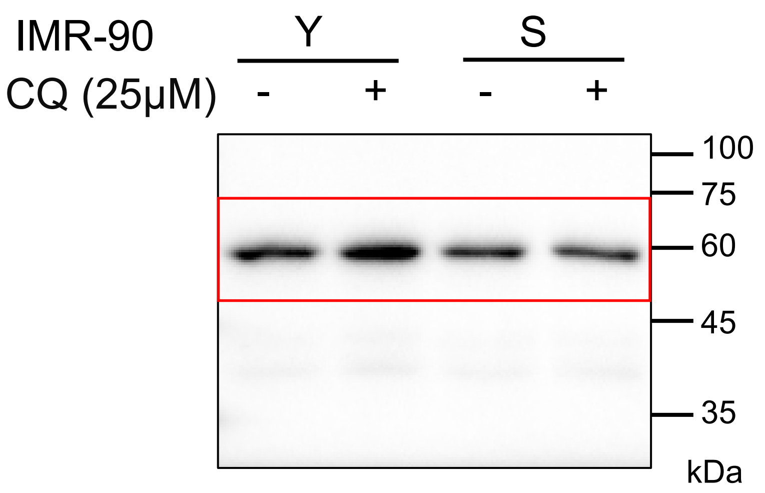

IB: p62 (62kDa)

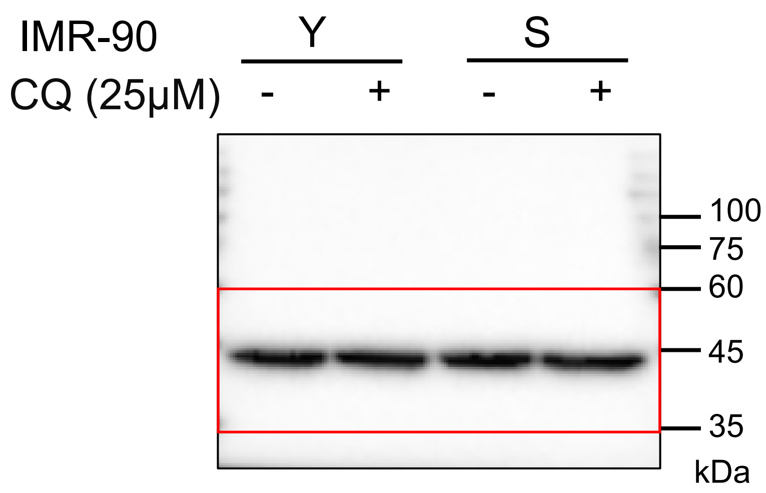

IB: β-actin (45kDa)

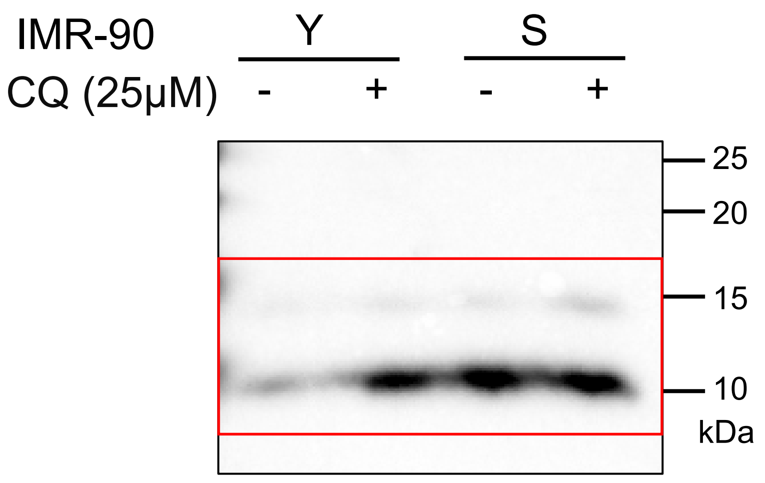

IB: LC3B (16, 14kDa)

Figure 3 c, N=1

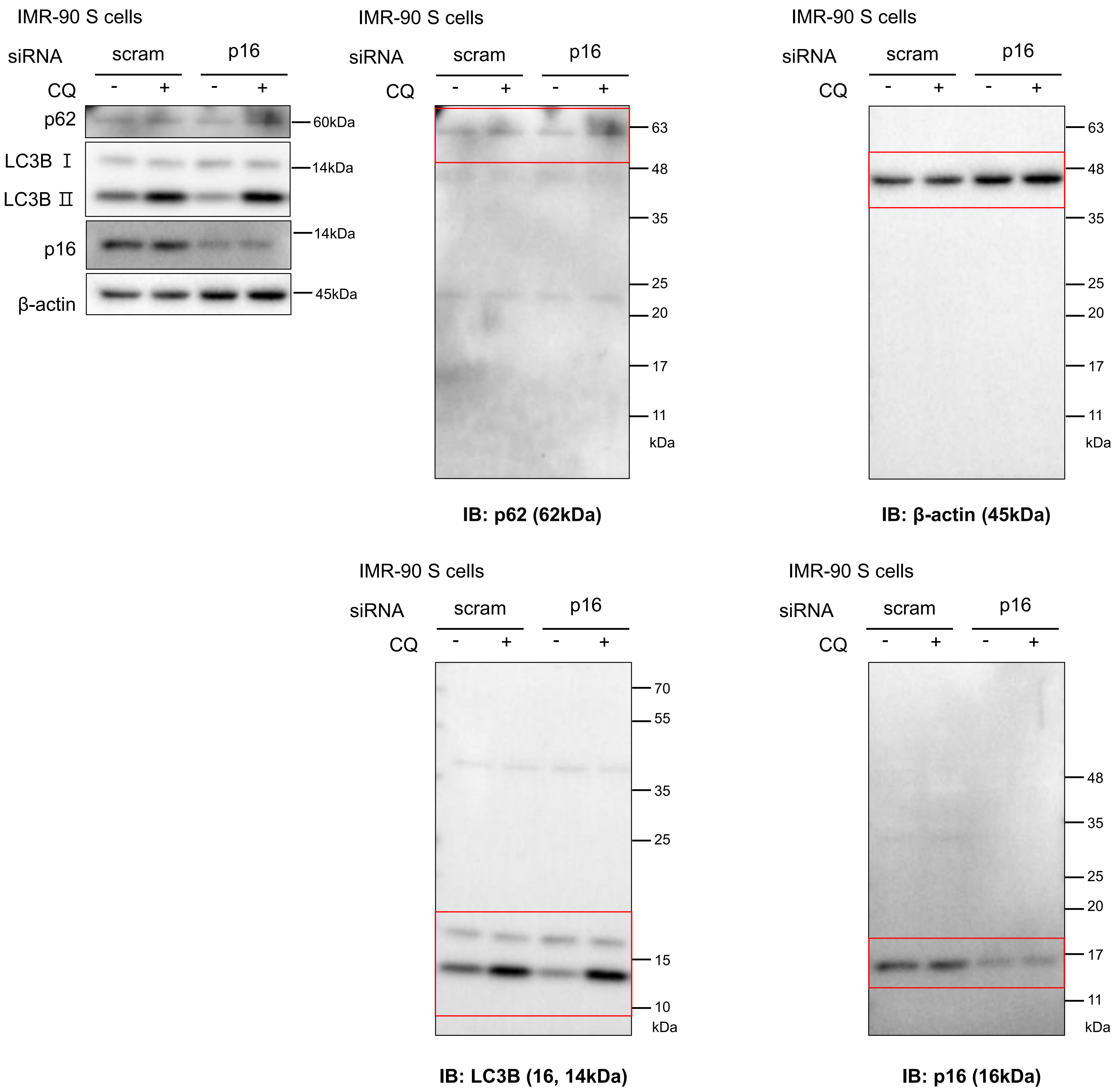
IMR-90 S cells
siRNA scram p16
CQ - + - +
p62
LC3B Ⅰ
LC3B Ⅱ
p16
β-actin
60kDa
14kDa
14kDa
45kDa
63
48
35
25
20
17
11
kDa
IB: p62 (62kDa)
IB: β-actin (45kDa)
70
55
15
10
IB: LC3B (16, 14kDa)
IB: p16 (16kDa)

Figure 3 c, N=2

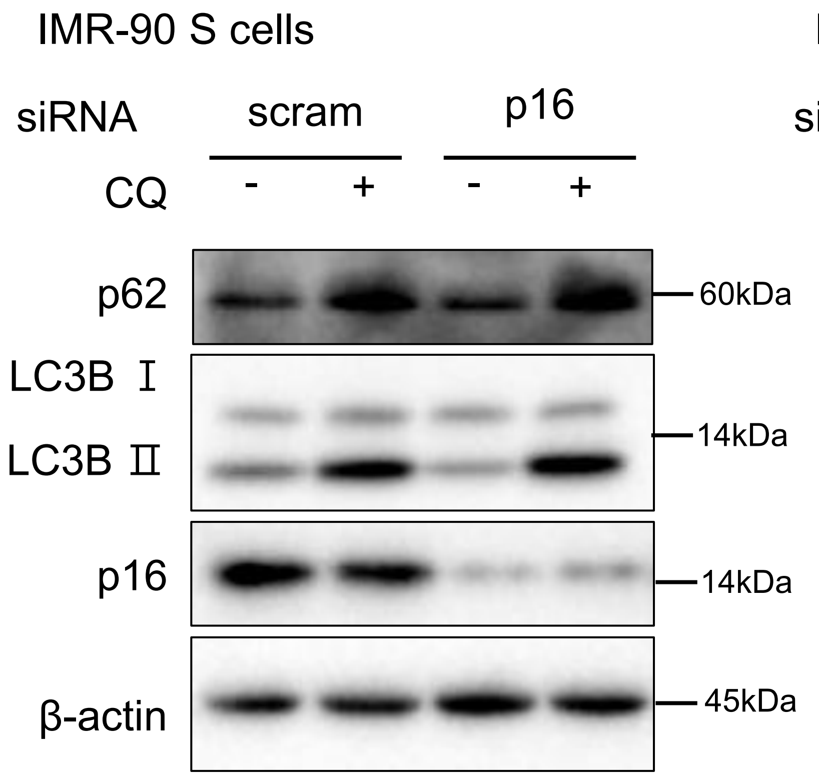

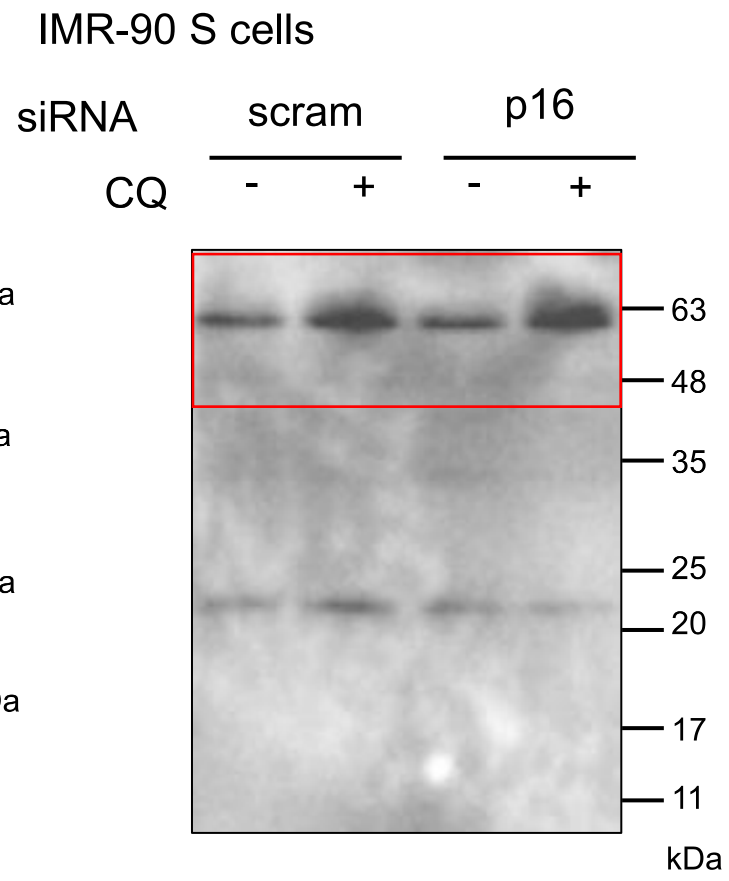

IB: p62 (62kDa)

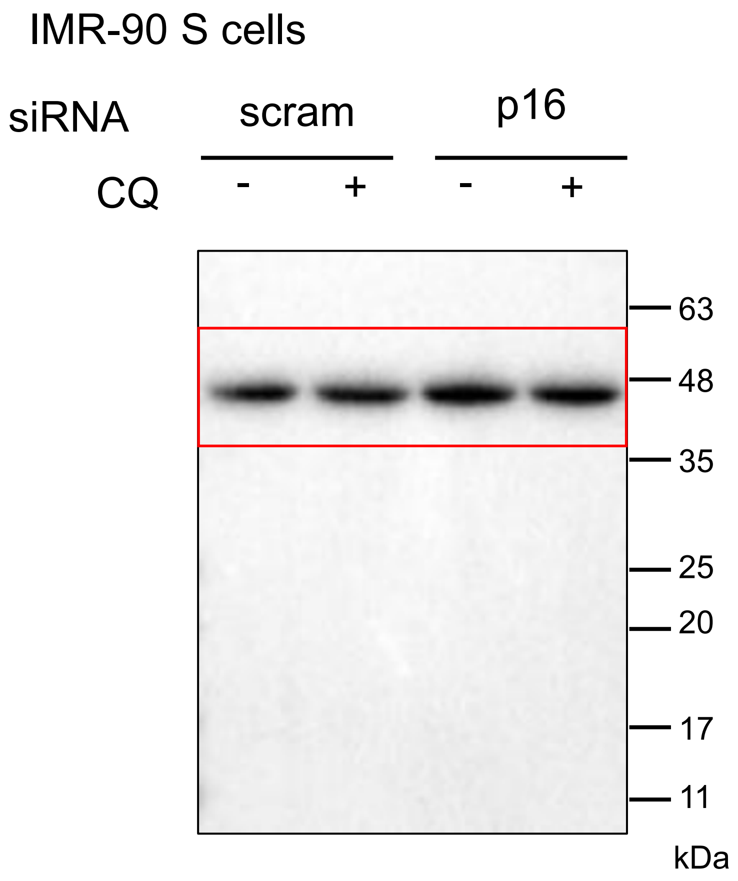

IB: β-actin (45kDa)

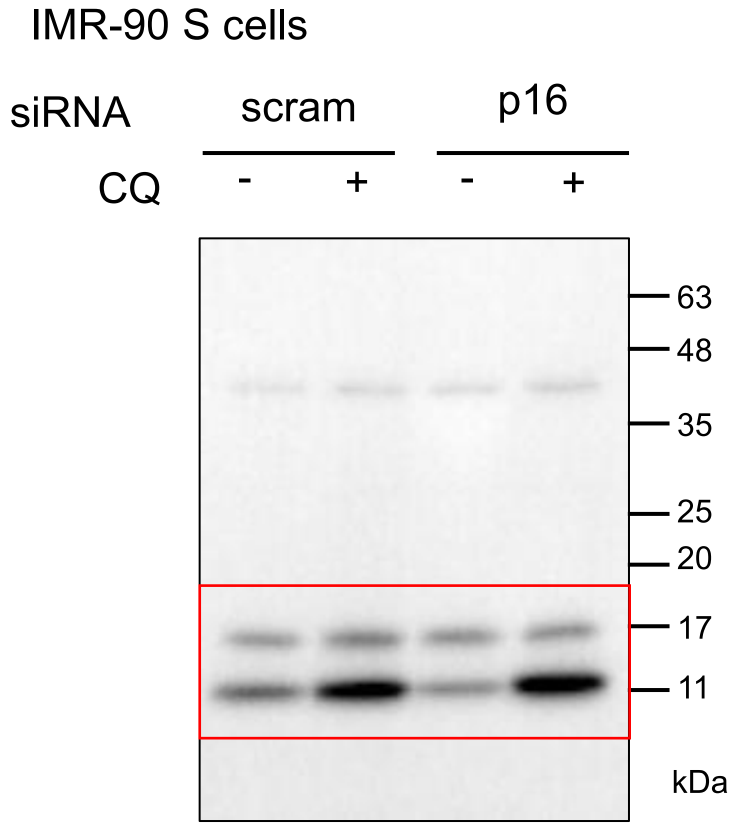

IB: LC3B (16, 14kDa)

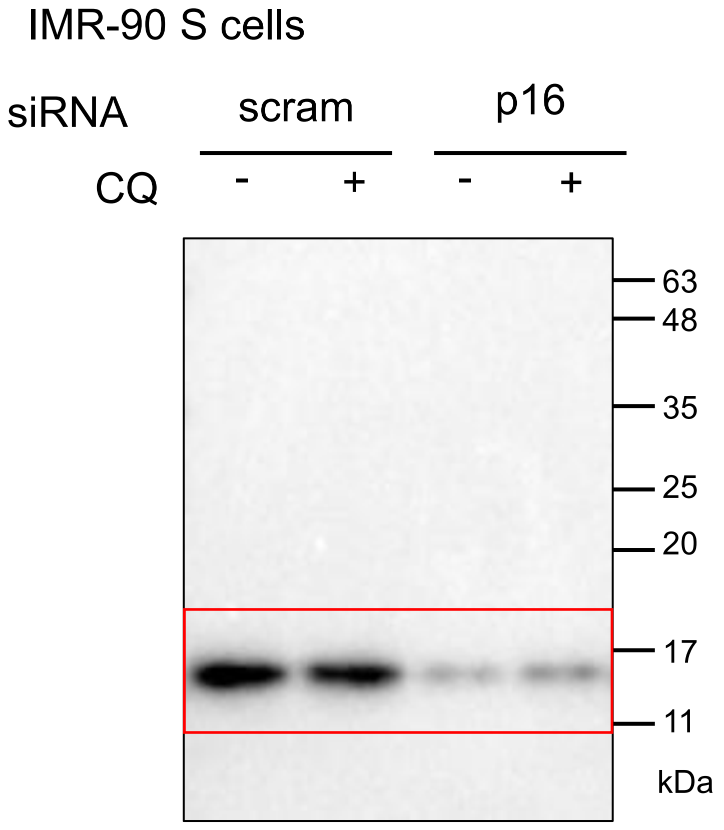

IB: p16 (16kDa)

Figure 3 c, N=3

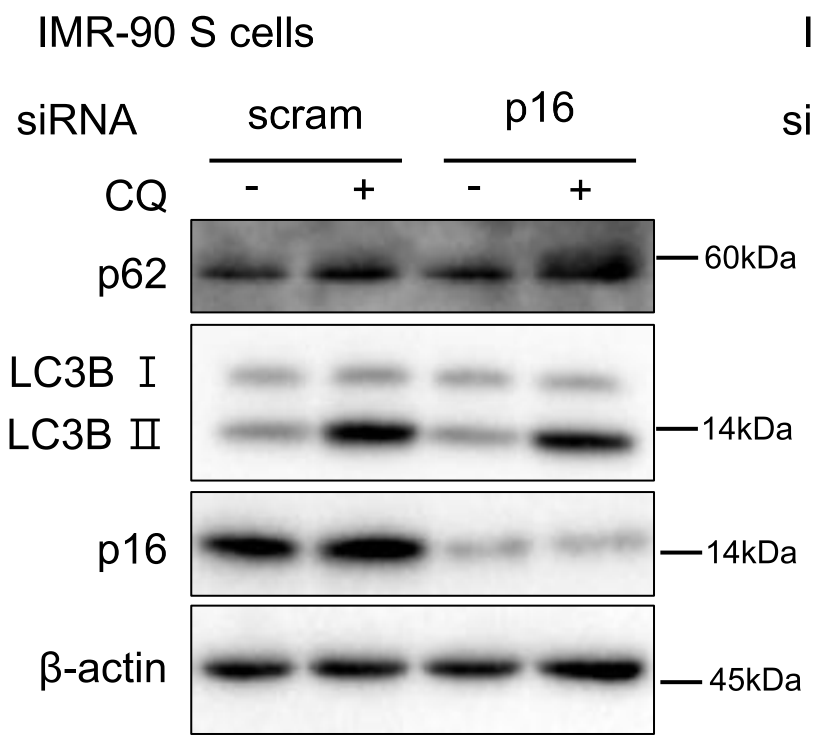

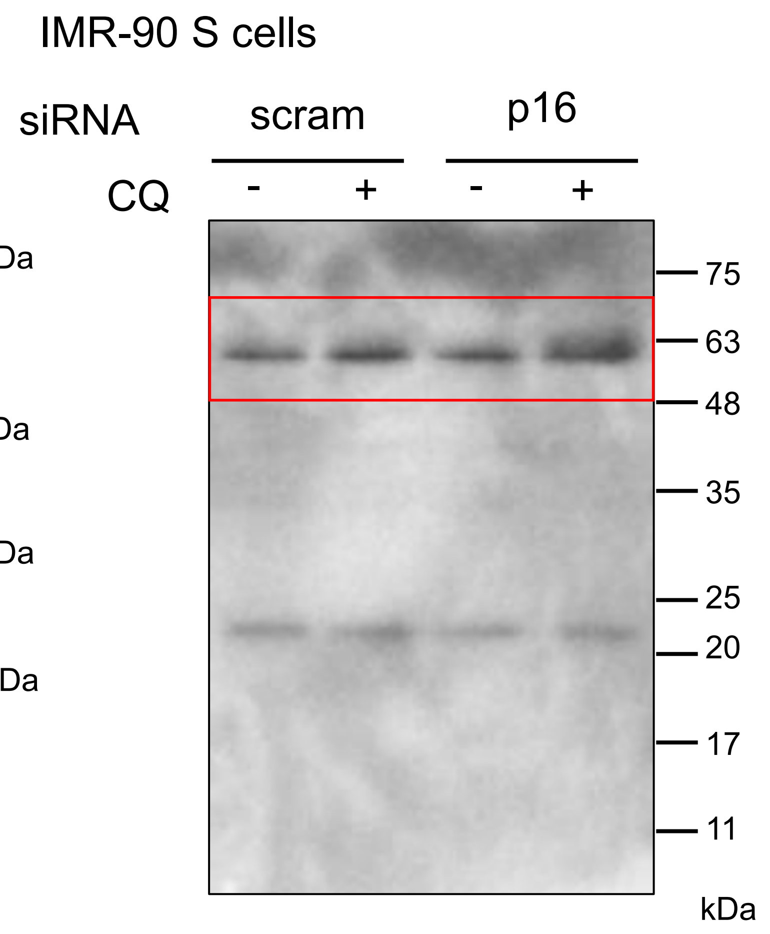

IB: p62 (62kDa)

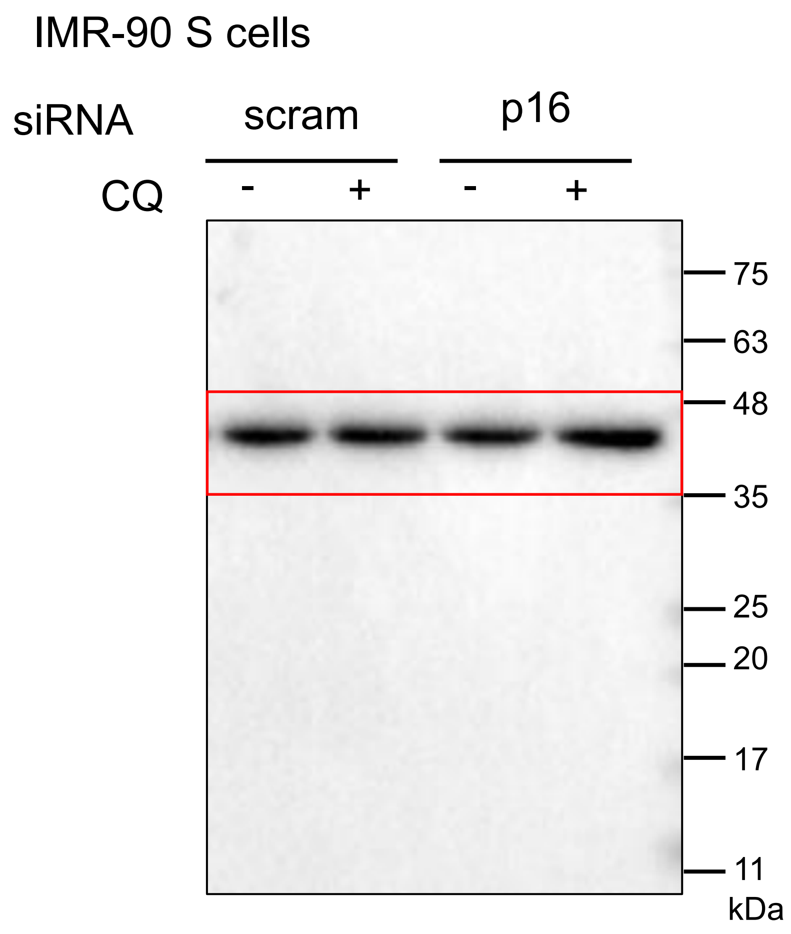

IB: β-actin (45kDa)

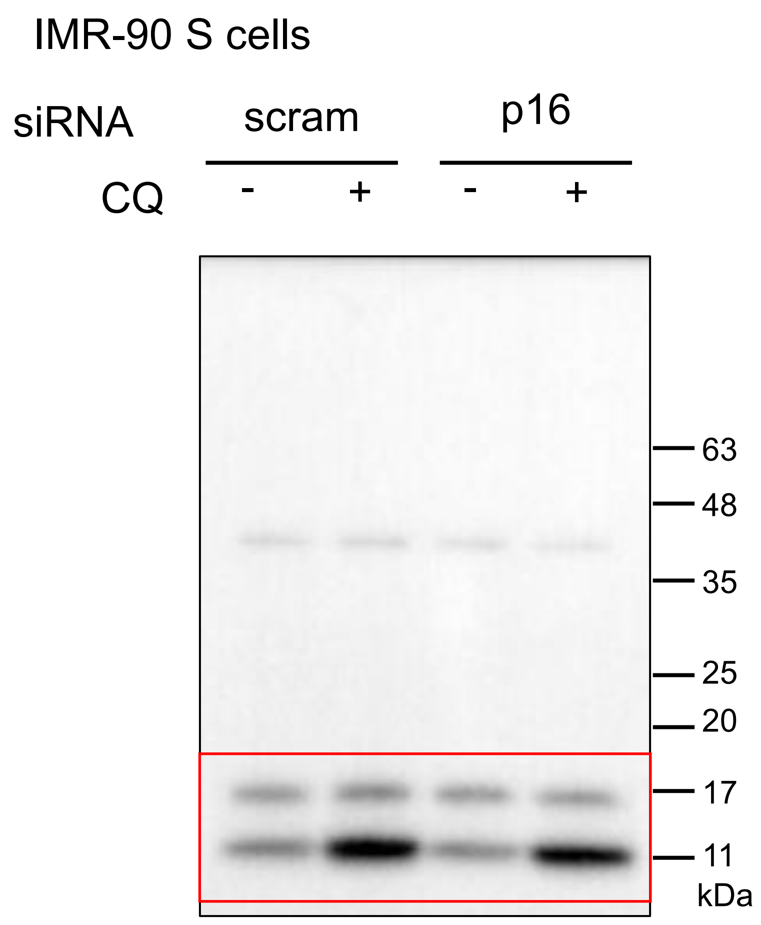

IB: LC3B (16, 14kDa)

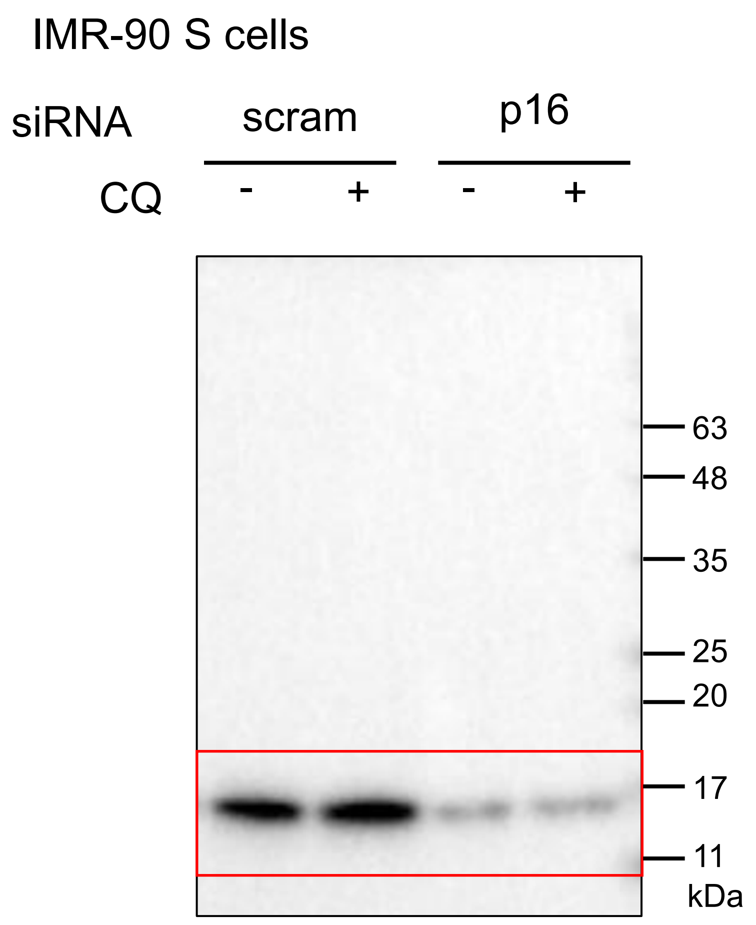

IB: p16 (16kDa)

Figure 4 d, N=1

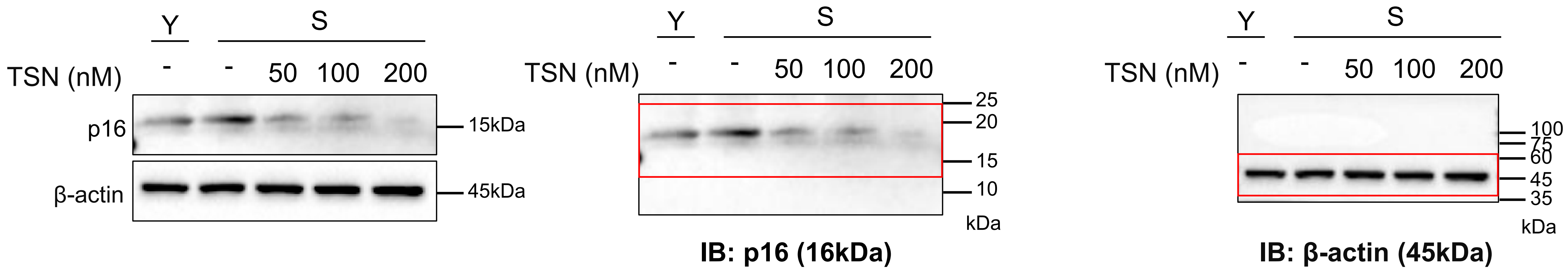

Figure 4 d, N=2

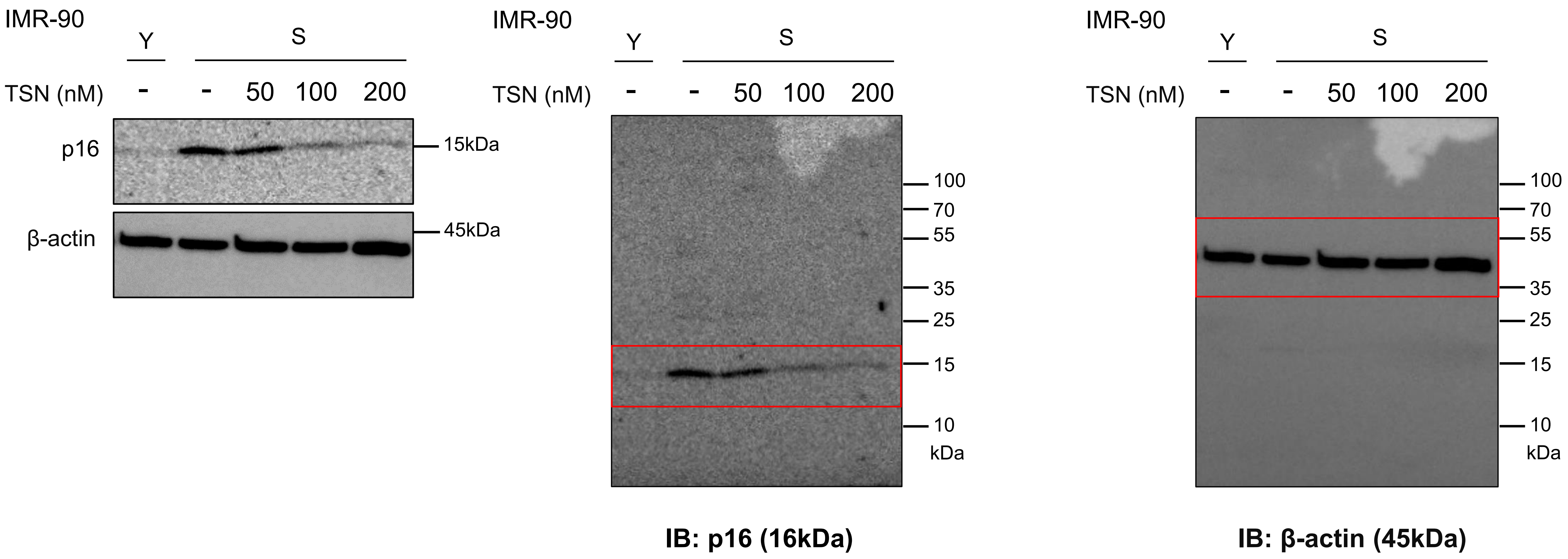

Figure 4 d, N=3

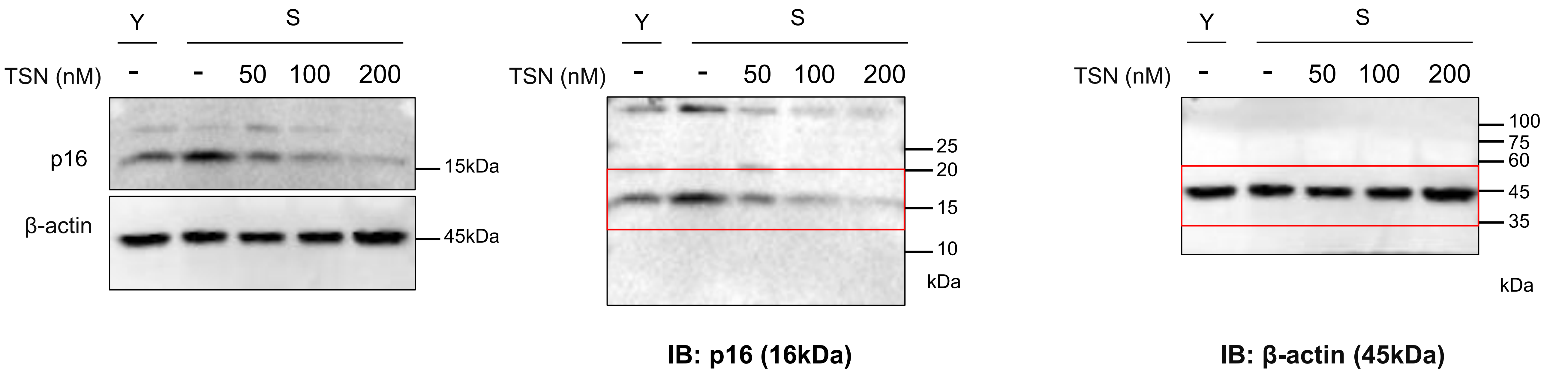

Figure 4 d, N=4

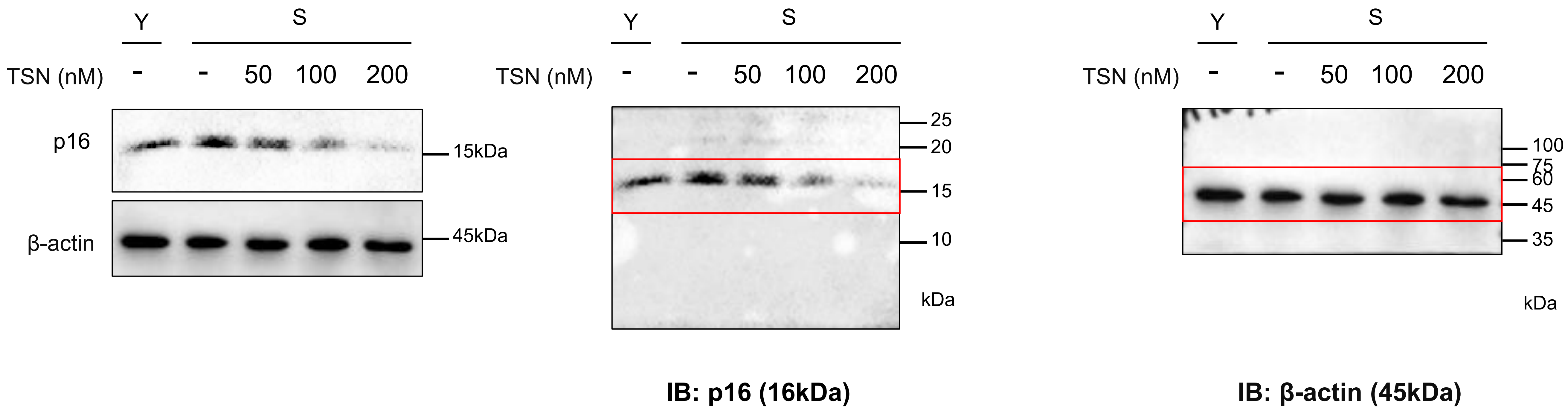

Figure 4 g, N=1

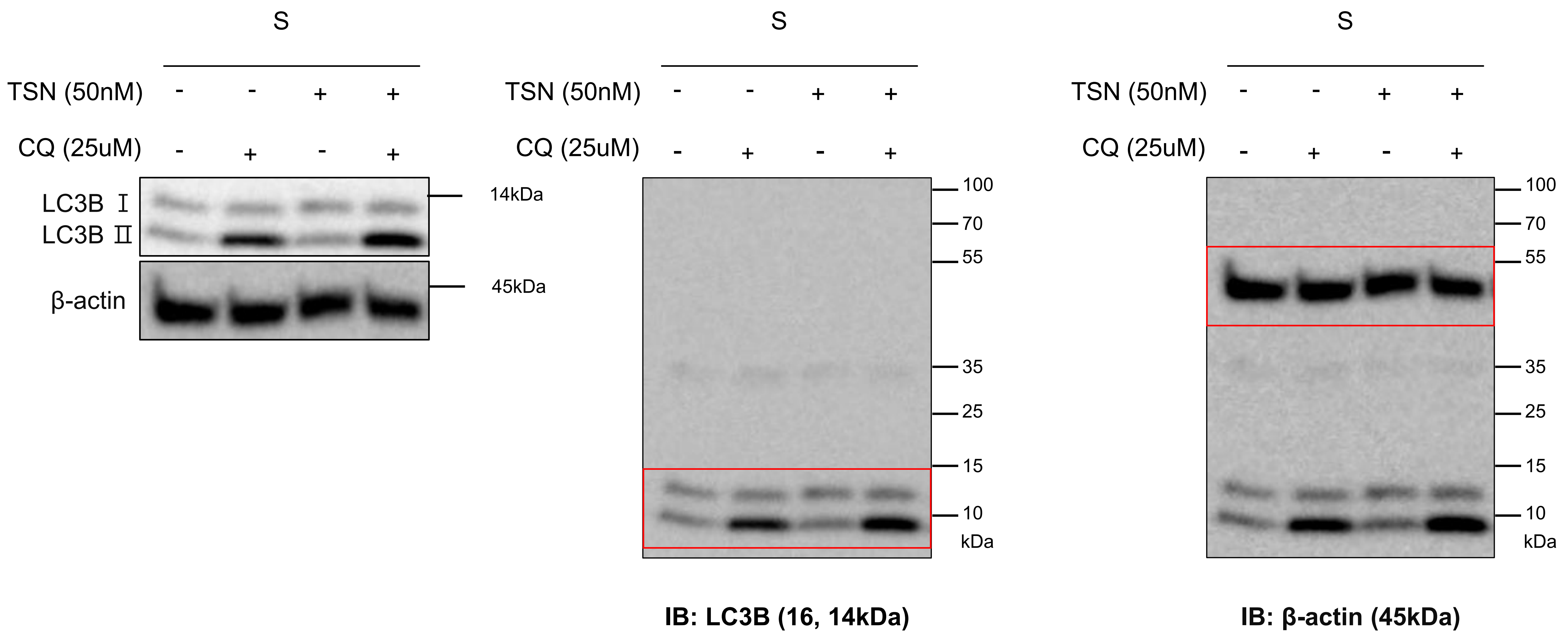

Figure 4 g, N=2

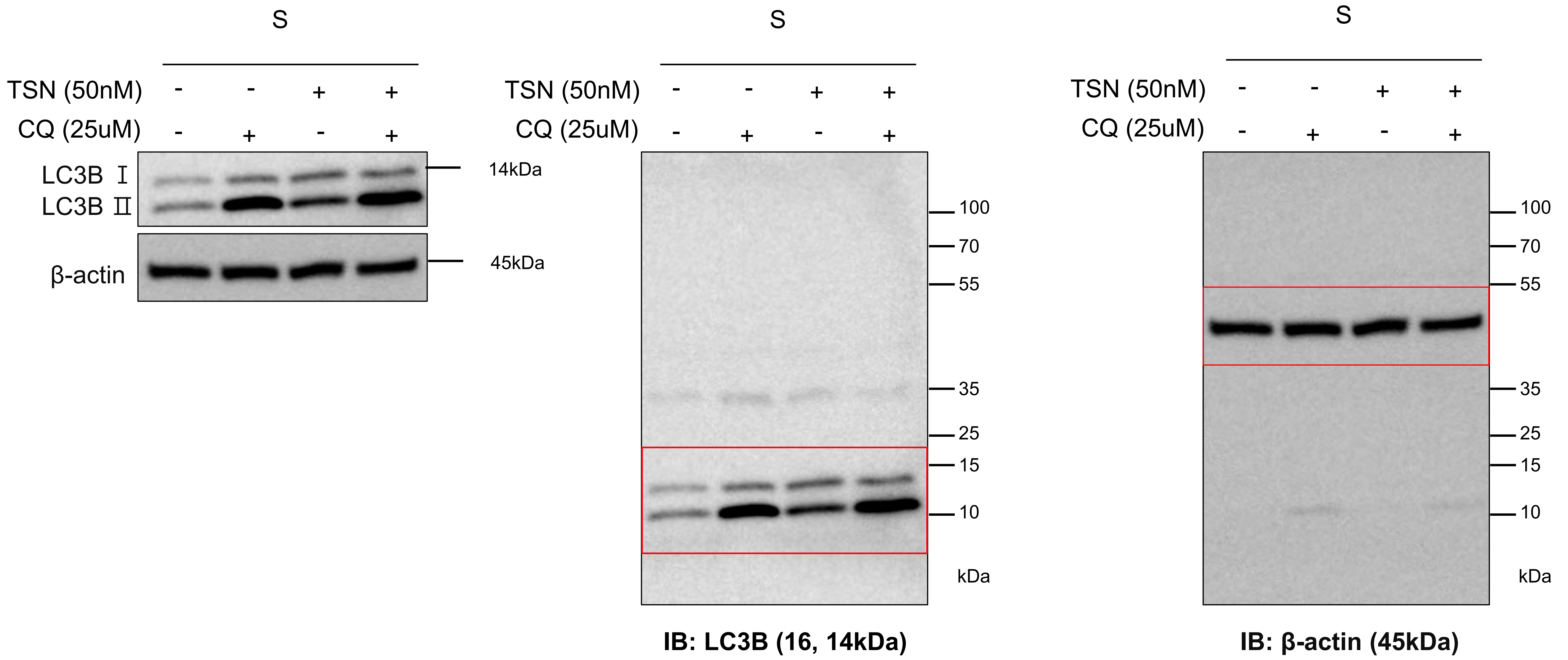

Figure 4 g, N=3

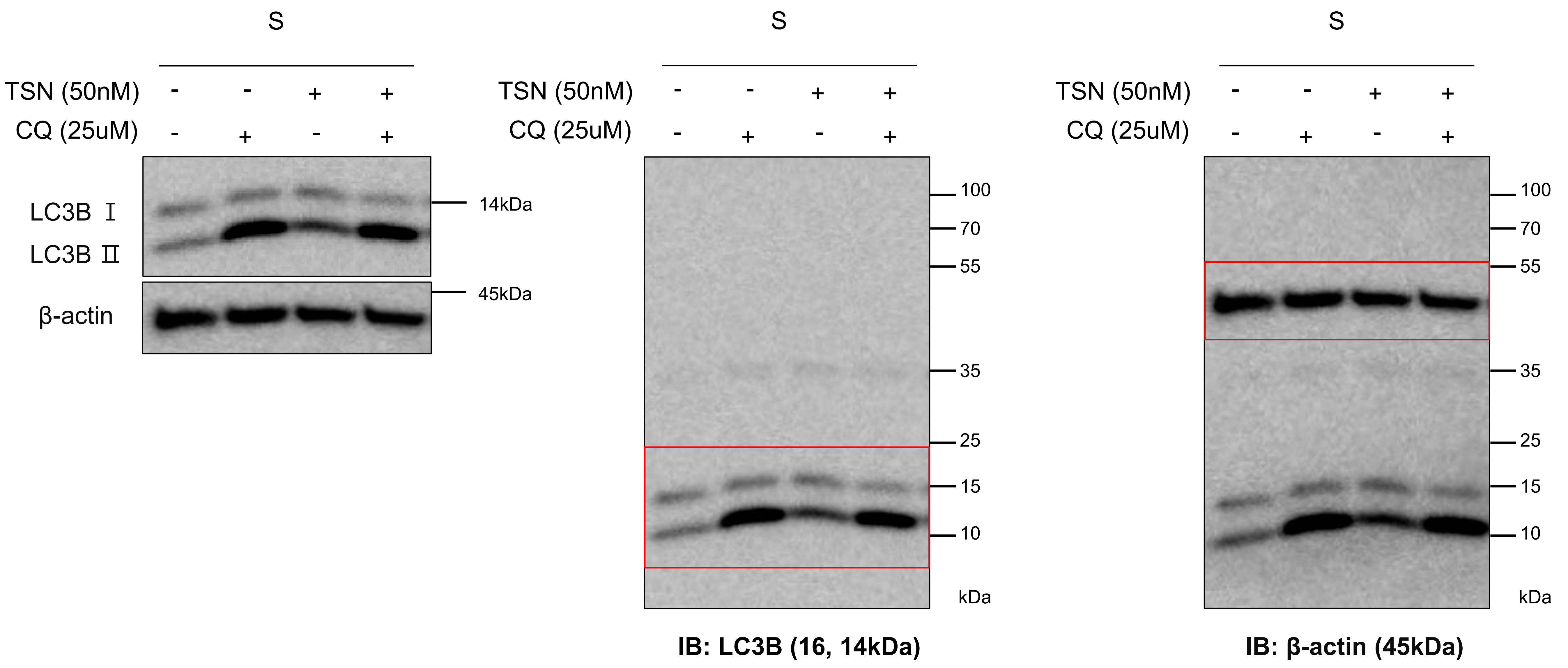

Figure 4 h, N=1

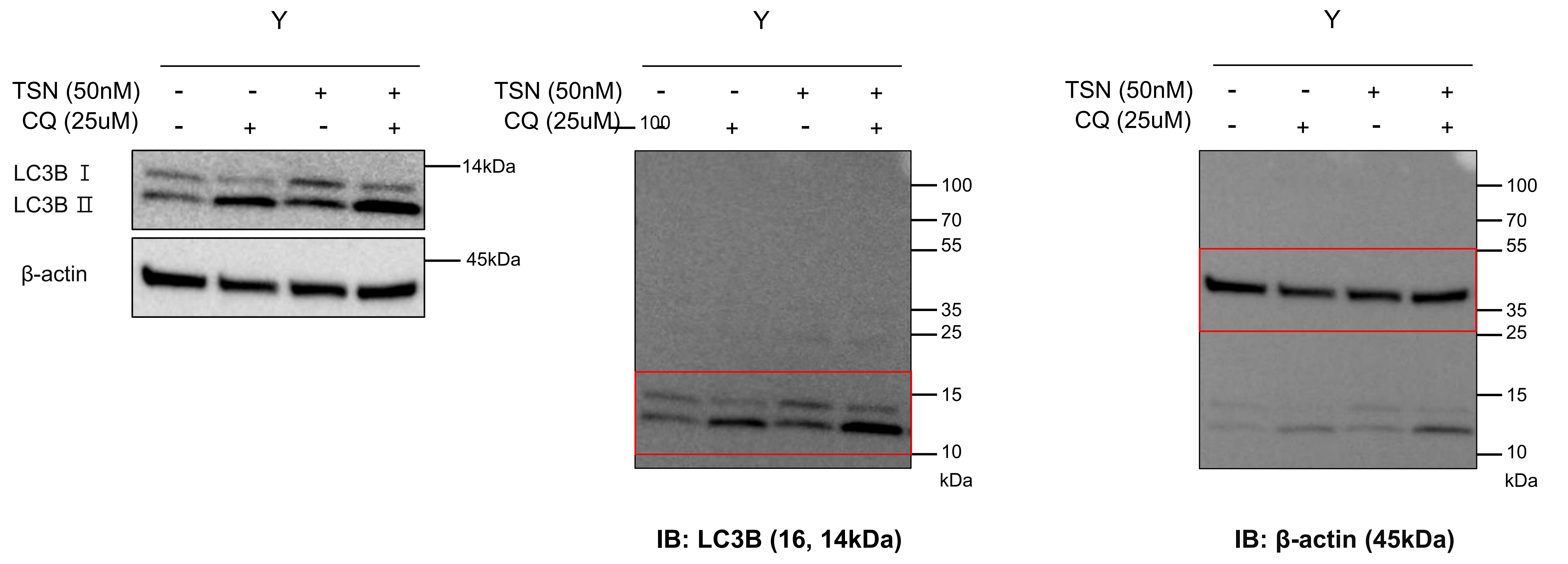

Figure 4 h, N=2

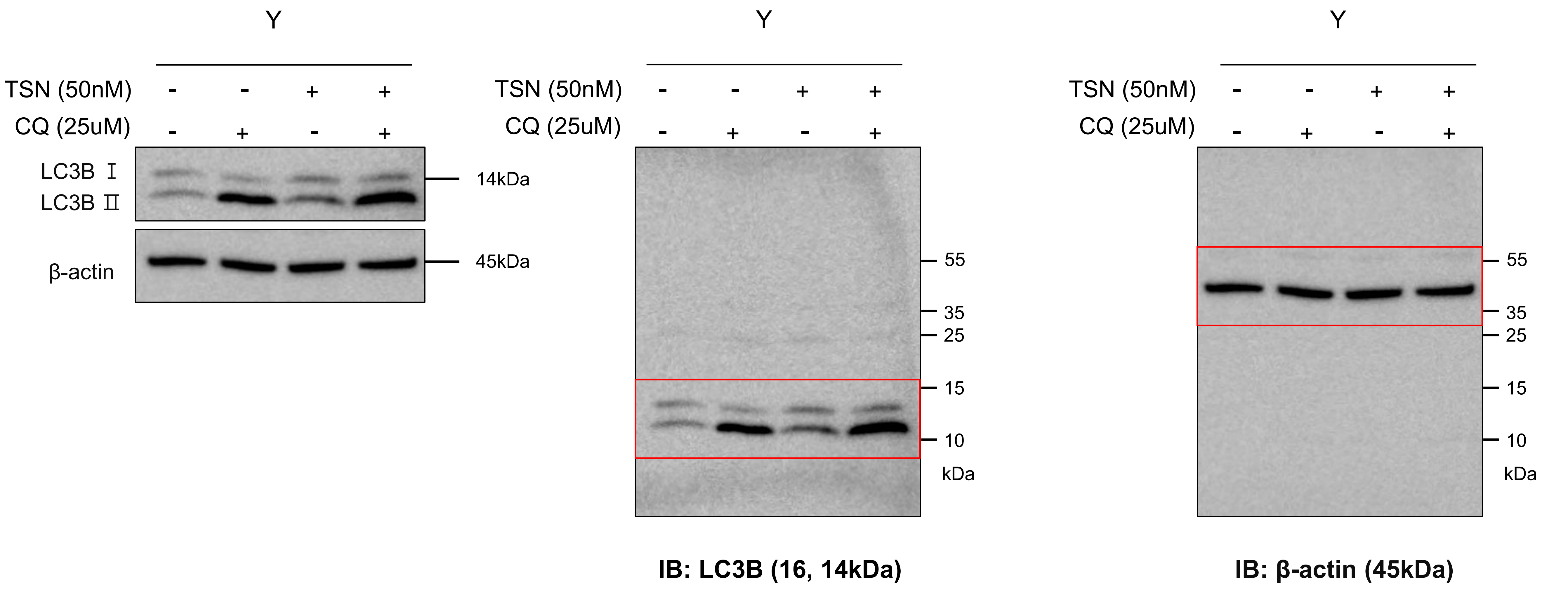

Figure 4 h, N=3

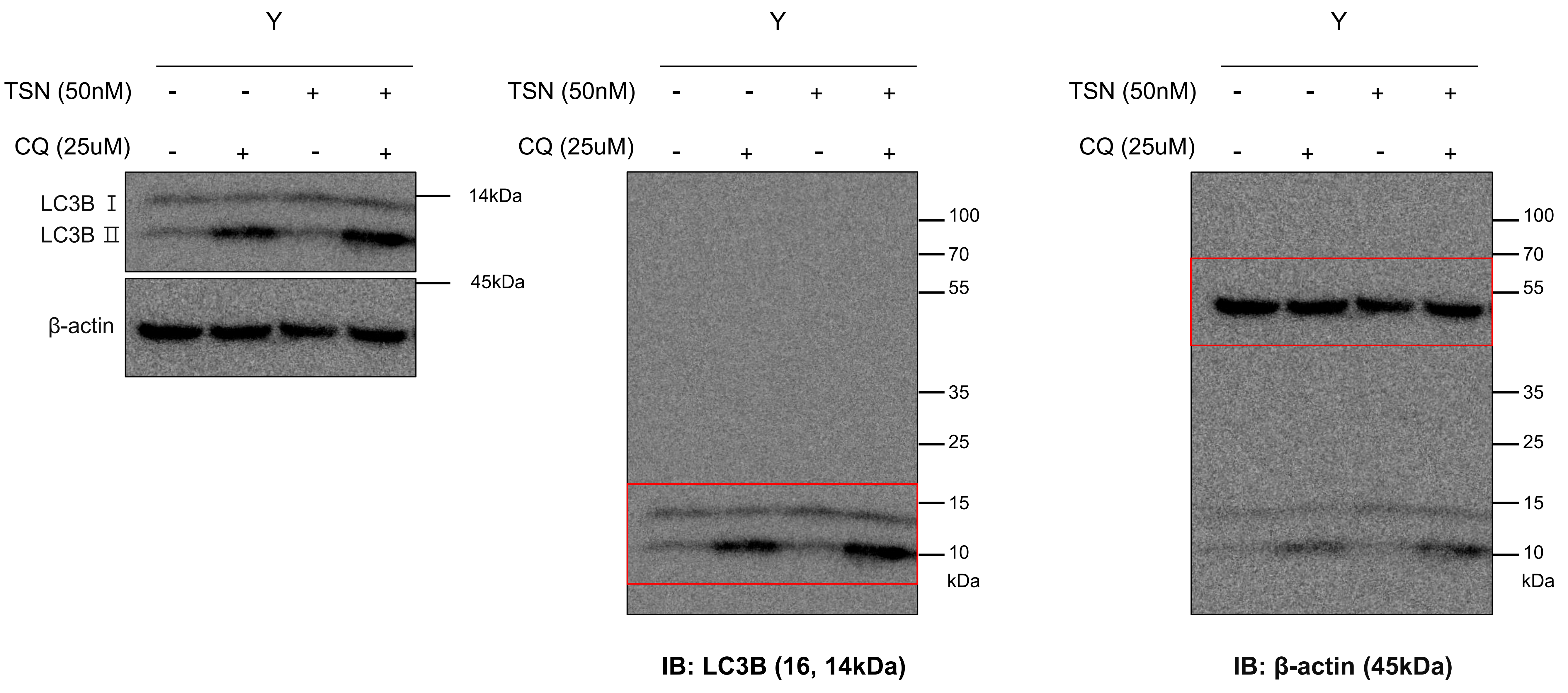

Supplementary Figure 2 b, N=1

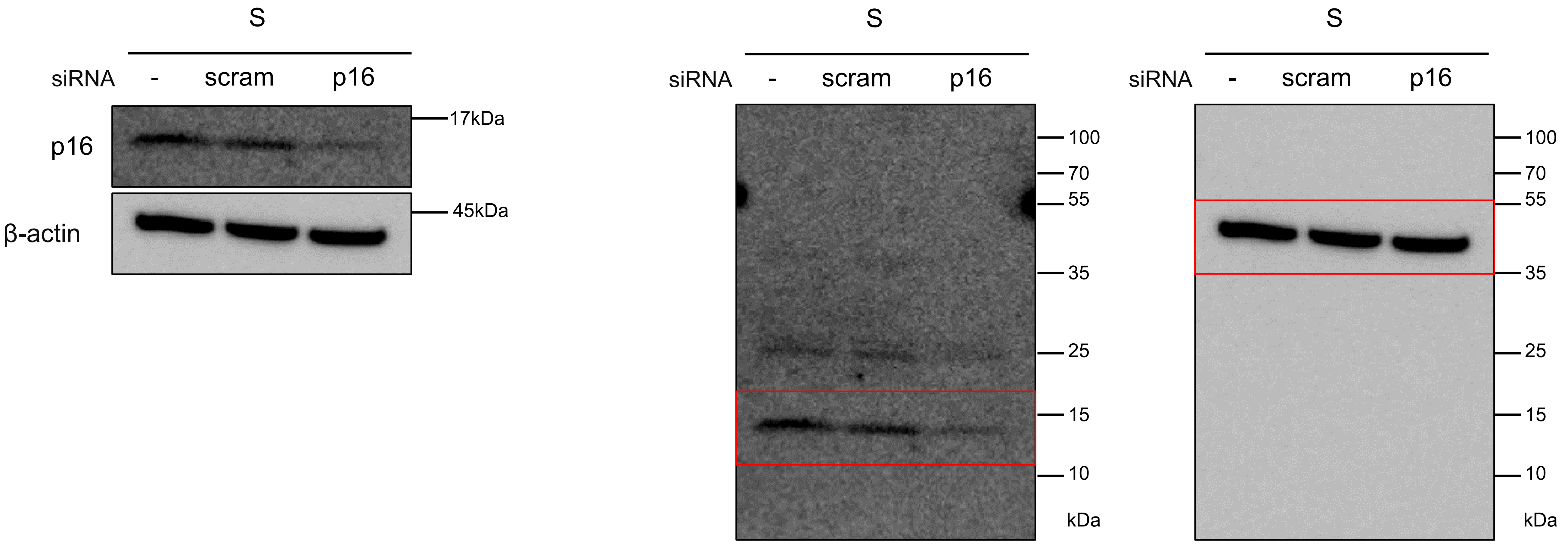

IB: p16 (16kDa) **IB: β-actin (45kDa)**

Supplementary Figure 2 b, N=2

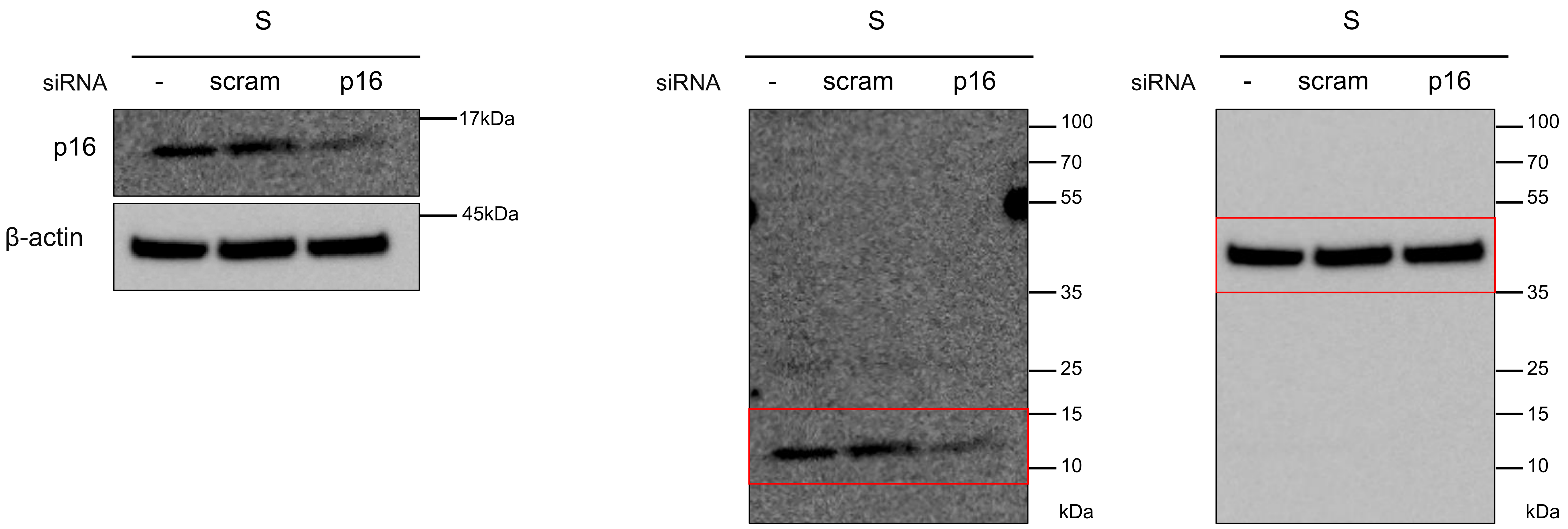

IB: p16 (16kDa) **IB: β-actin (45kDa)**

Supplementary Figure 2 b, N=3

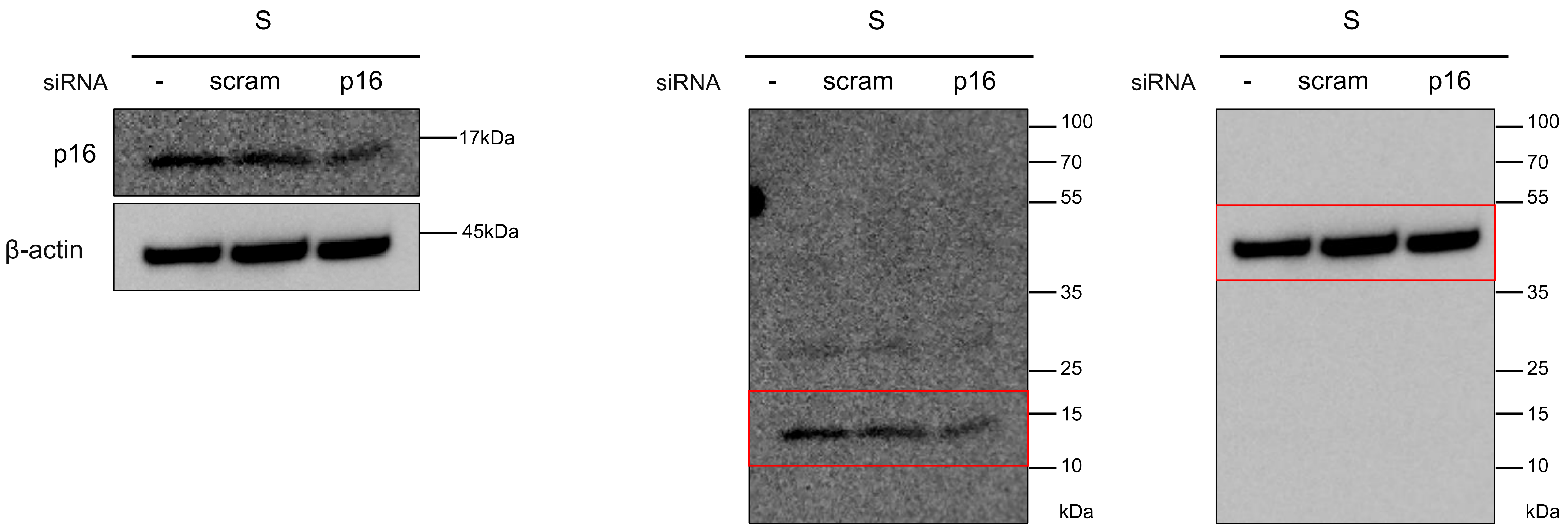

IB: p16 (16kDa) **IB: β-actin (45kDa)**

Supplementary Figure 2 f , N=1, 2

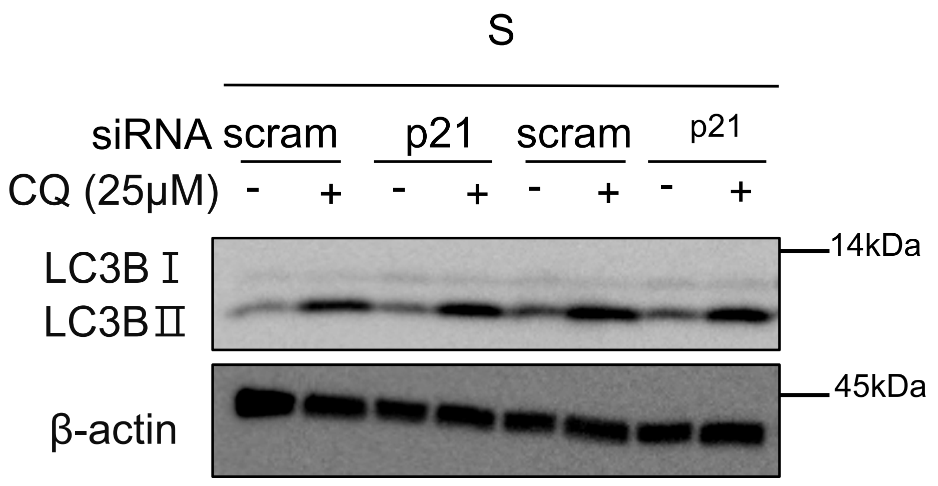

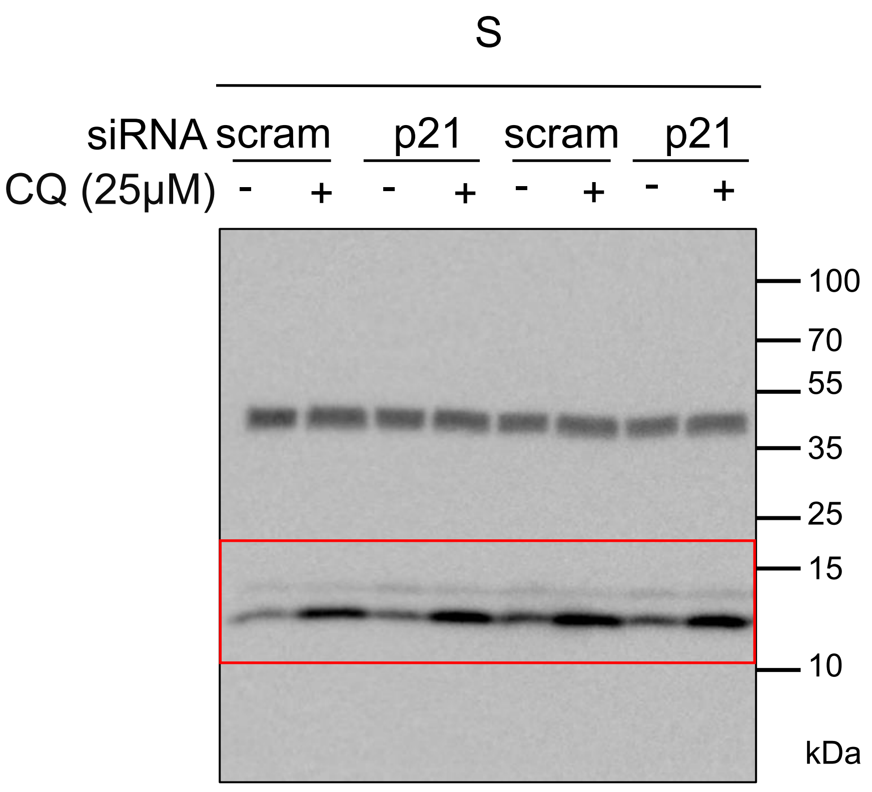

IB: LC3B (16, 14kDa)

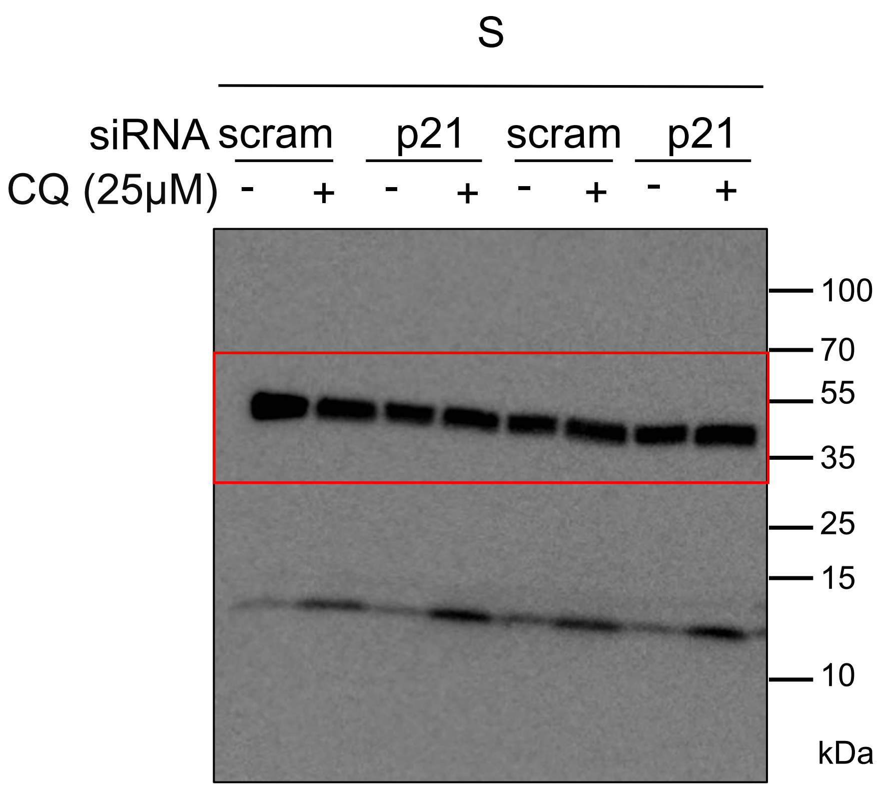

IB: β-actin (45kDa)

Supplementary Figure 2 f, N=3

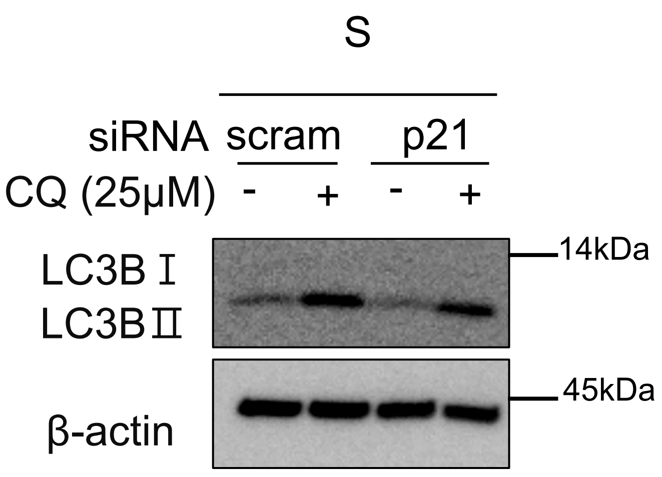

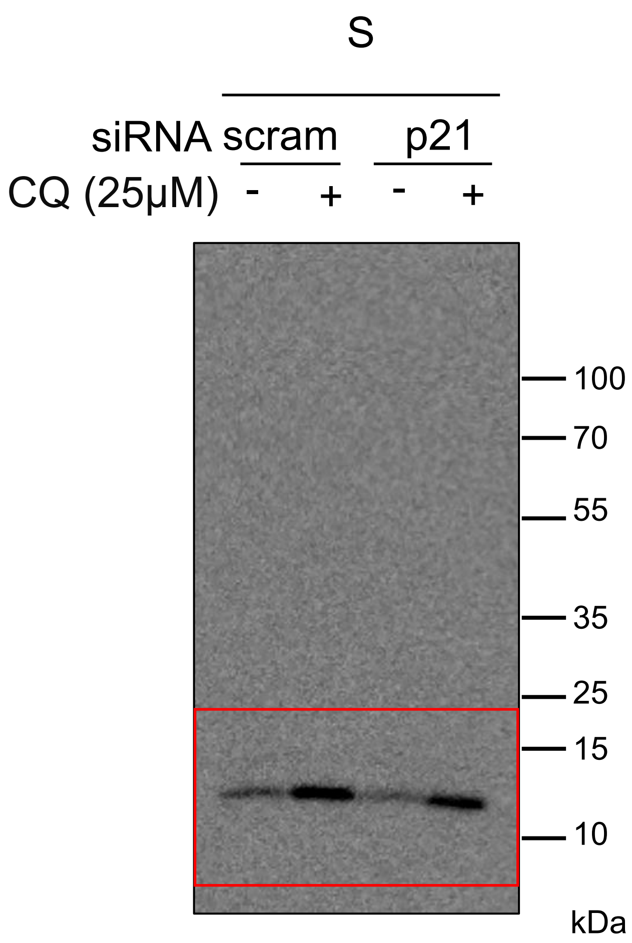

IB: LC3B (16, 14kDa)

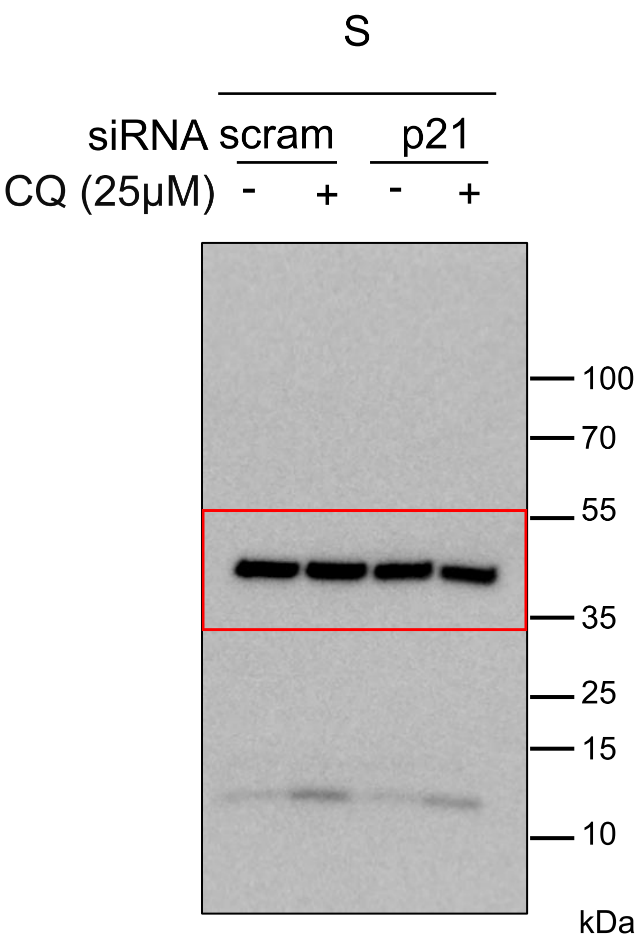

IB: β-actin (45kDa)

Supplementary Figure 2 f , N=1, 2, 3

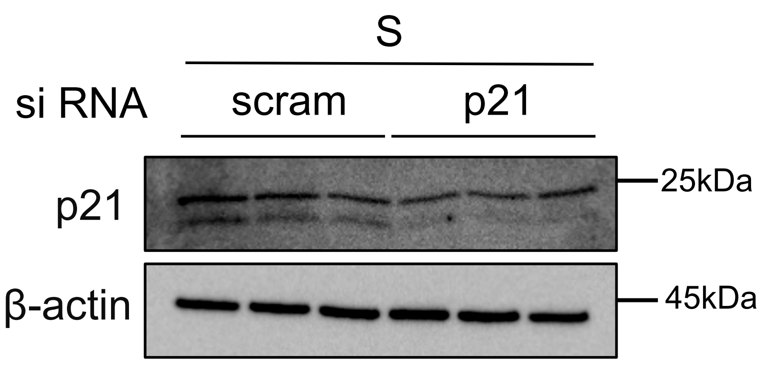

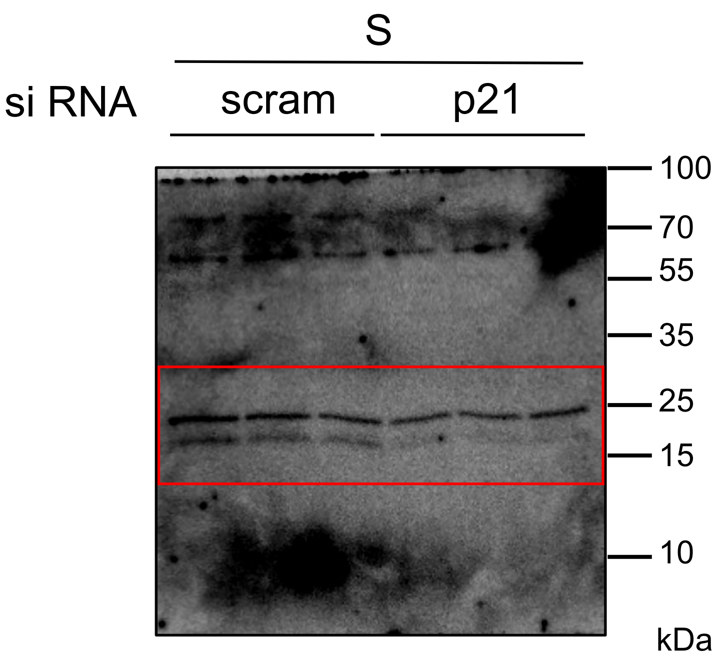

IB: p21 (21kDa)

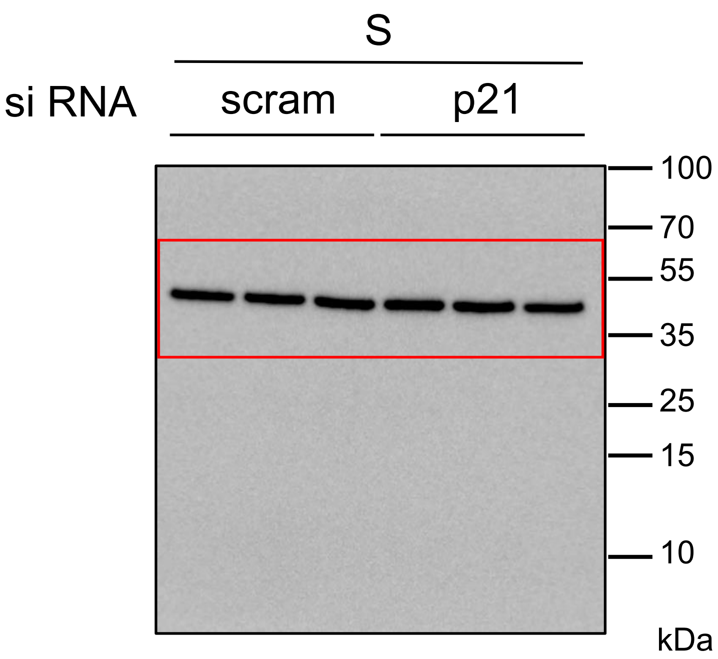

IB: β-actin (45kDa)

Supplementary Figure 2 g, N=1

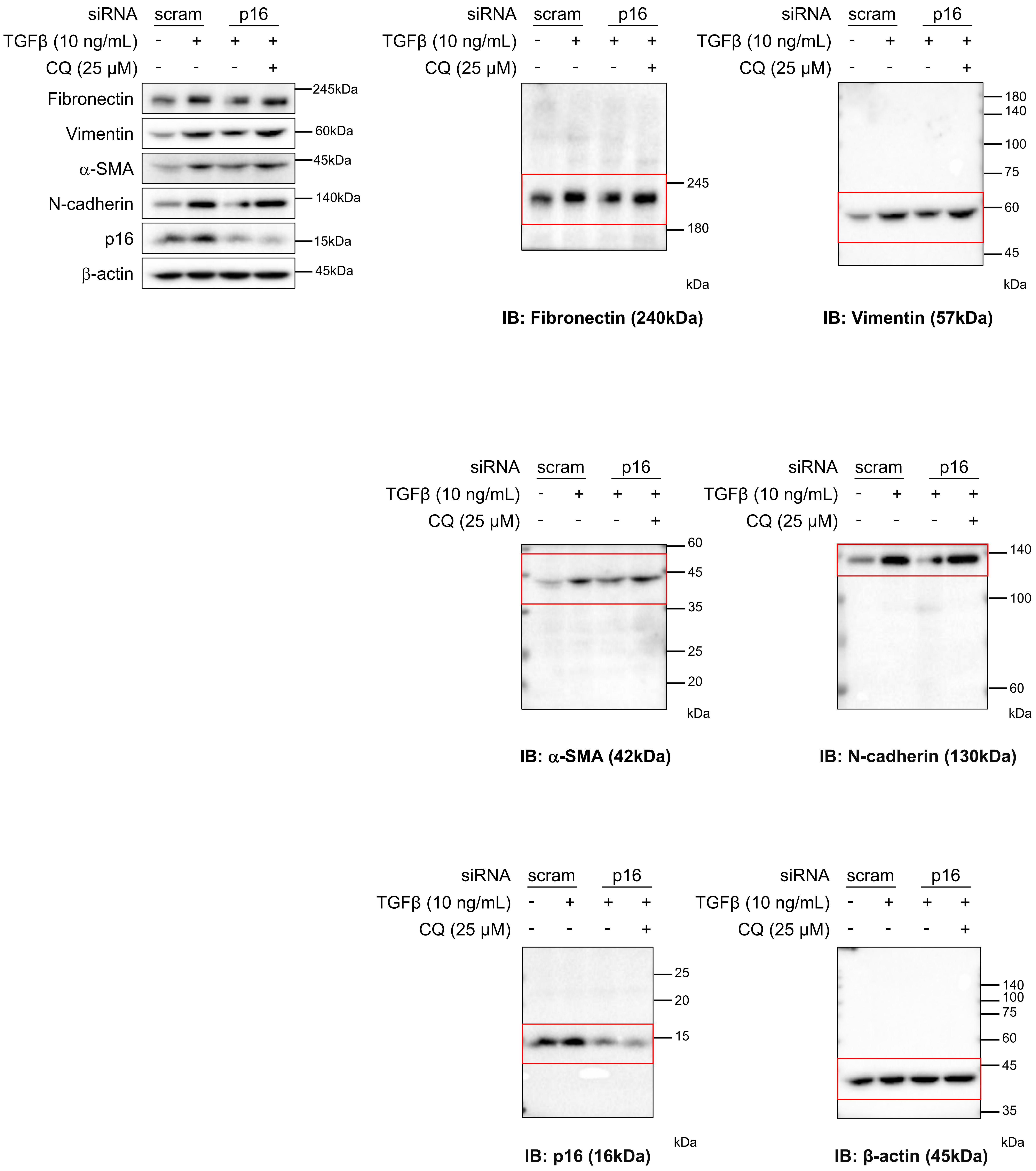

Supplementary Figure 2 g, N=2

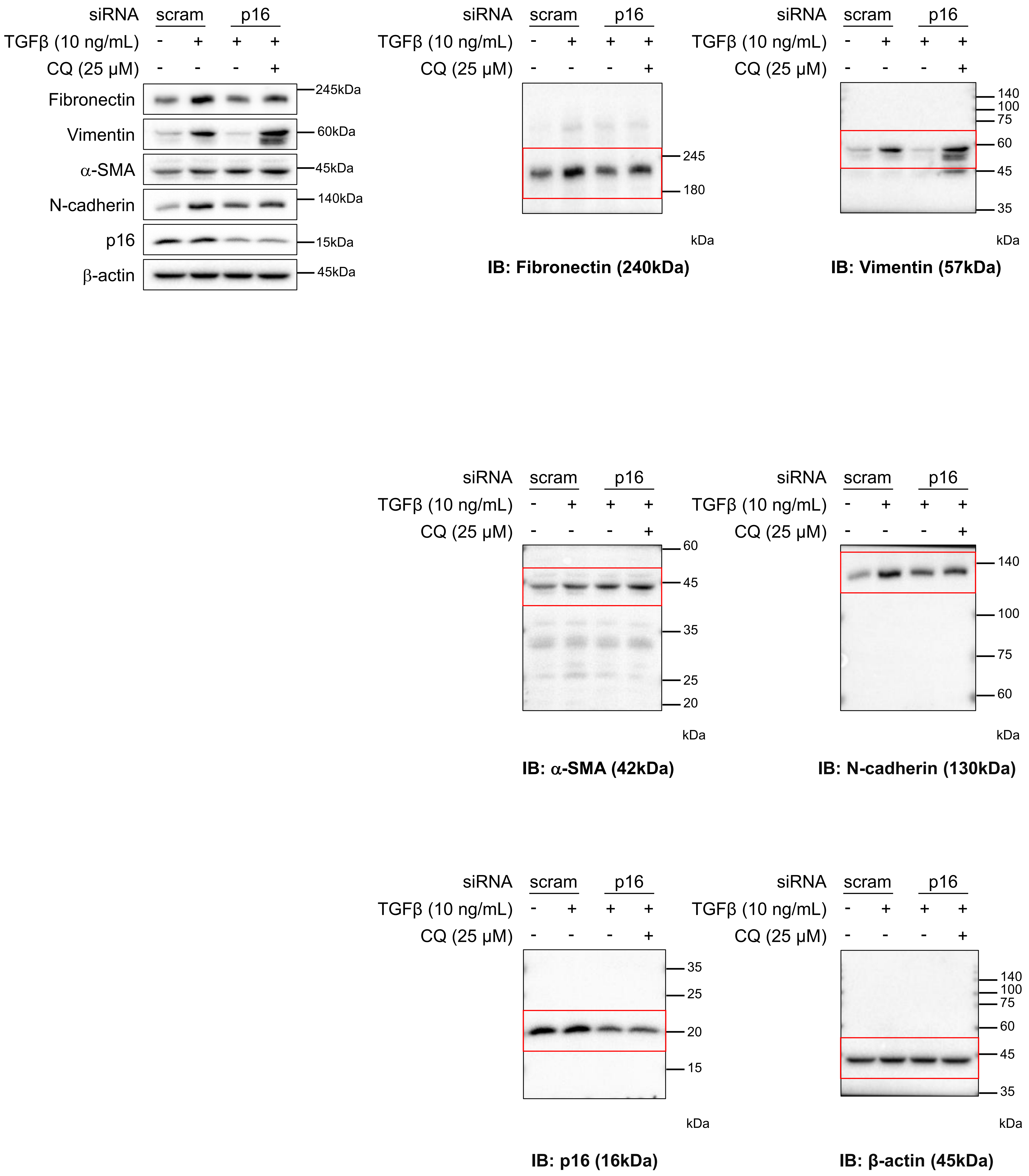

Supplementary Figure 2 g, N=3

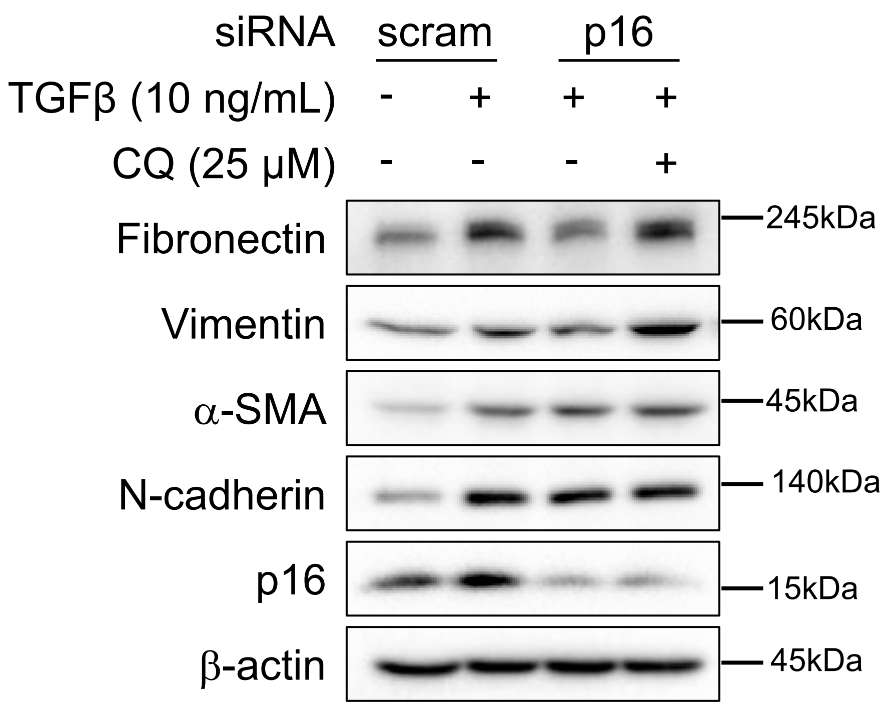

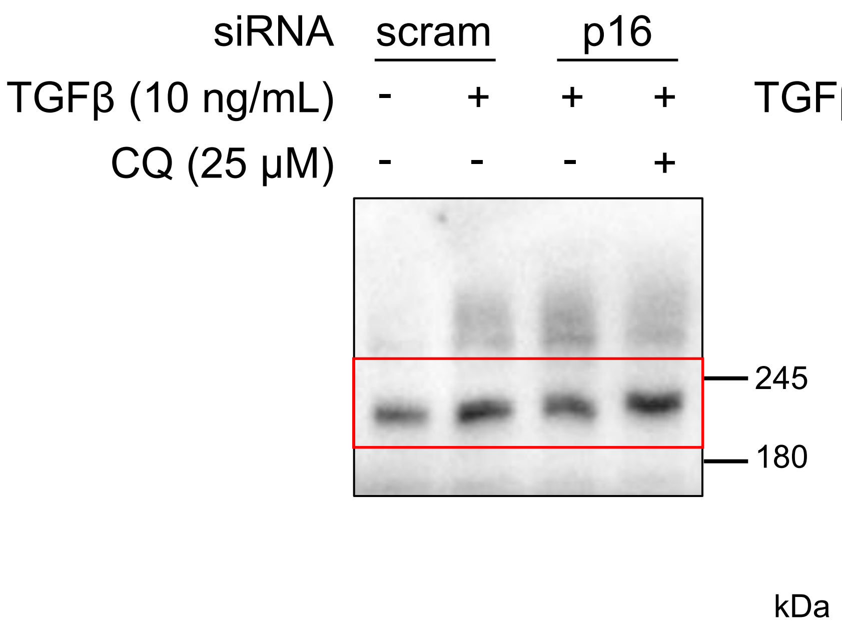

IB: Fibronectin (240kDa)

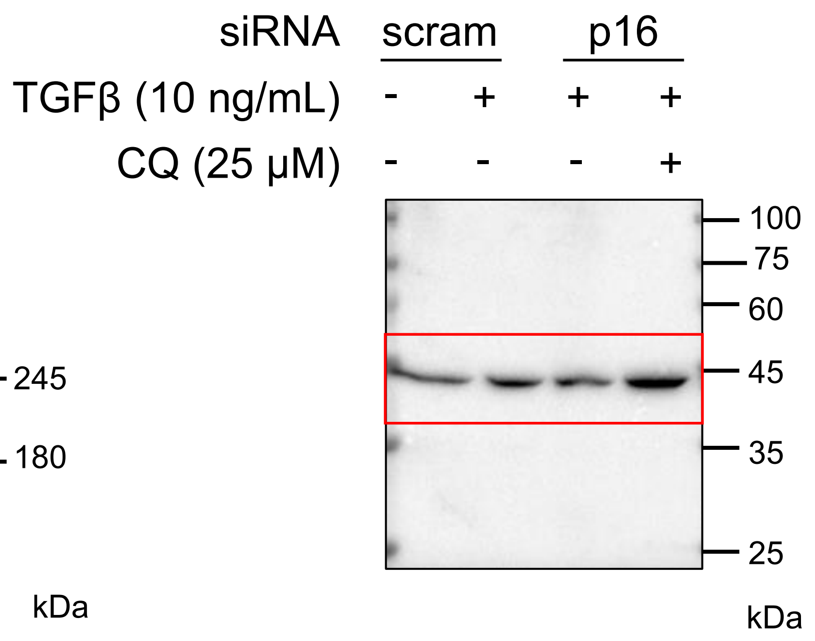

IB: Vimentin (57kDa)

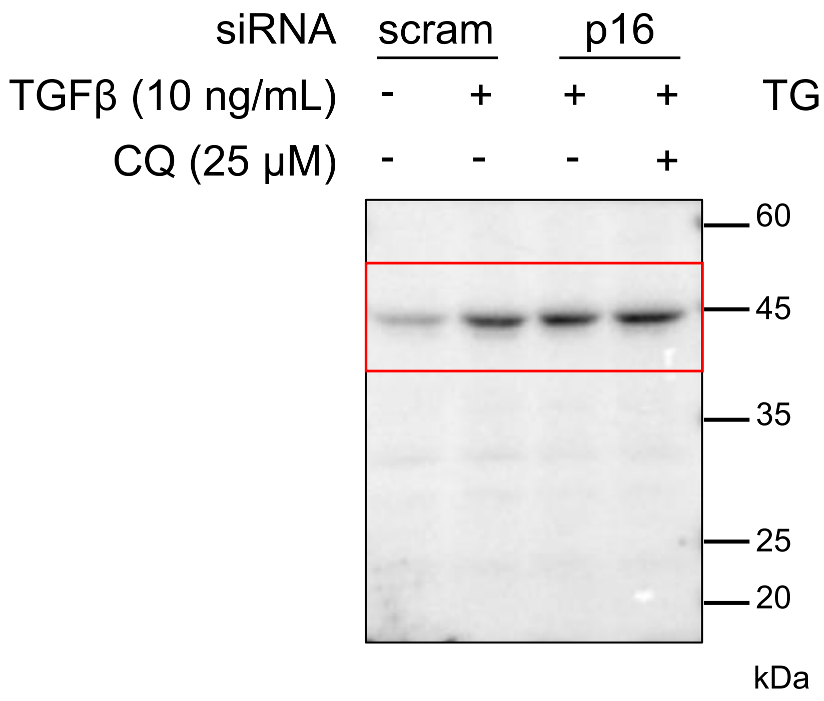

IB: α-SMA (42kDa)

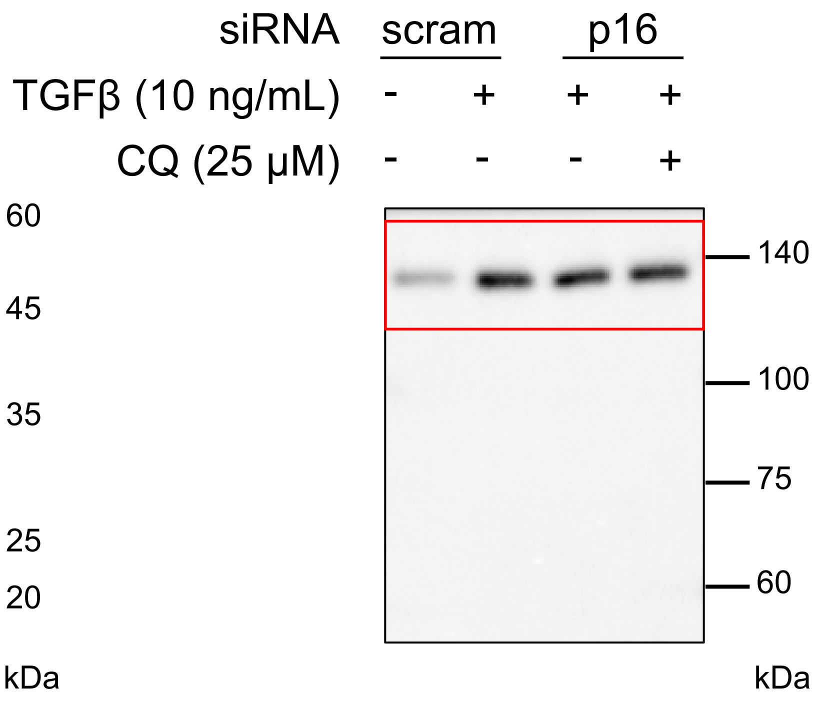

IB: N-cadherin (130kDa)

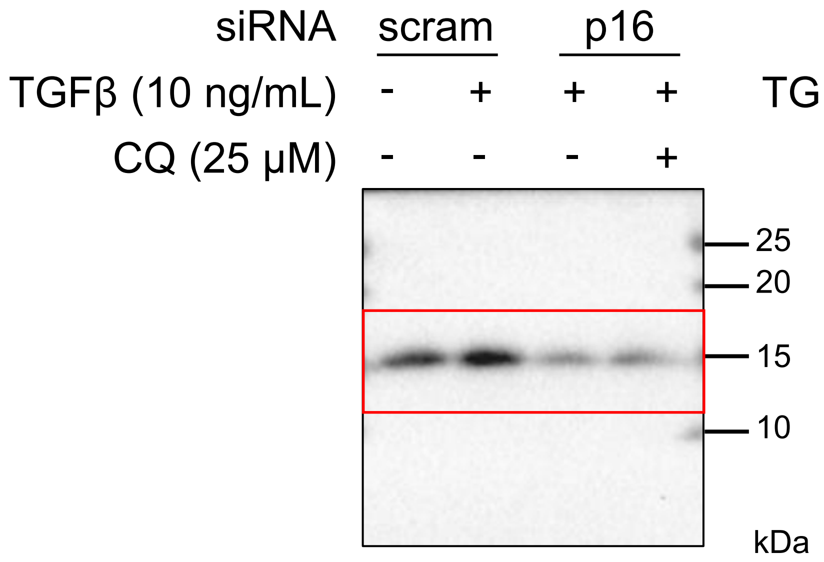

IB: p16 (16kDa)

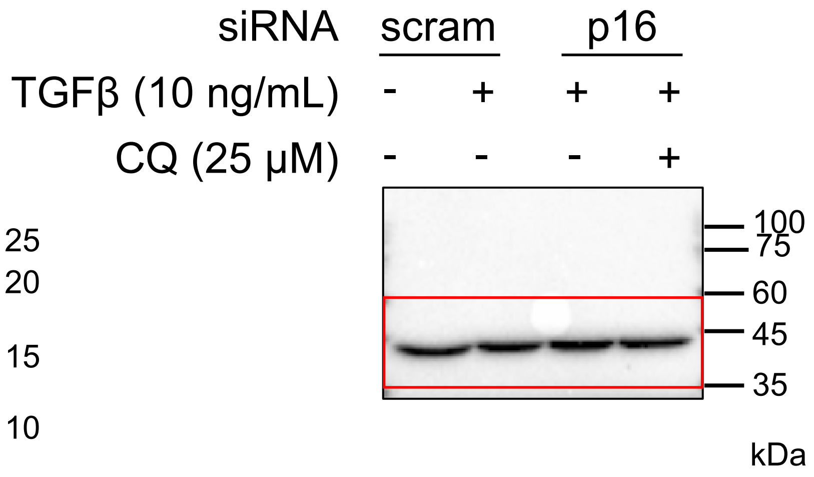

IB: β-actin (45kDa)

Supplementary Figure 2 g, N=4

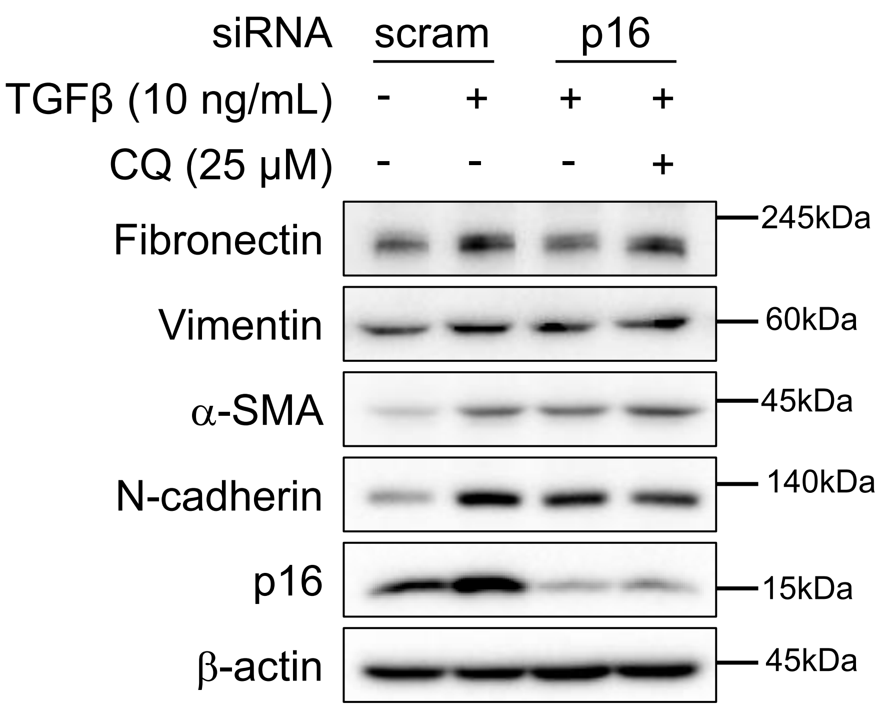

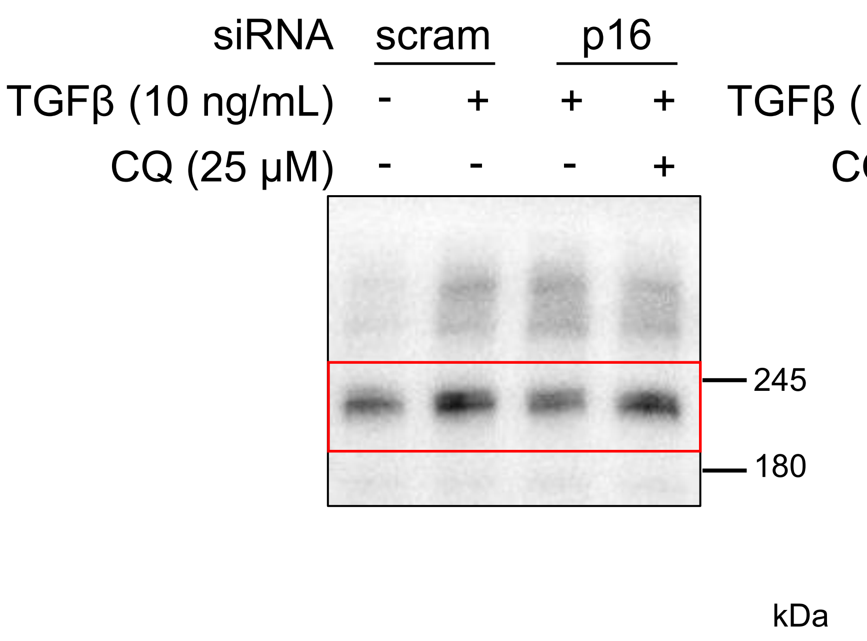

IB: Fibronectin (240kDa)

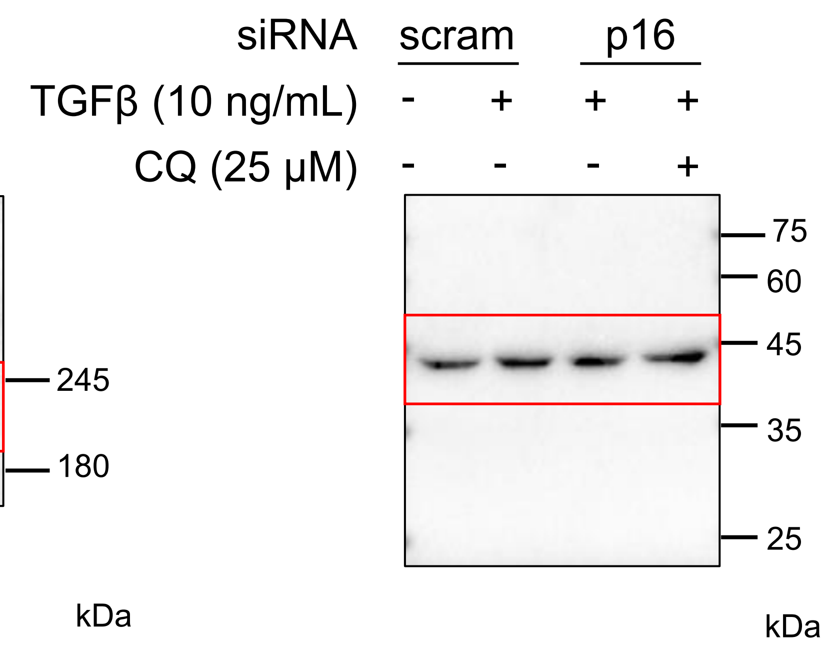

IB: Vimentin (57kDa)

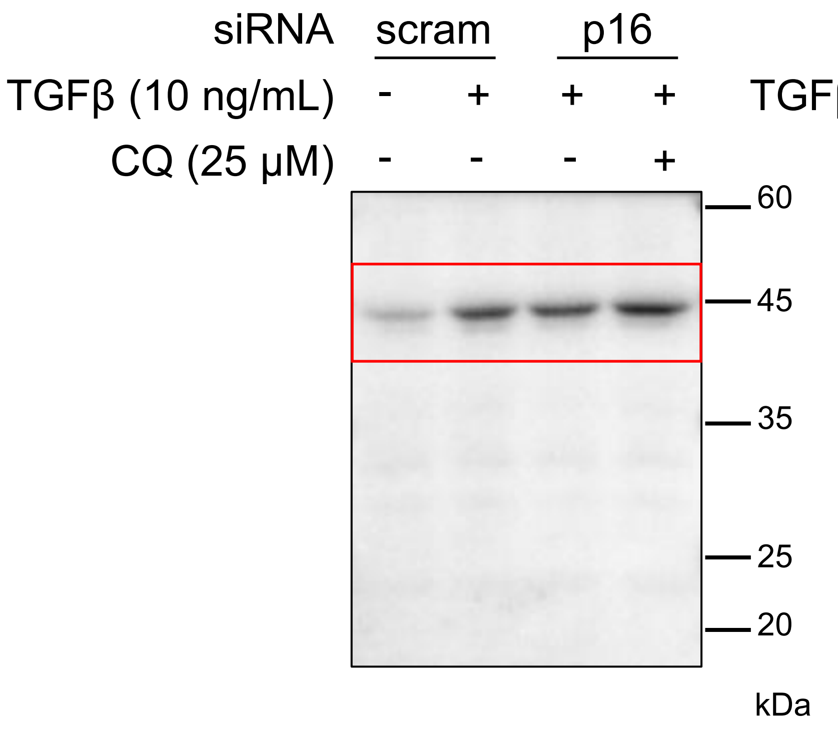

IB: α-SMA (42kDa)

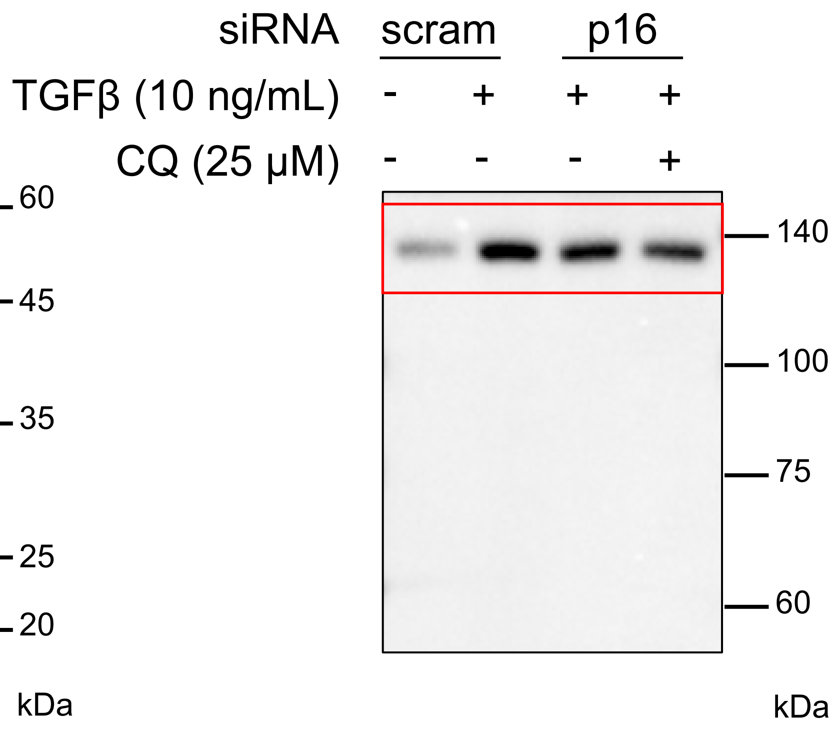

IB: N-cadherin (130kDa)

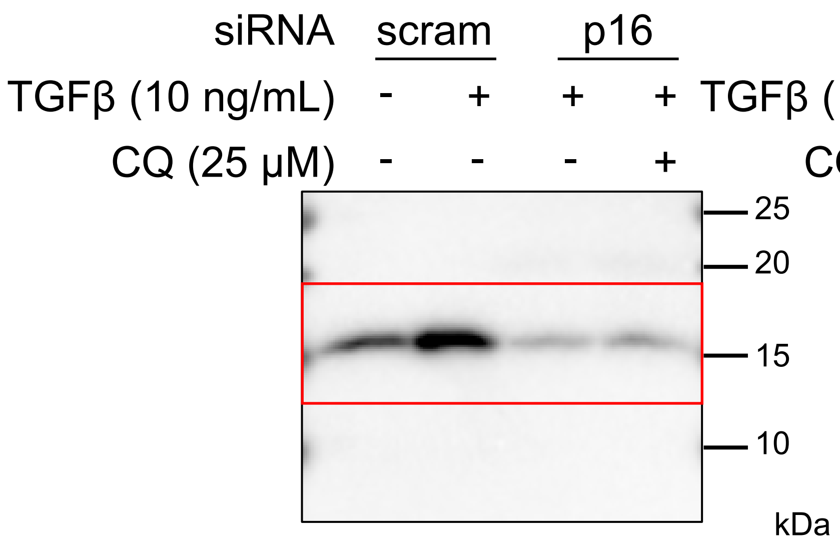

IB: p16 (16kDa)

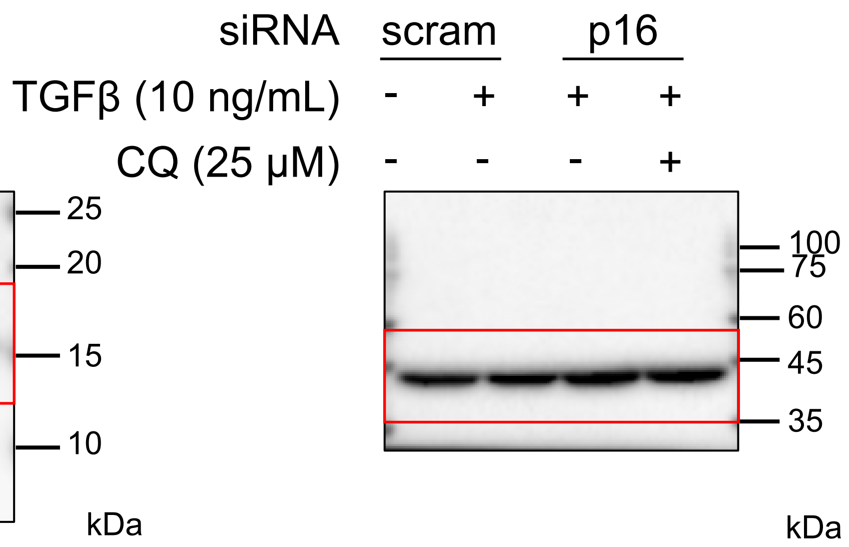

IB: β-actin (45kDa)

Supplementary Figure 2 g, N=5

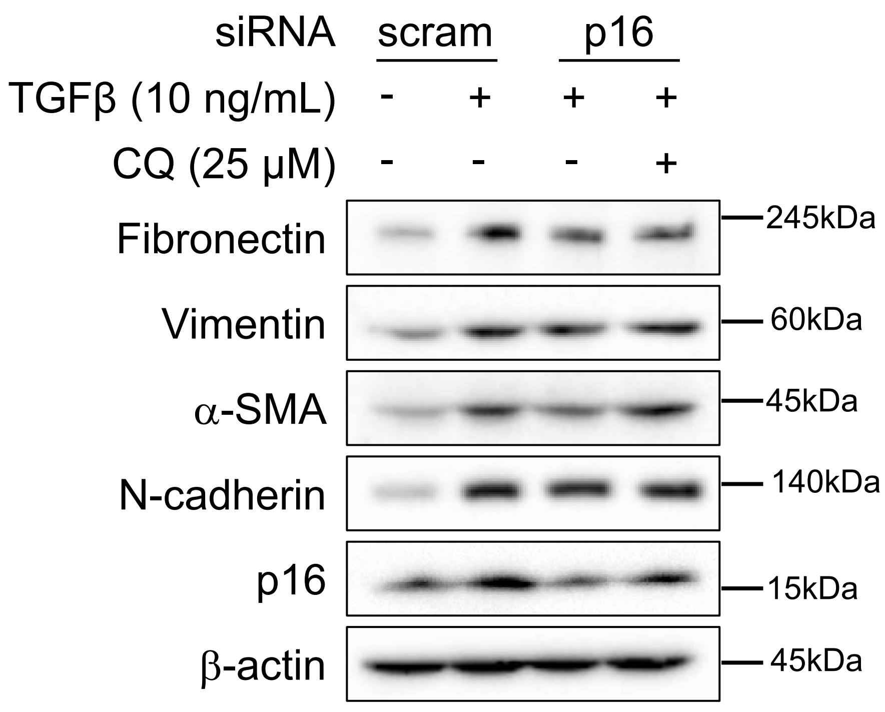

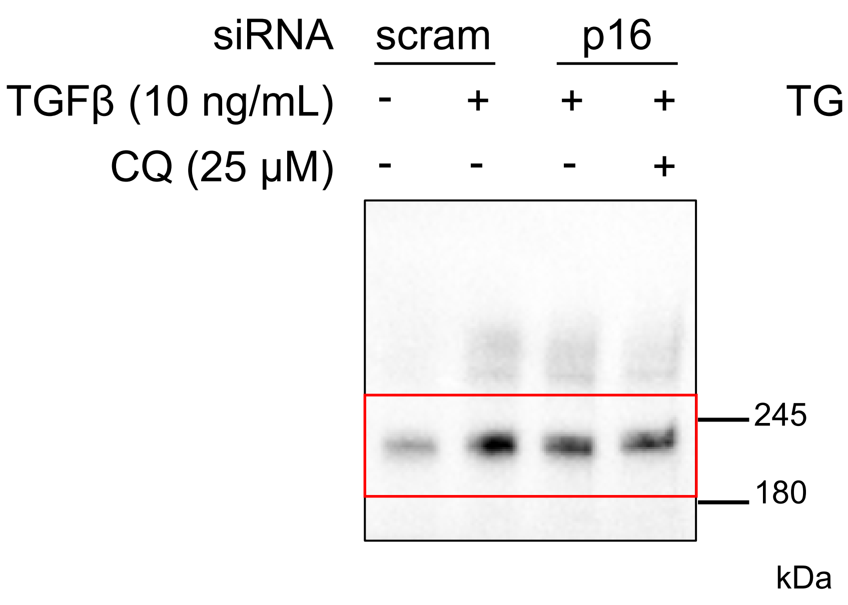

IB: Fibronectin (240kDa)

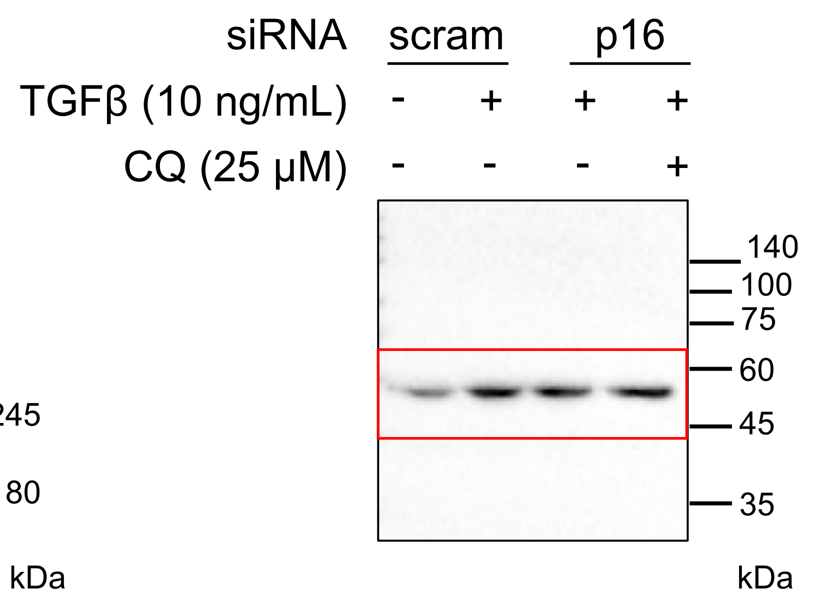

IB: Vimentin (57kDa)

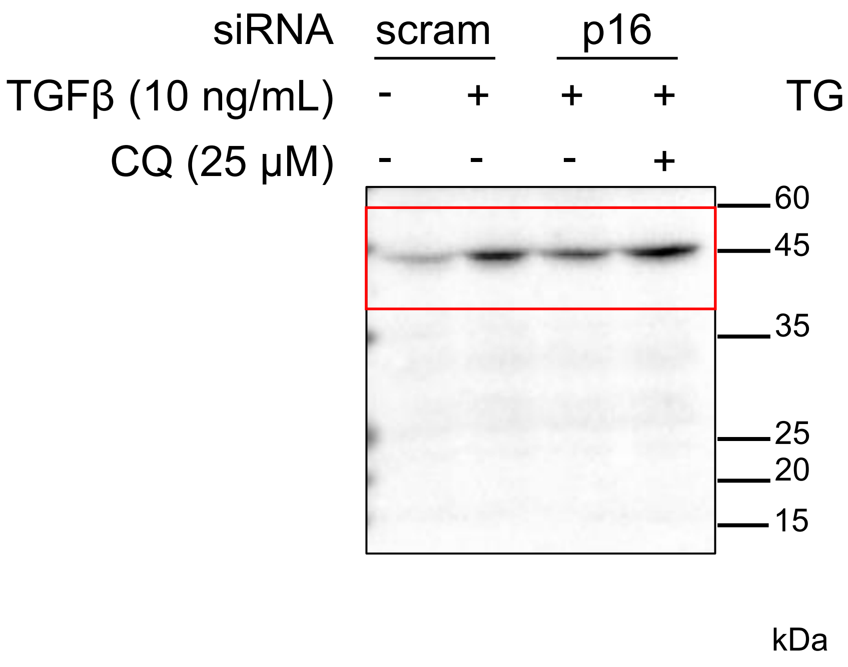

IB: α-SMA (42kDa)

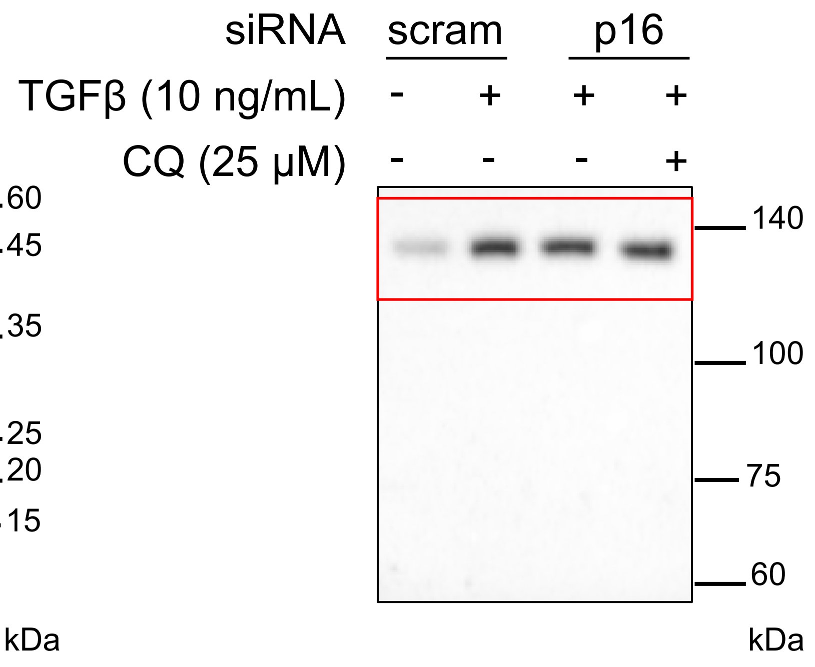

IB: N-cadherin (130kDa)

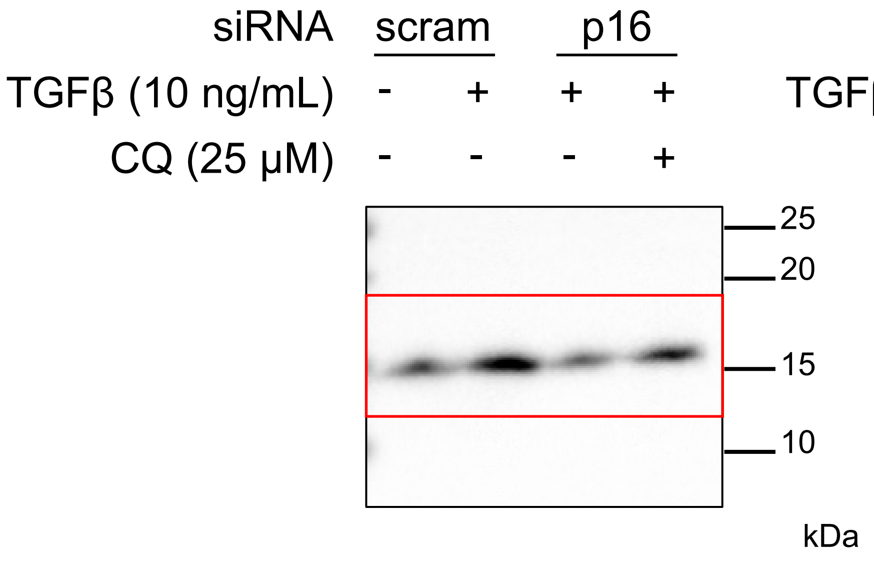

IB: p16 (16kDa)

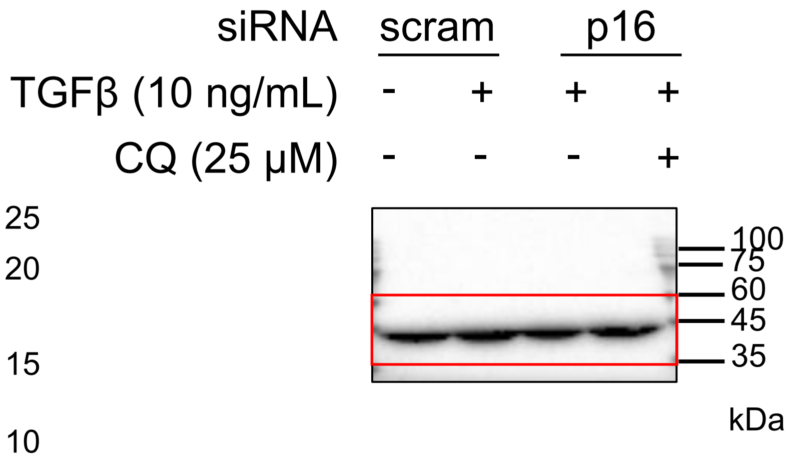

IB: β-actin (45kDa)

Supplementary Figure 3 f, N=1

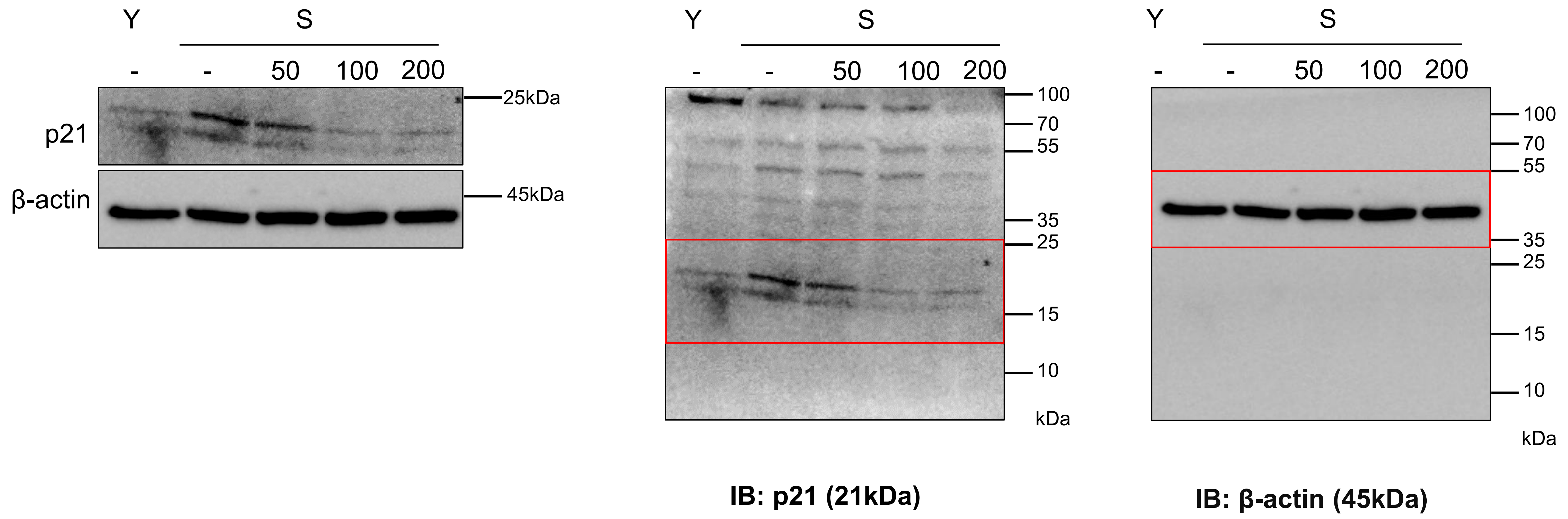

Supplementary Figure 3 f, N=2

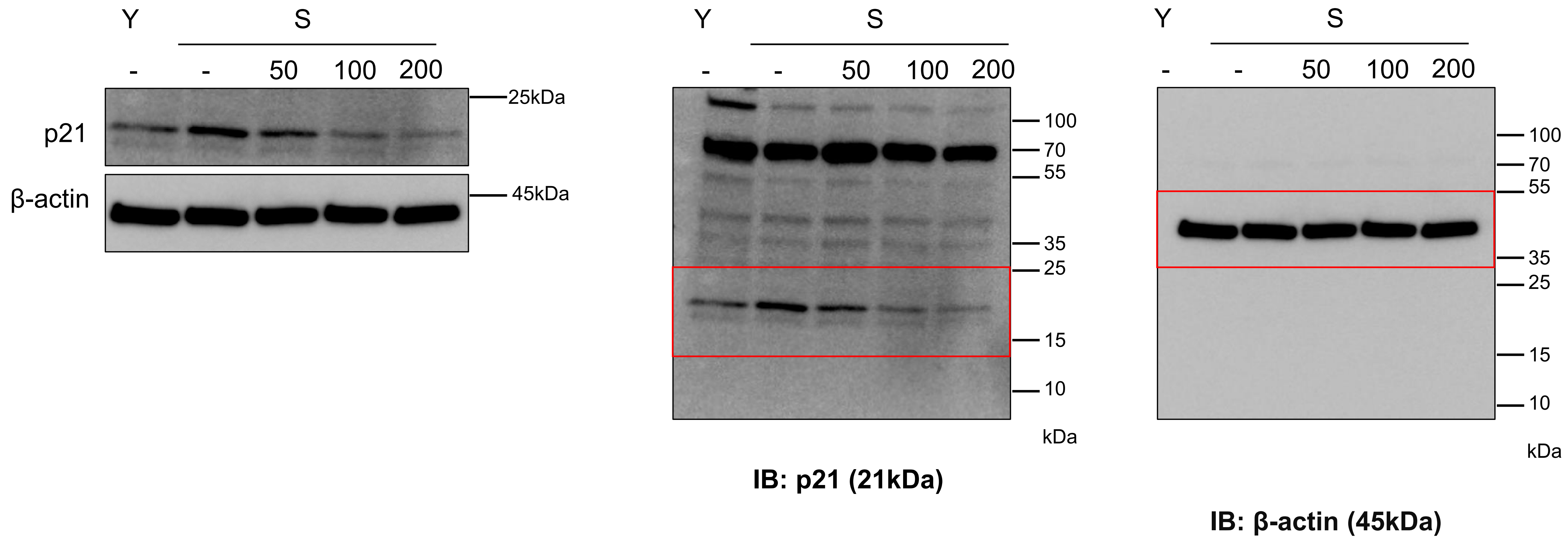

Supplementary Figure 3 f, N=3

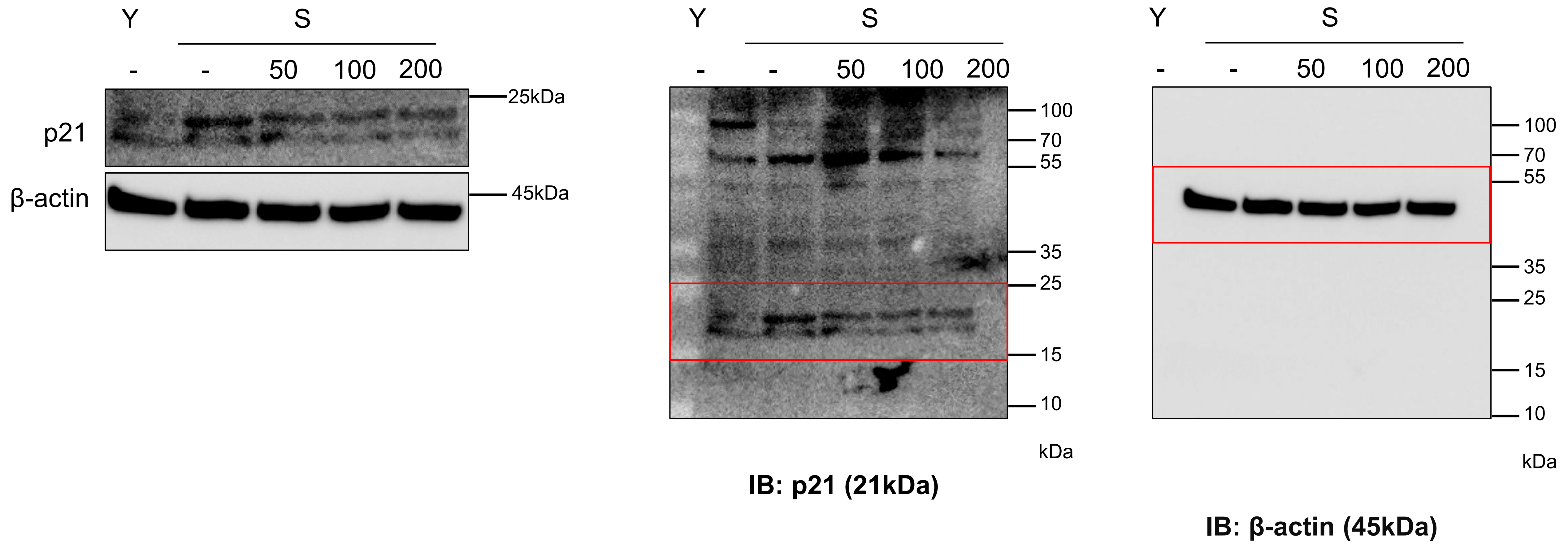

Supplementary Figure 4 a

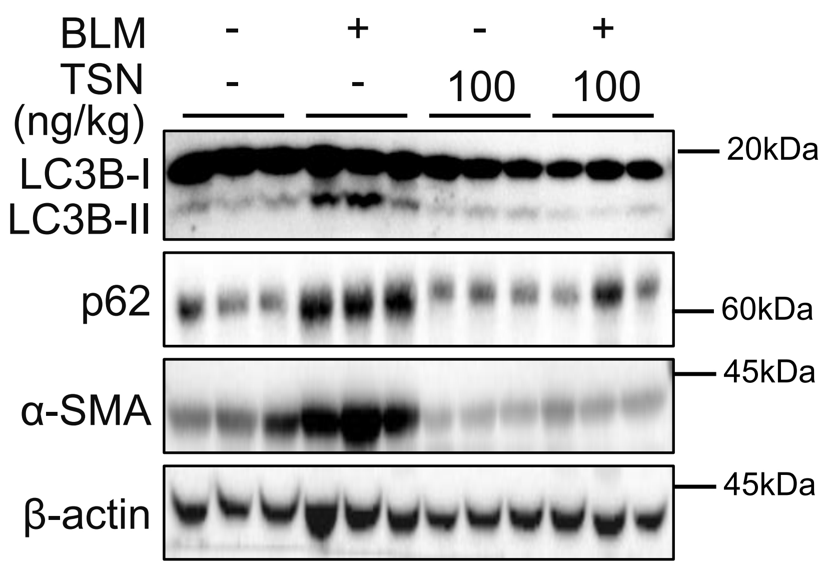

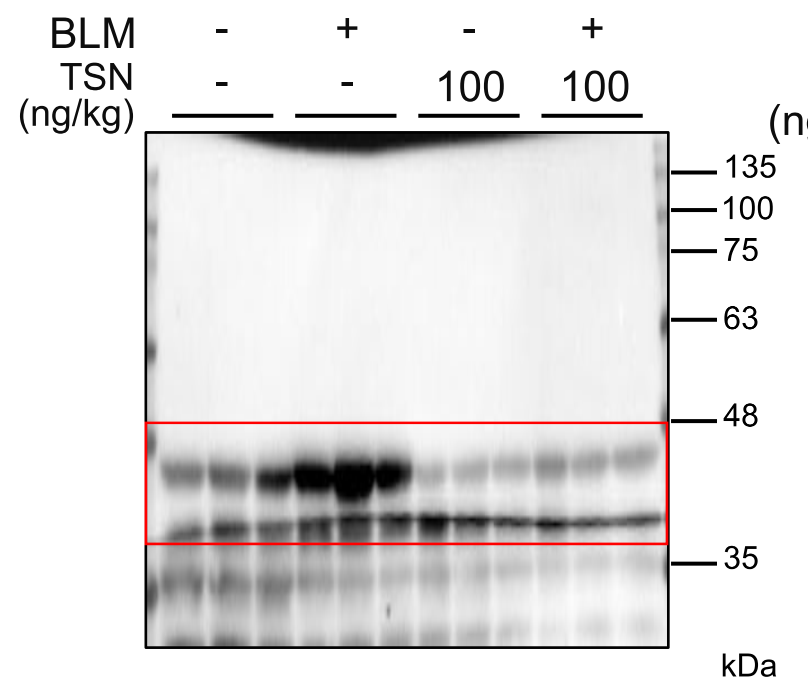

IB: α-SMA (42kDa)

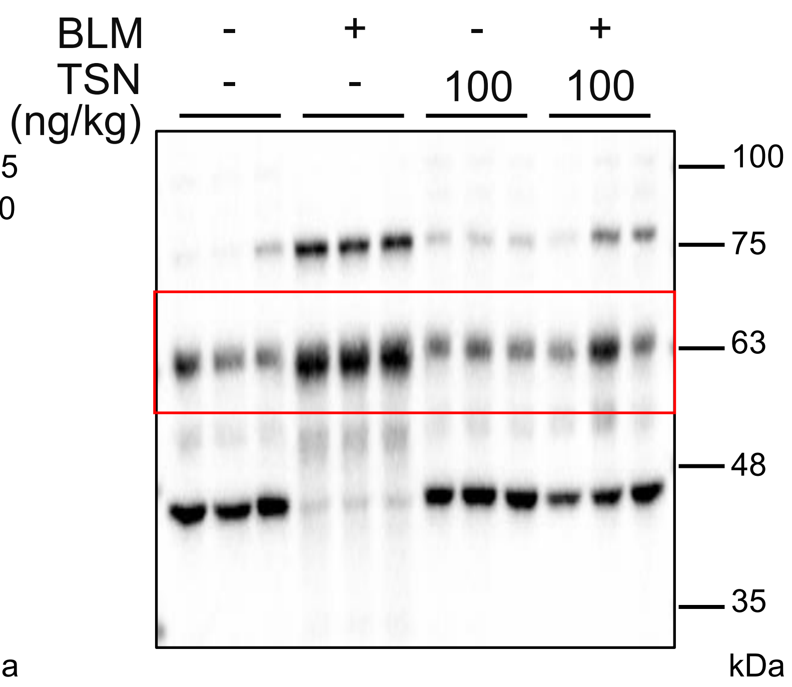

IB: p62 (62kDa)

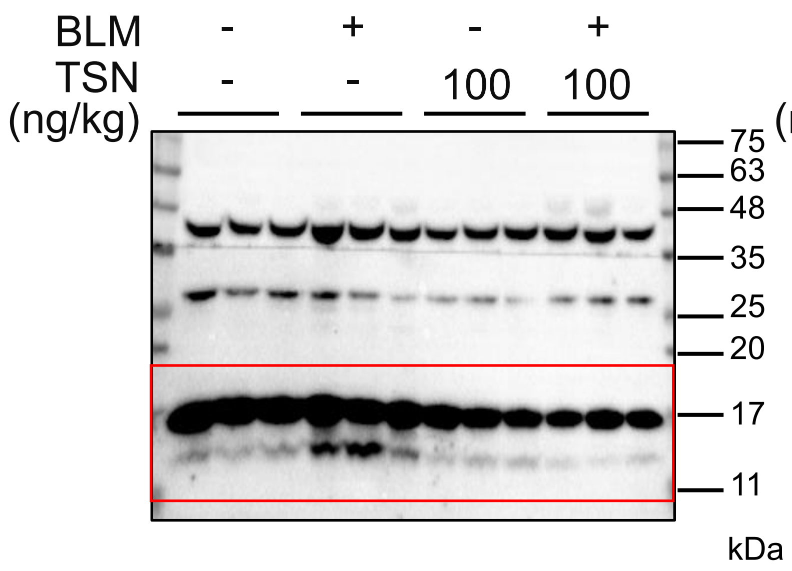

IB: LC3B (16, 14kDa)

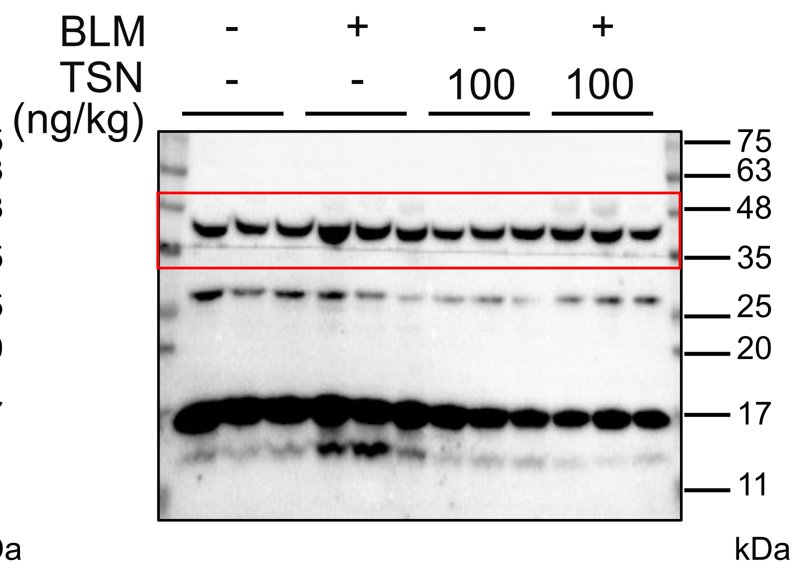

IB: β-actin (45kDa)

Supplementary Figure 5 e, N=1

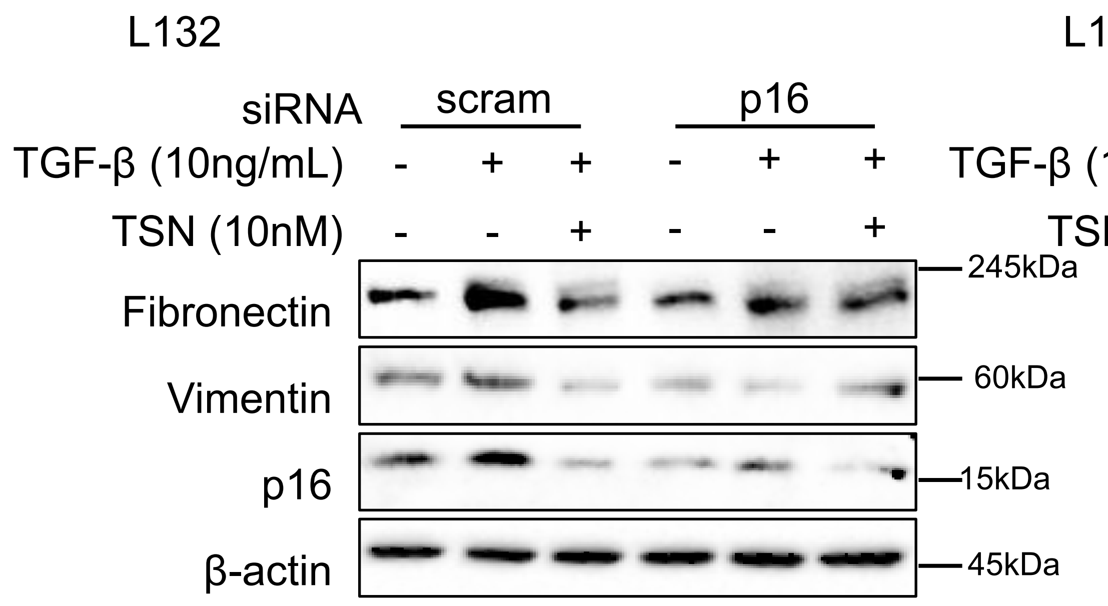

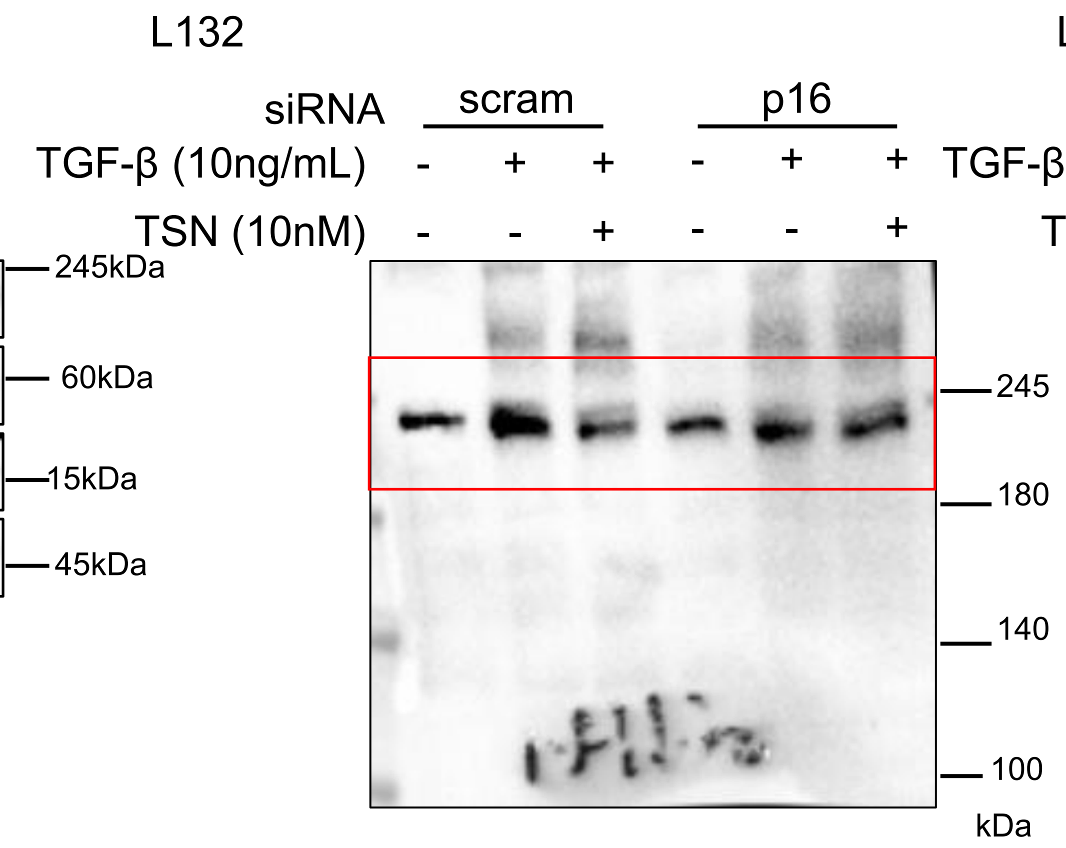

IB: Fibronectin (240kDa)

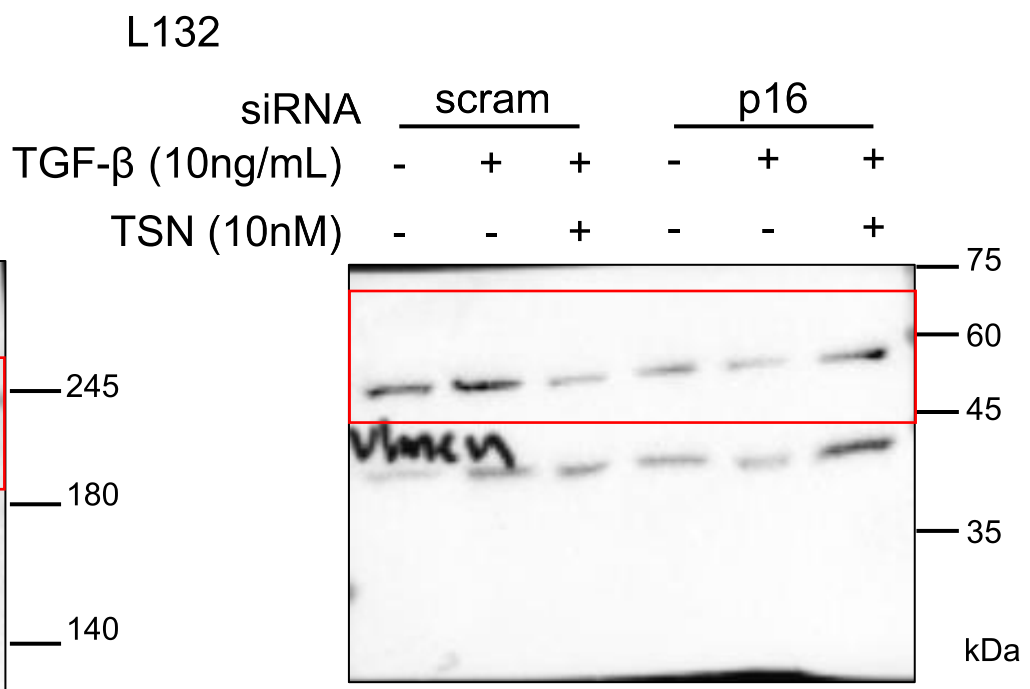

IB: Vimentin (57kDa)

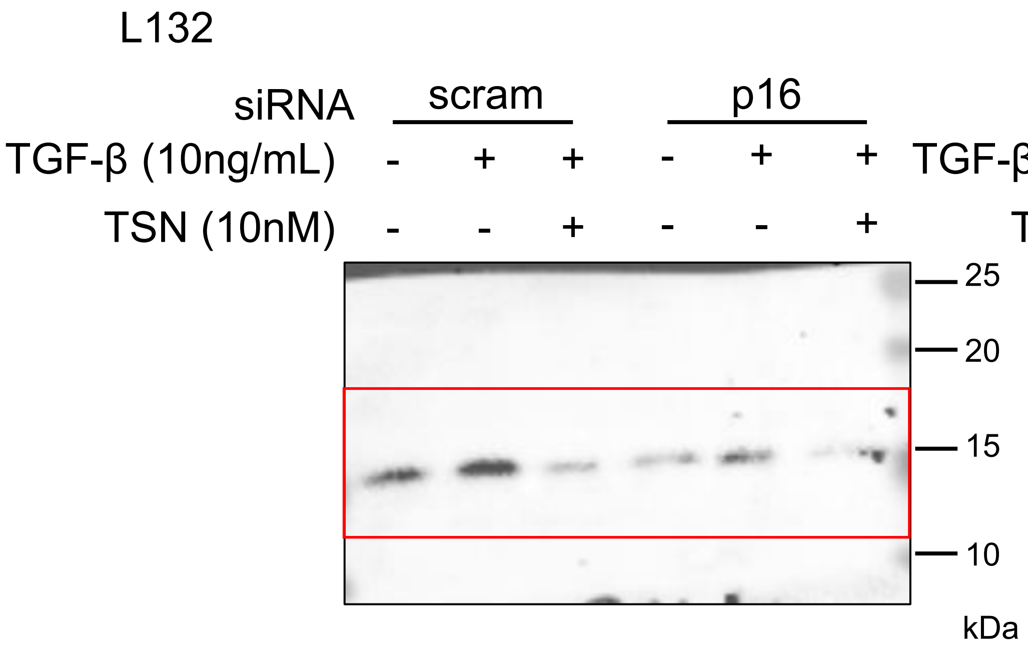

IB: p16 (16kDa)

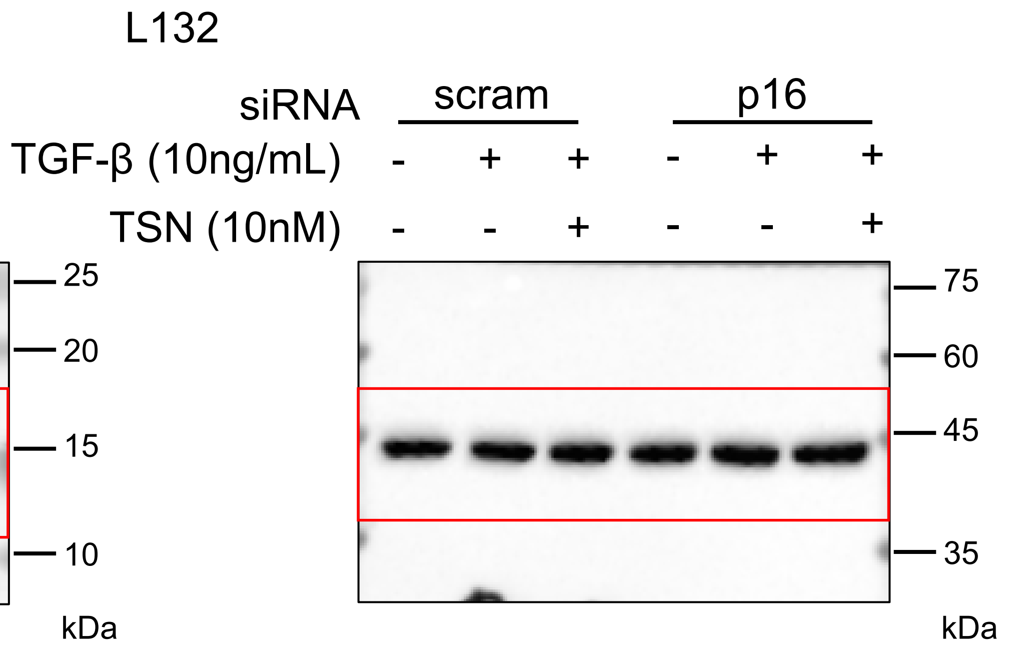

IB: β-actin (45kDa)

Supplementary Figure 5 e, N=2

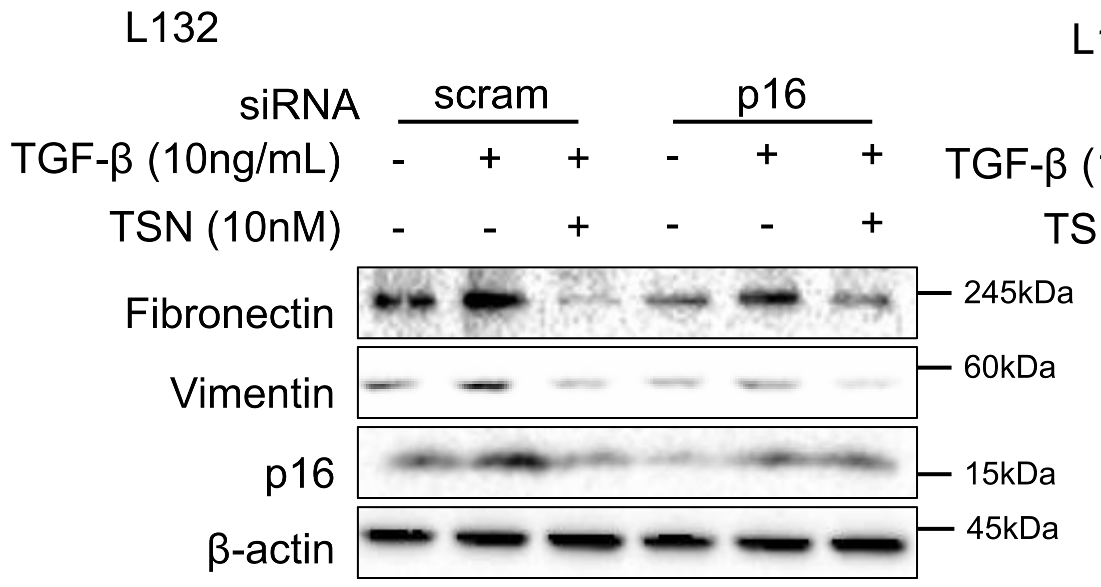

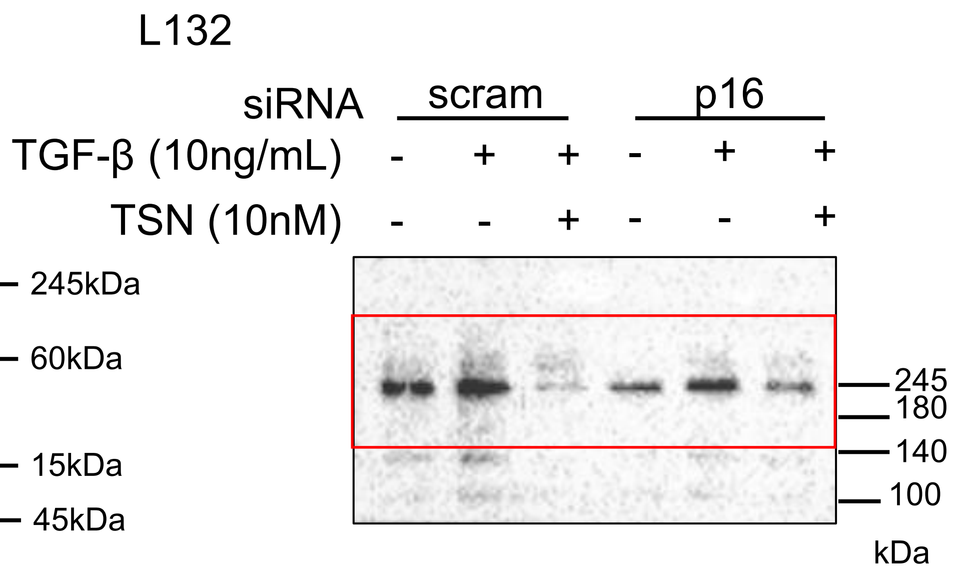

IB: Fibronectin (240kDa)

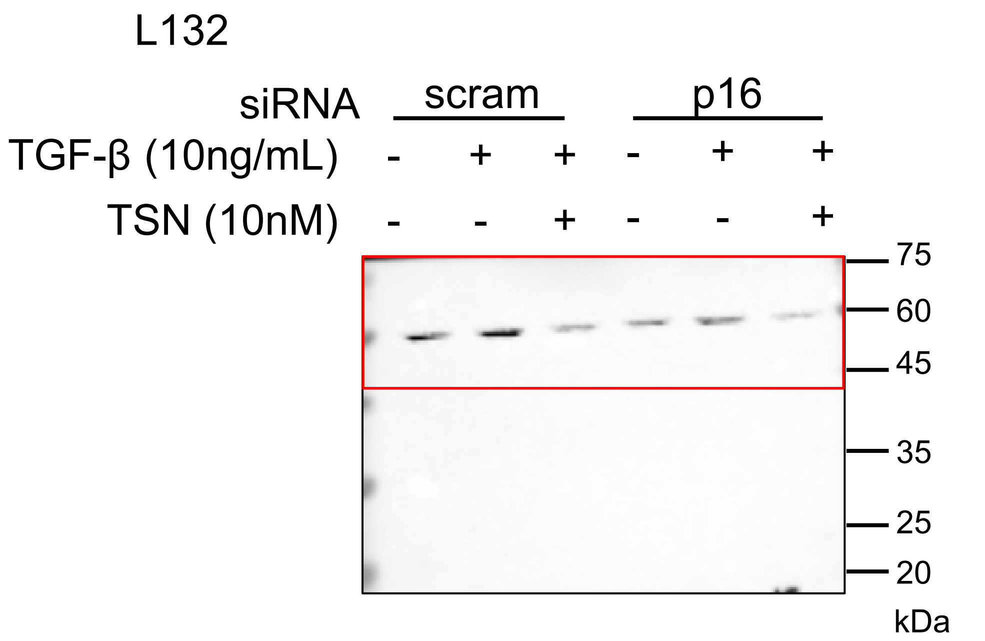

IB: Vimentin (57kDa)

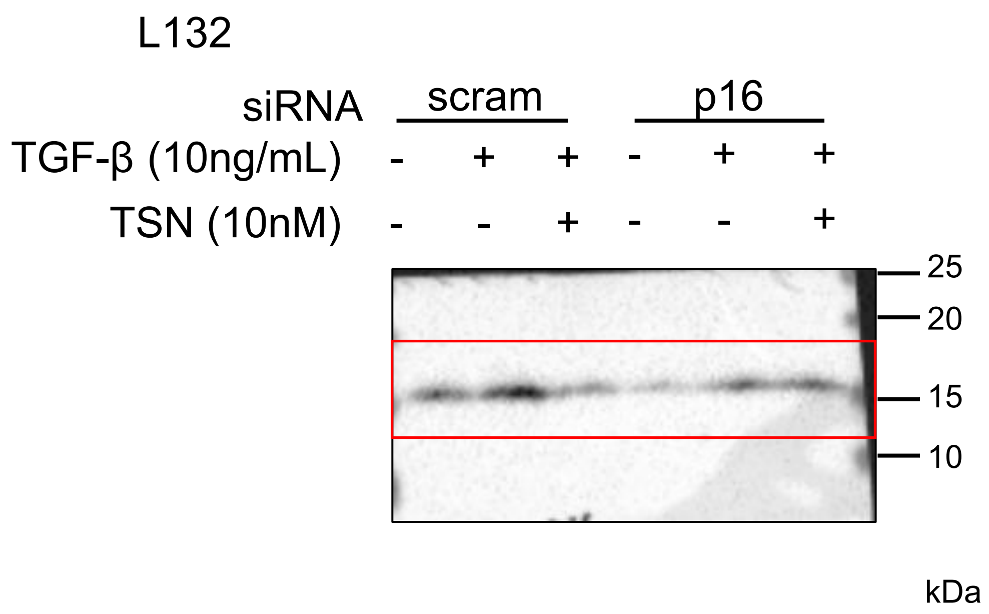

IB: p16 (16kDa)

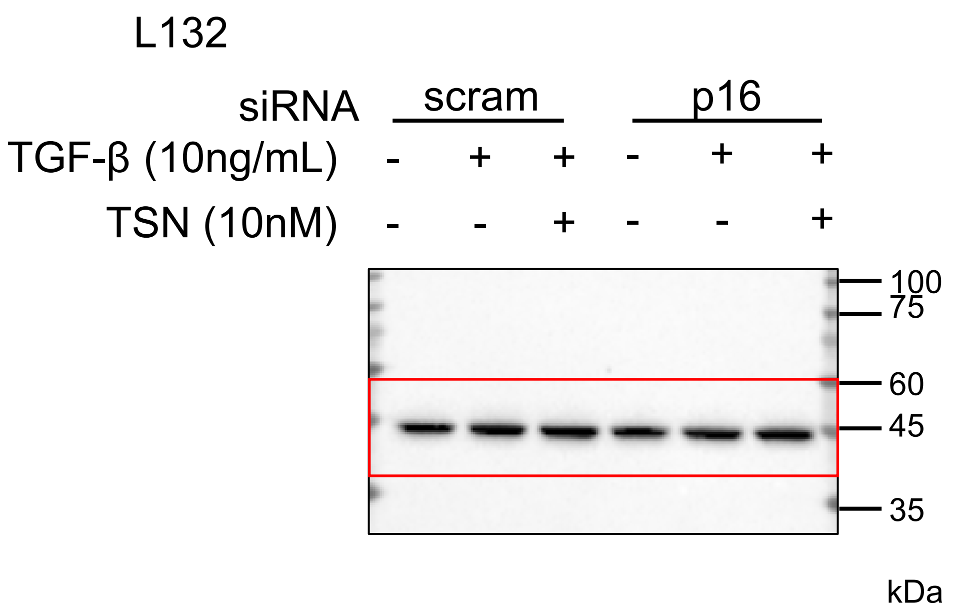

IB: β-actin (45kDa)

Supplementary Figure 5 e, N=3

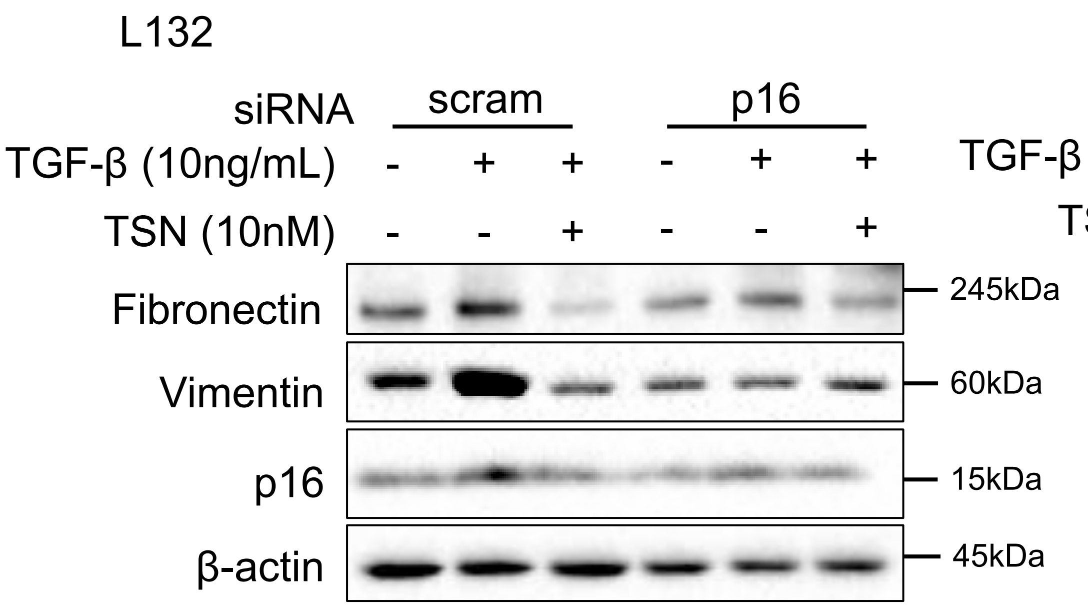

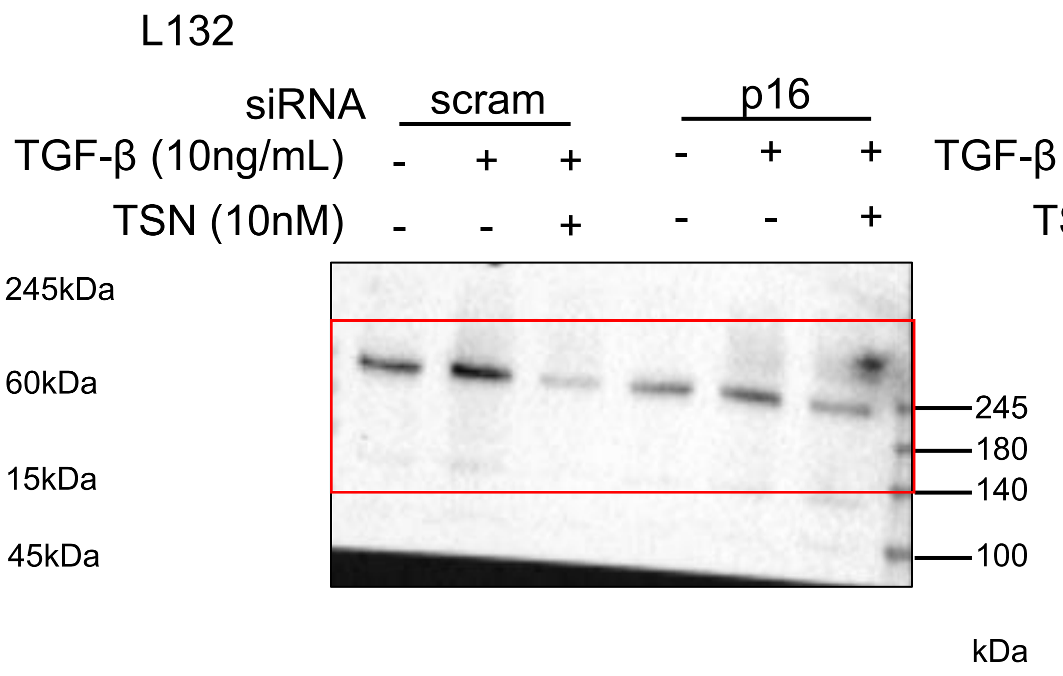

IB: Fibronectin (240kDa)

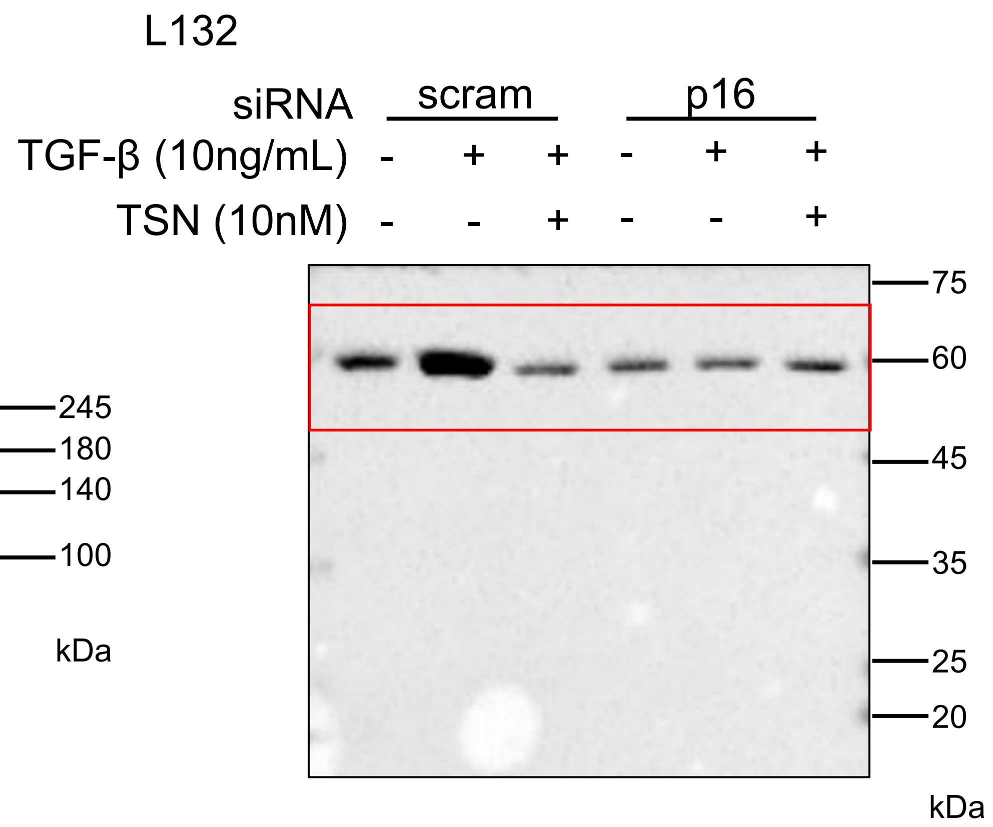

IB: Vimentin (57kDa)

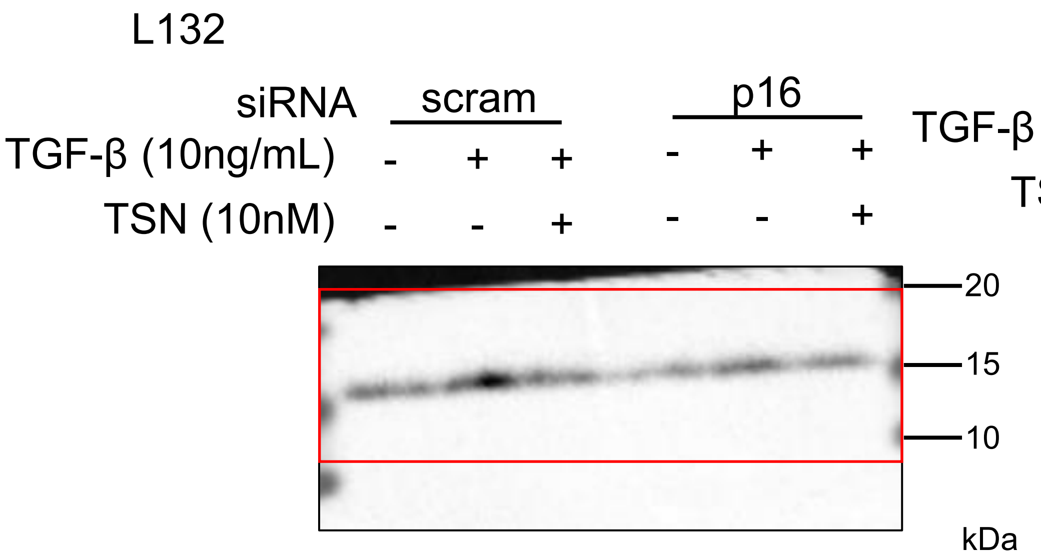

IB: p16 (16kDa)

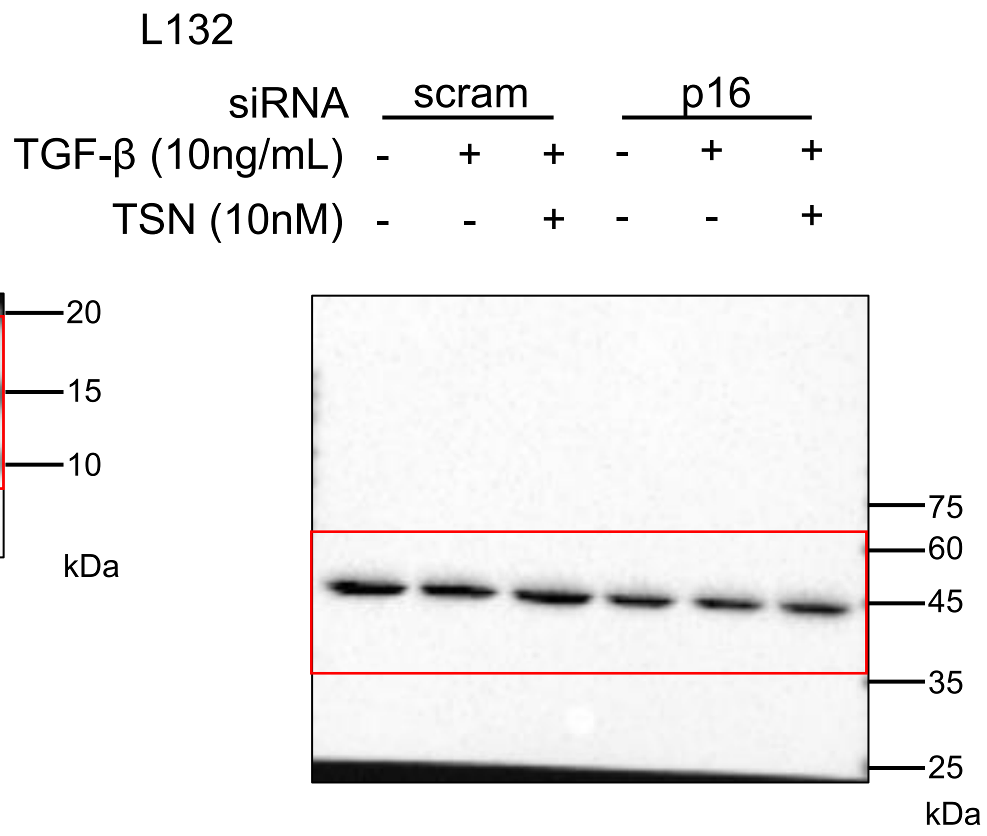

IB: β-actin (45kDa)

Supplementary Figure 5 e, N=4

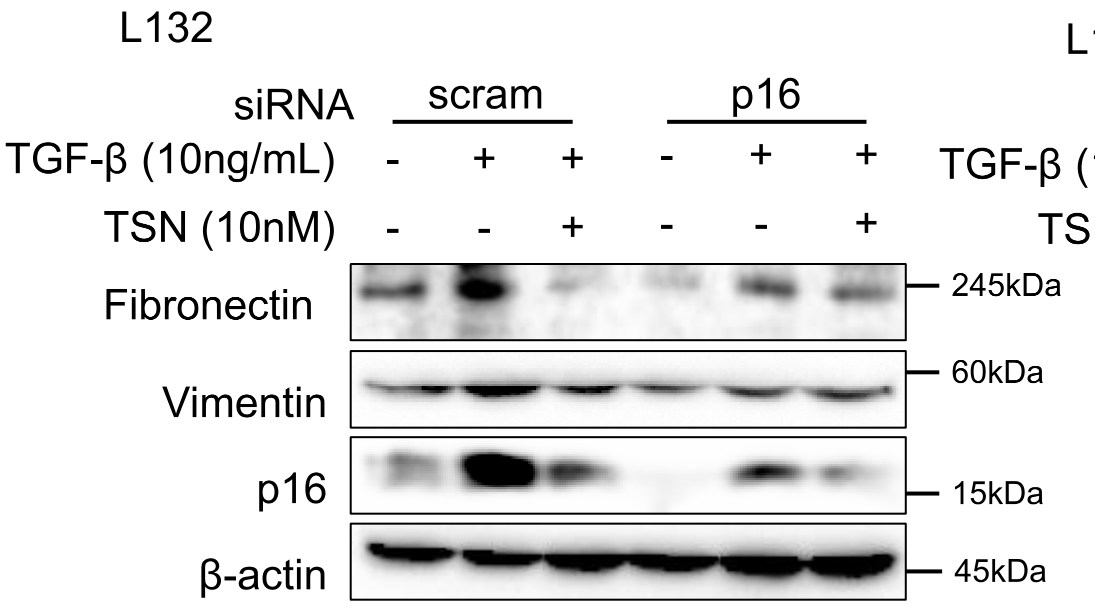

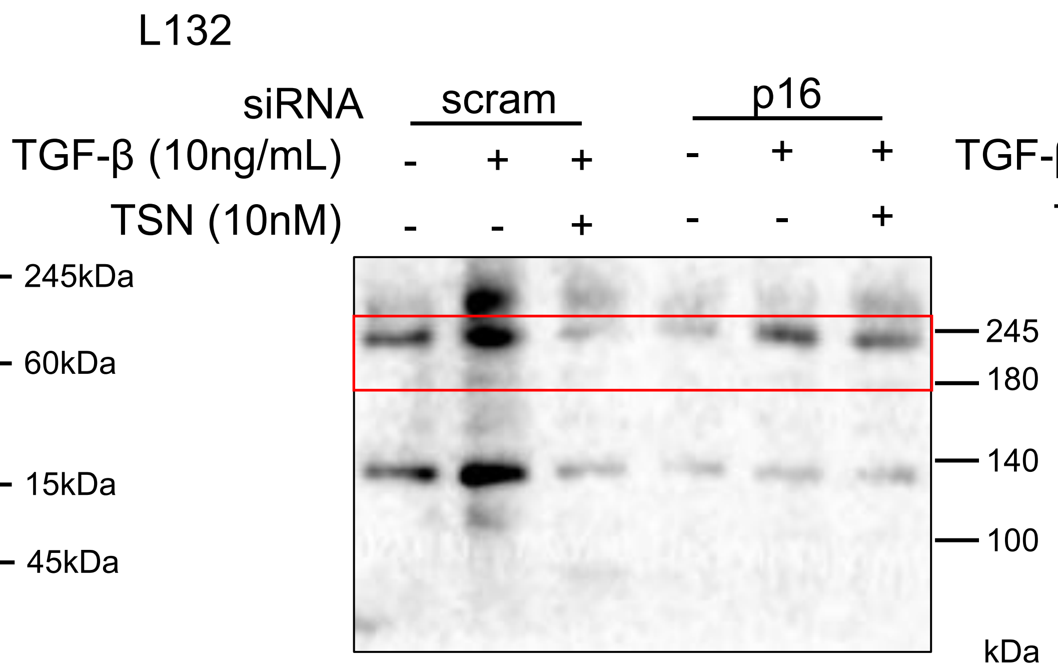

IB: Fibronectin (240kDa)

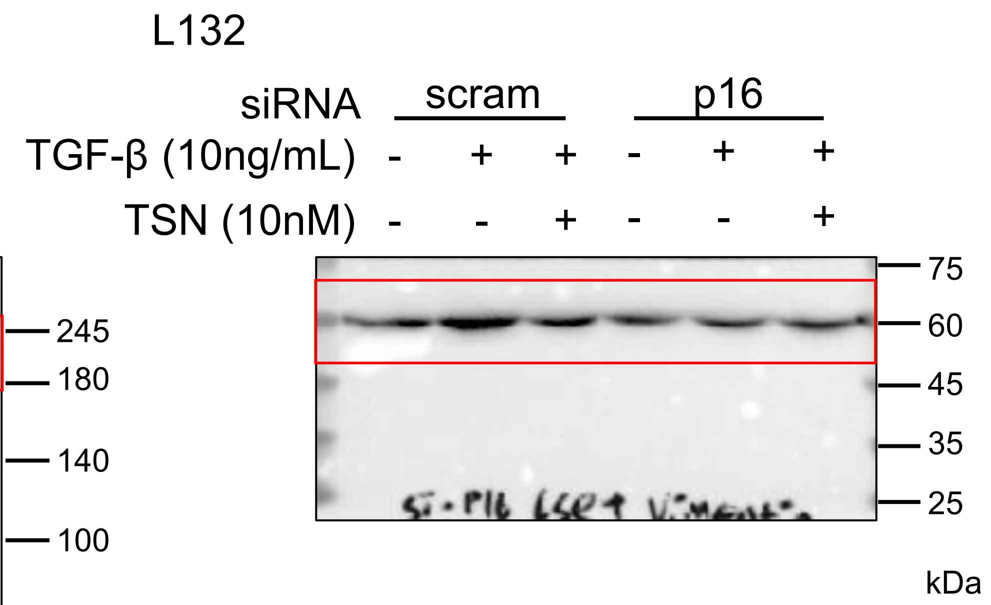

IB: Vimentin (57kDa)

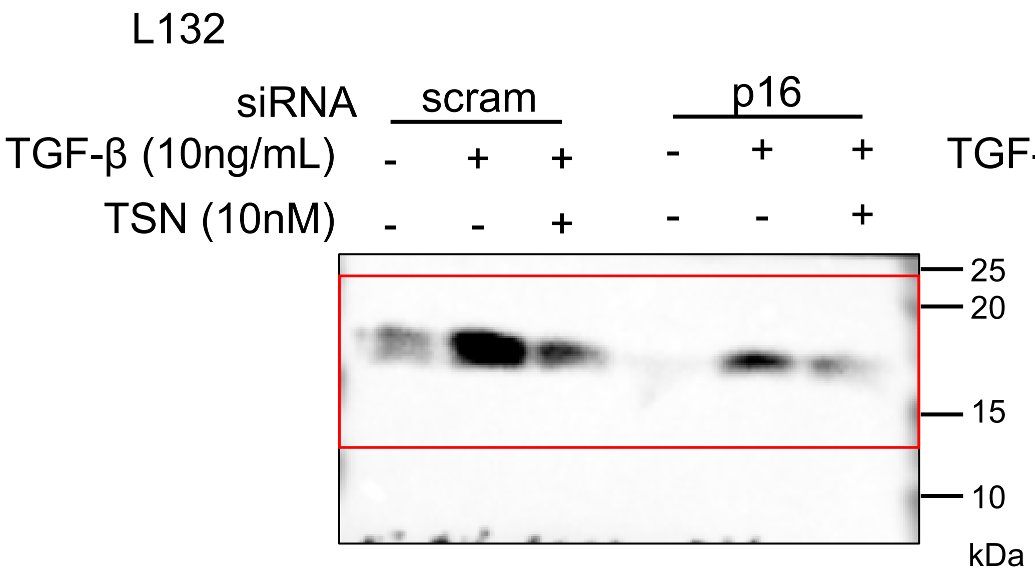

IB: p16 (16kDa)

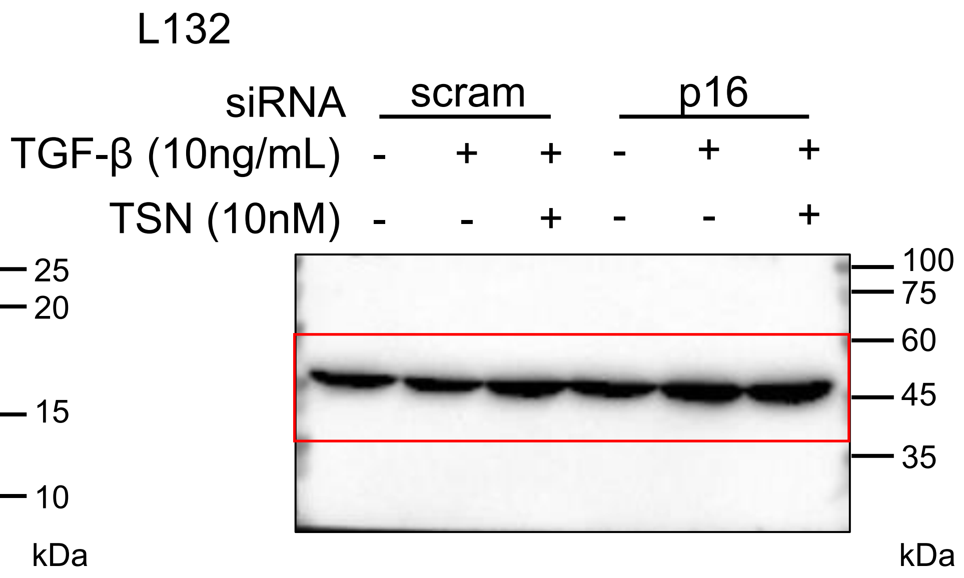

IB: β-actin (45kDa)

Supplementary Figure 5 e, N=5

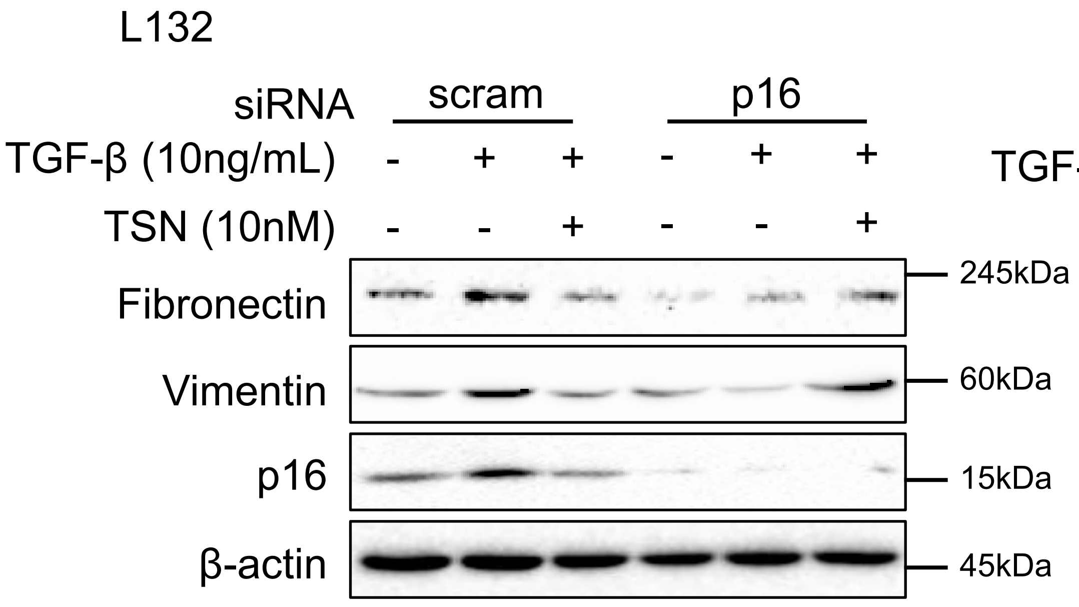

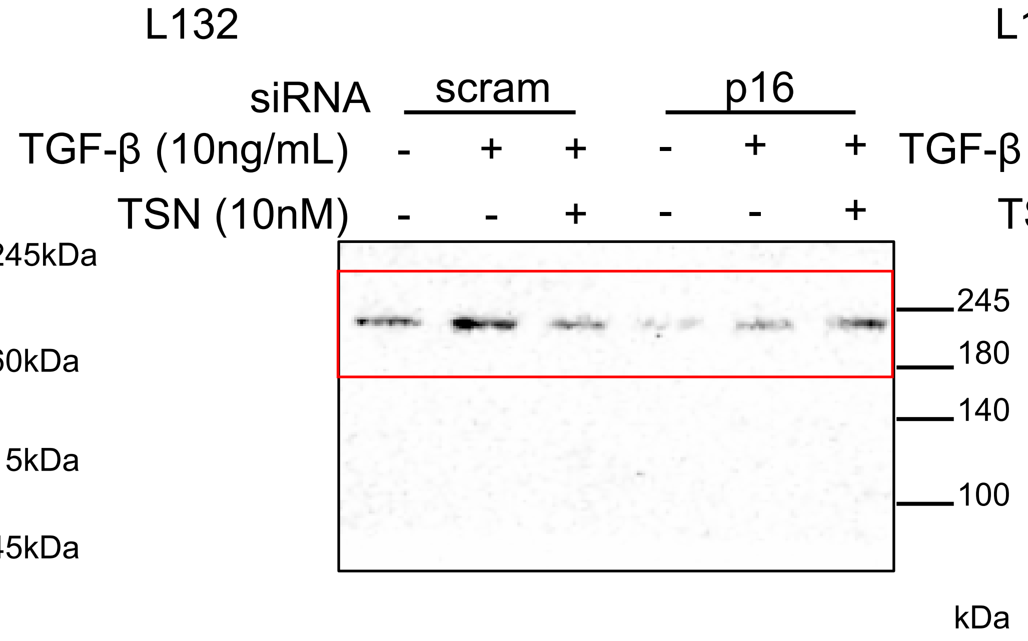

IB: Fibronectin (240kDa)

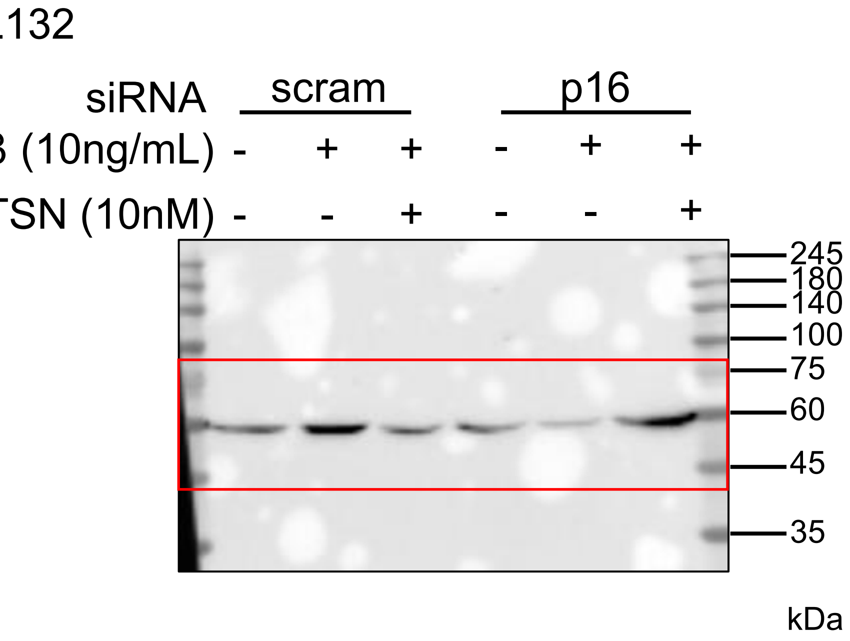

IB: Vimentin (57kDa)

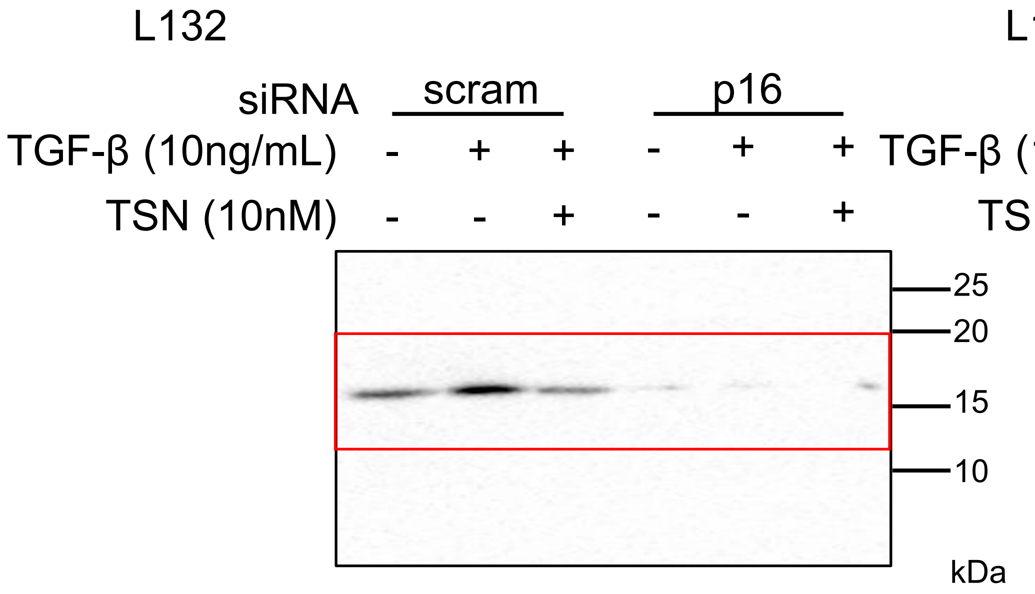

IB: p16 (16kDa)

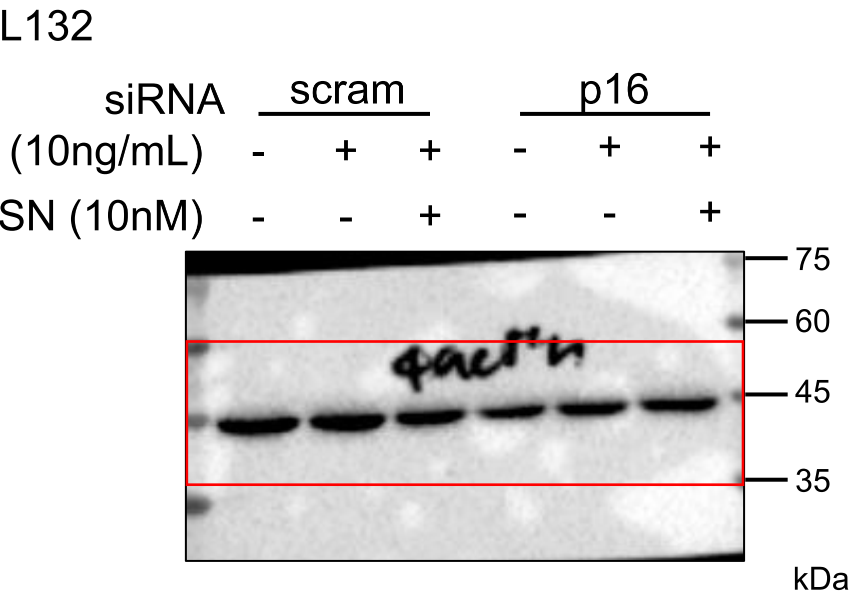

IB: β-actin (45kDa)

Supplementary Figure 7 c

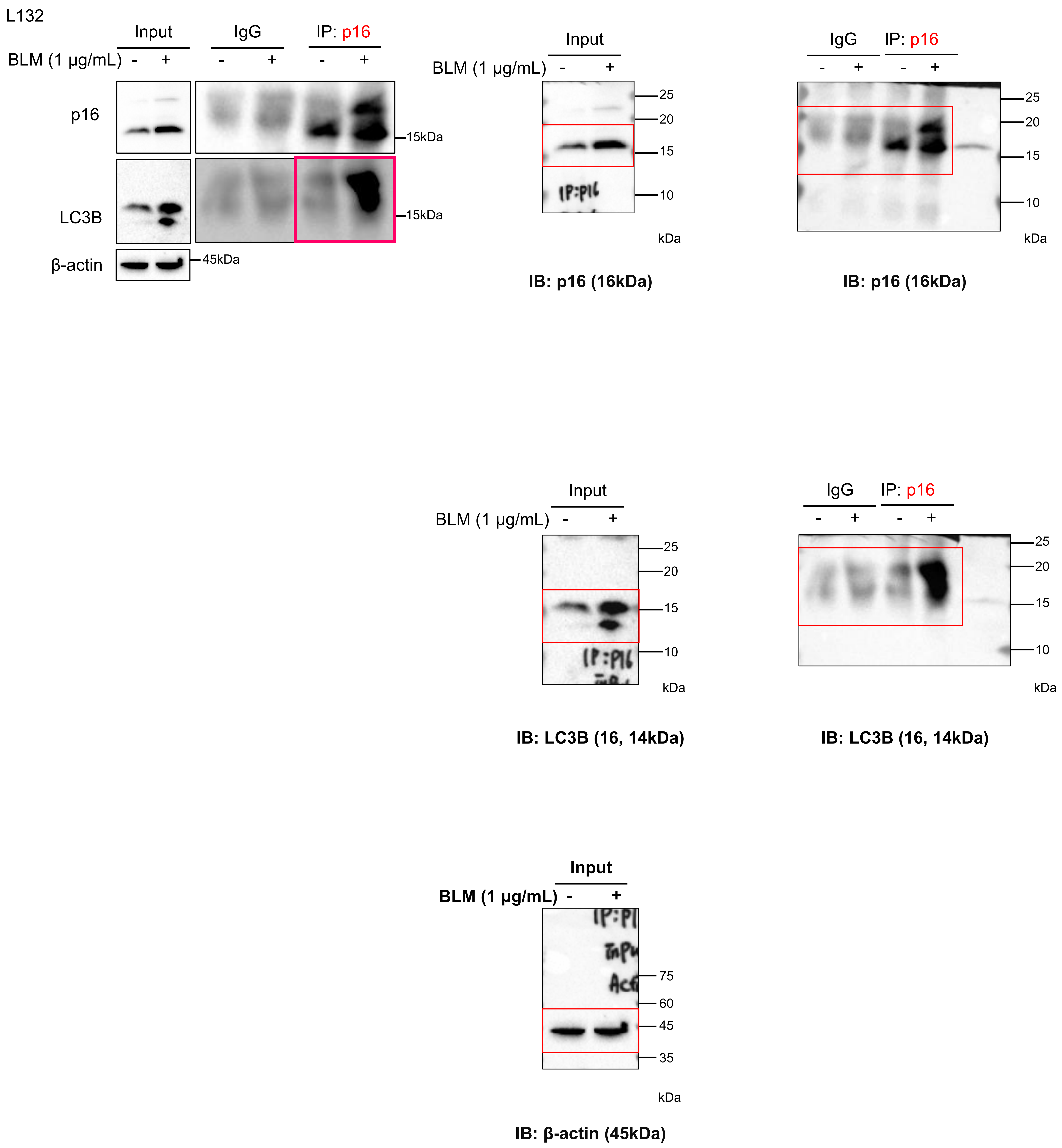

Supplementary Figure 7 c

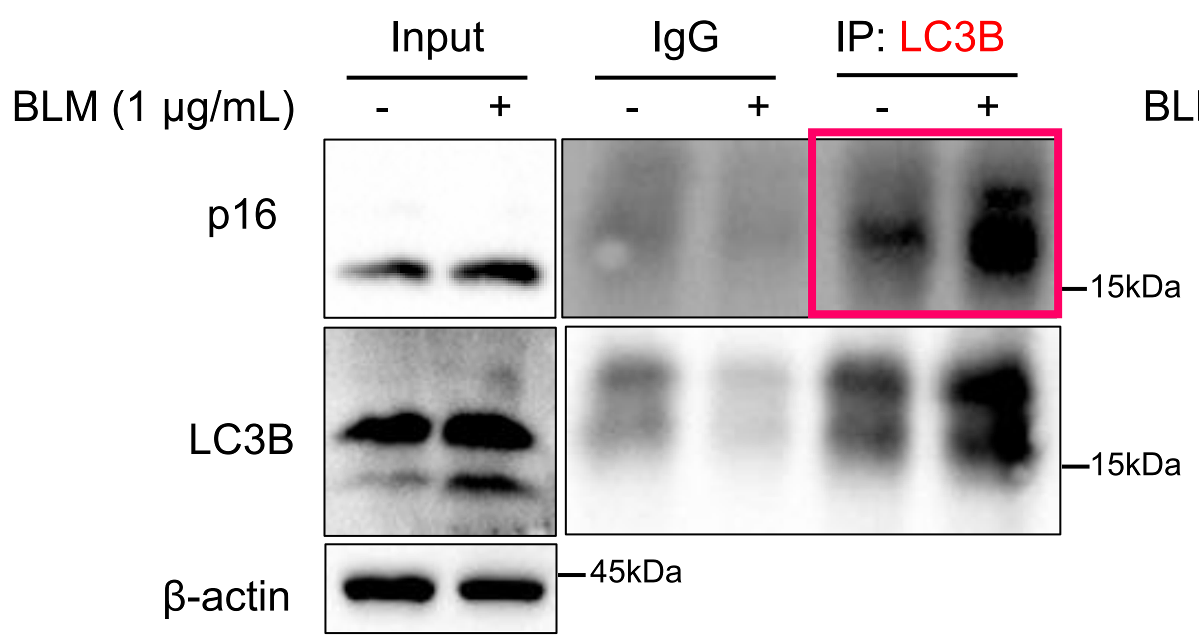

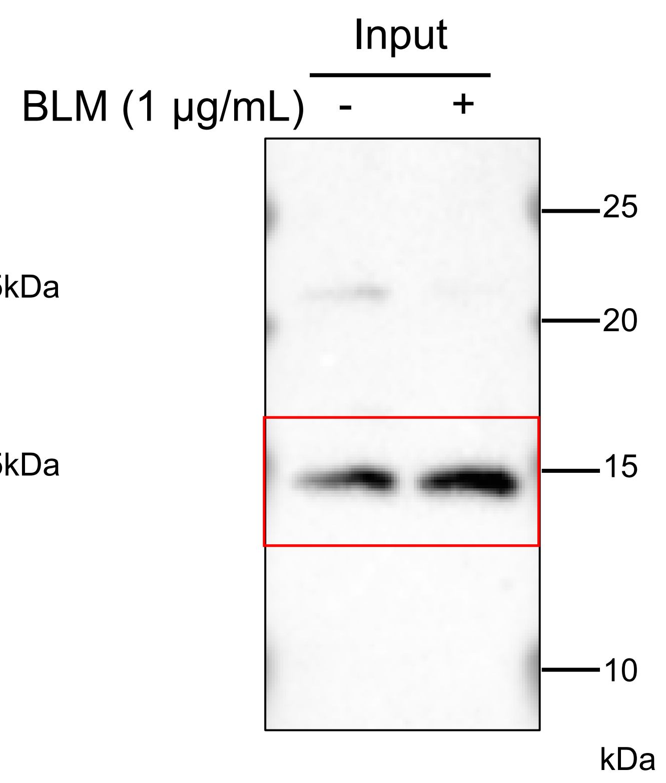

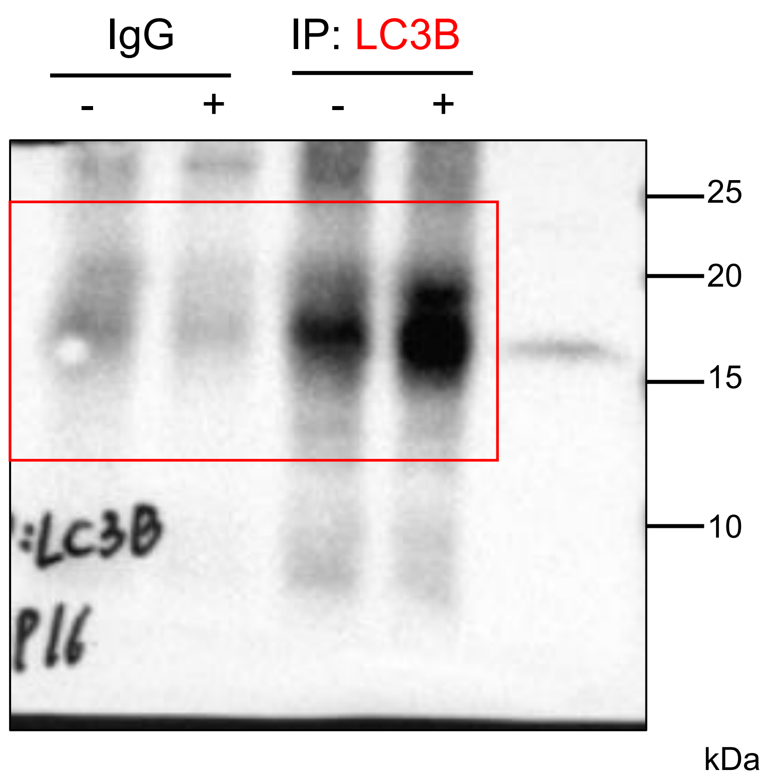

IB: p16 (16kDa)

IB: p16 (16kDa)

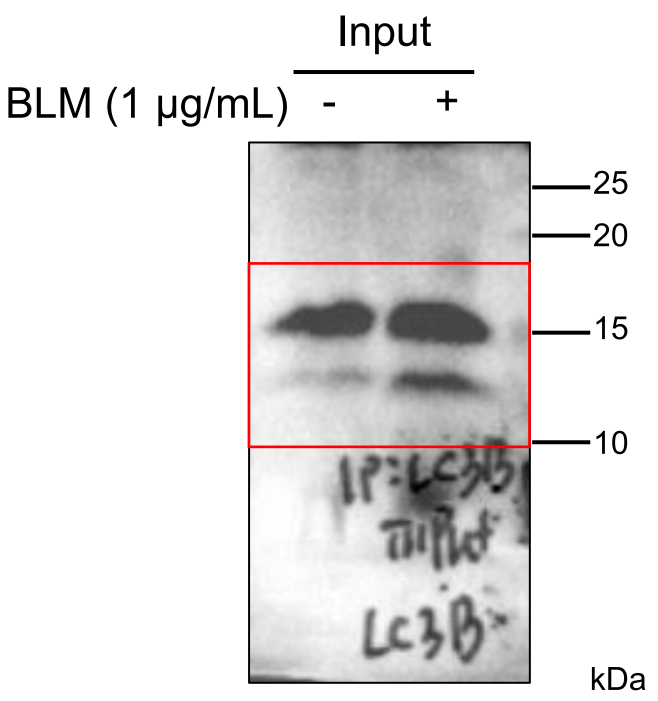

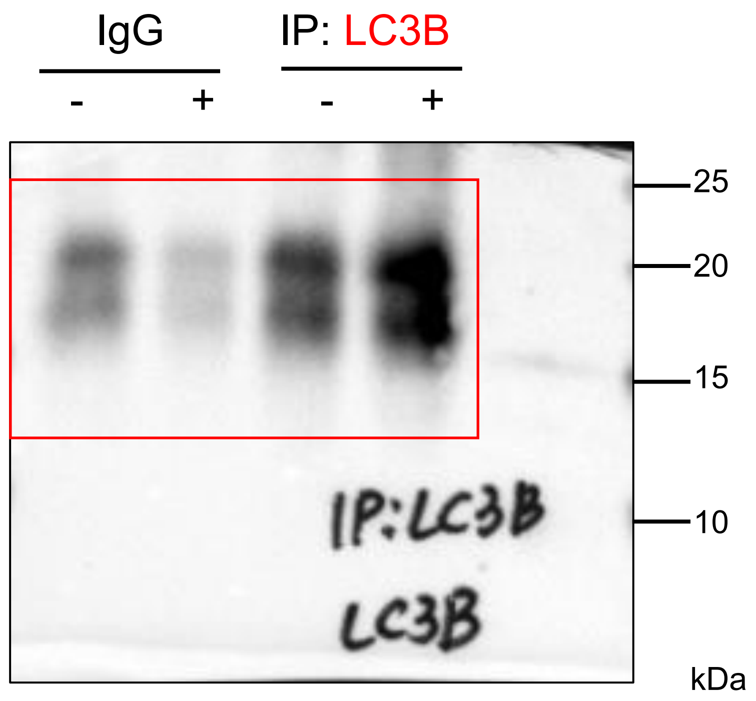

IB: LC3B (16, 14kDa)

IB: LC3B (16, 14kDa)

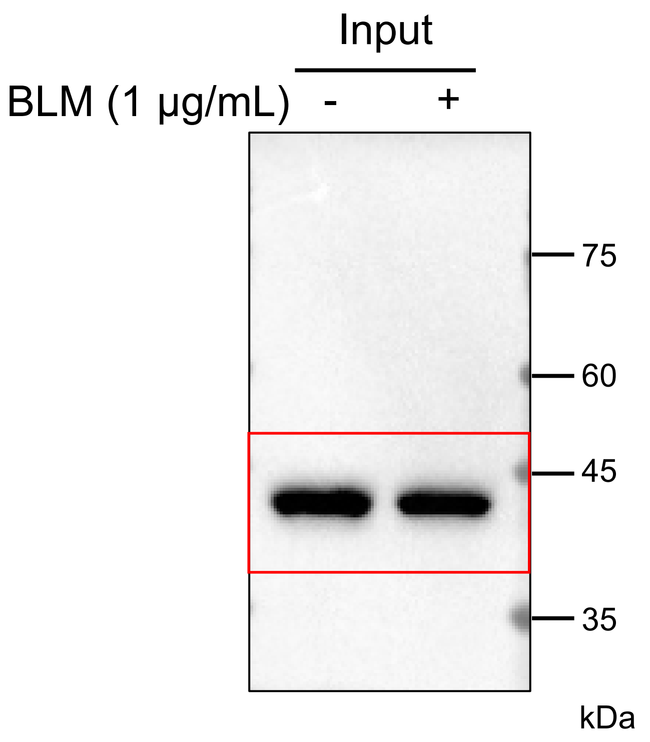

IB: β-actin (45kDa)

Supplementary Figure 8, N=1

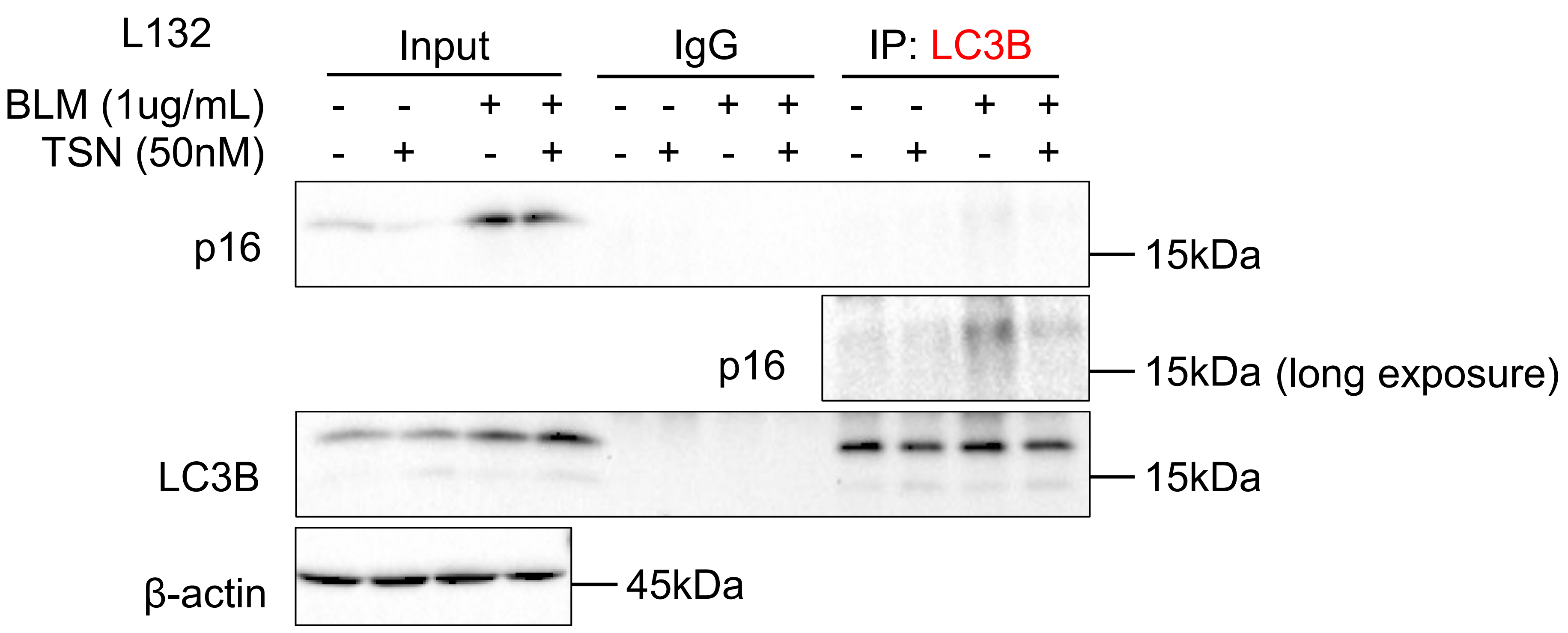

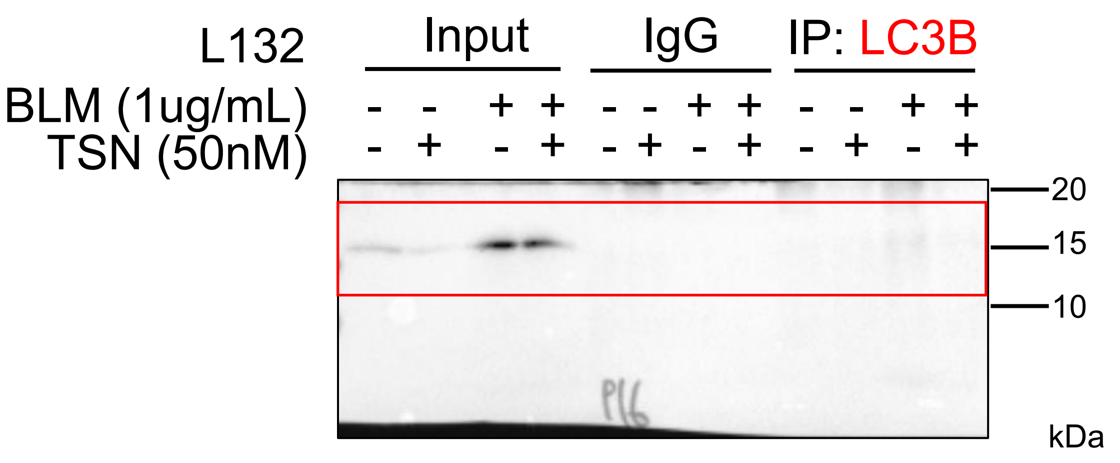

IB: **p16 (16kDa)**

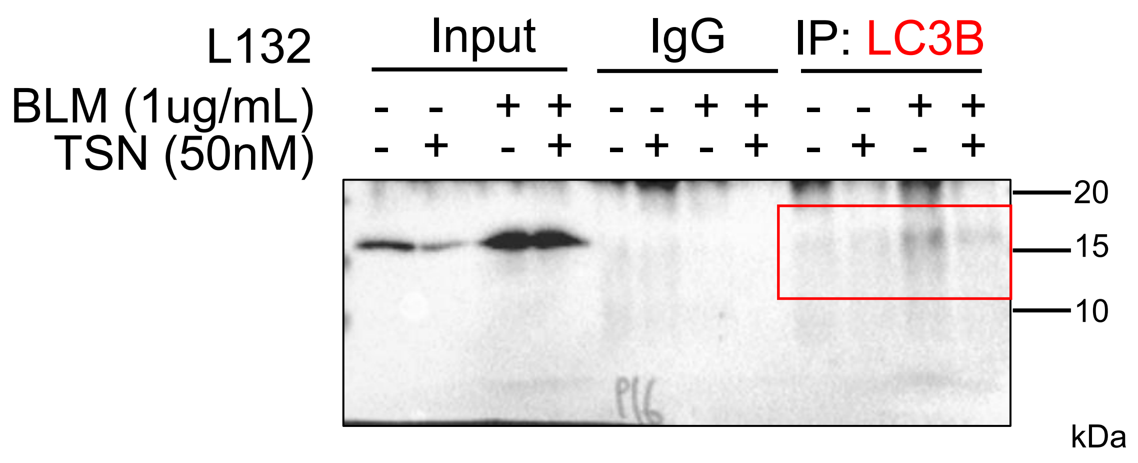

IB: p16 (16kDa) long exposure

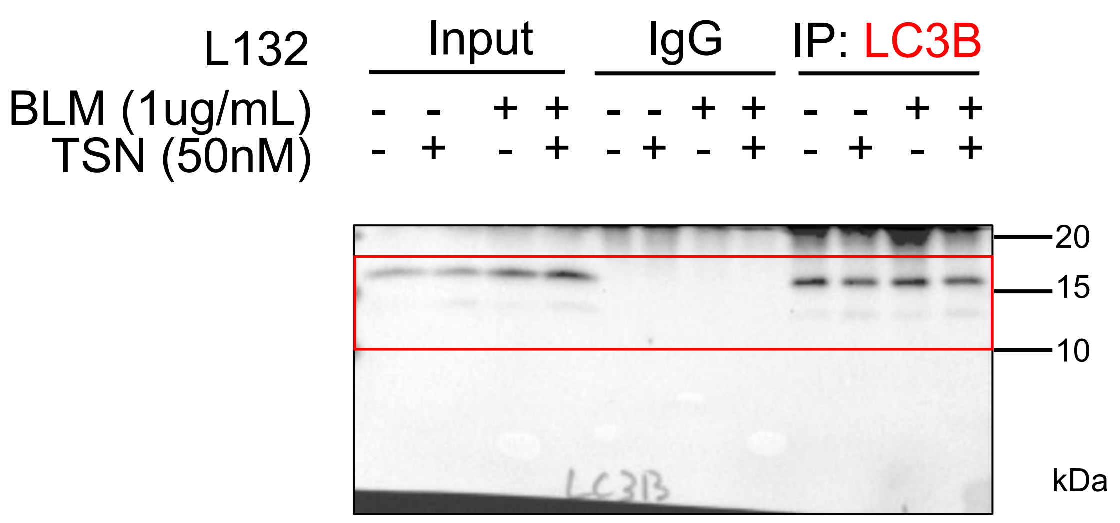

IB: LC3B (16, 14kDa)

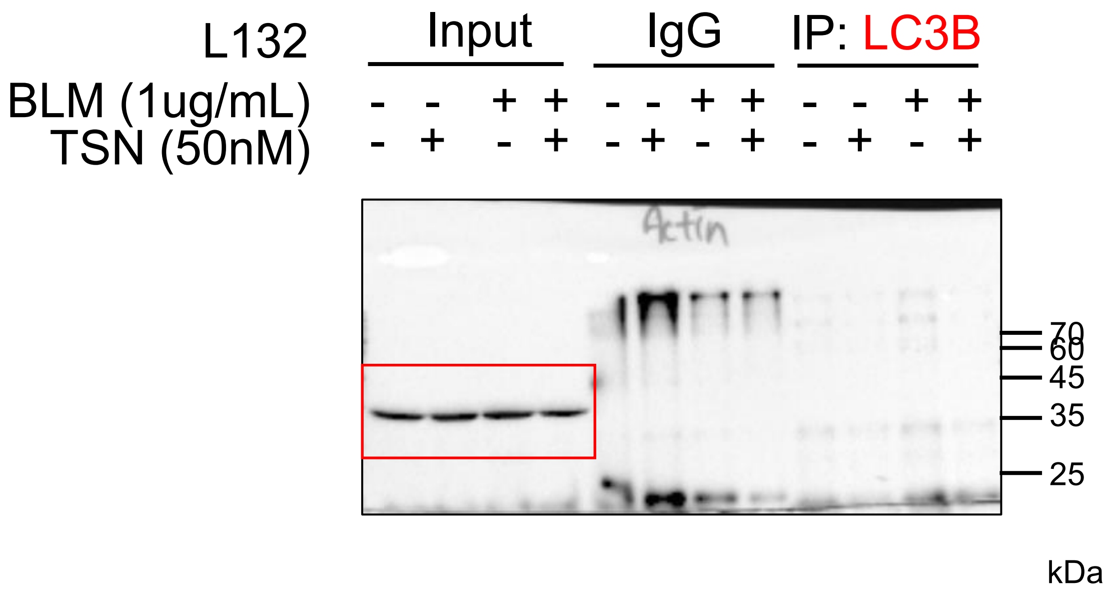

IB: β-actin (45kDa)

Supplementary Figure 8, N=2

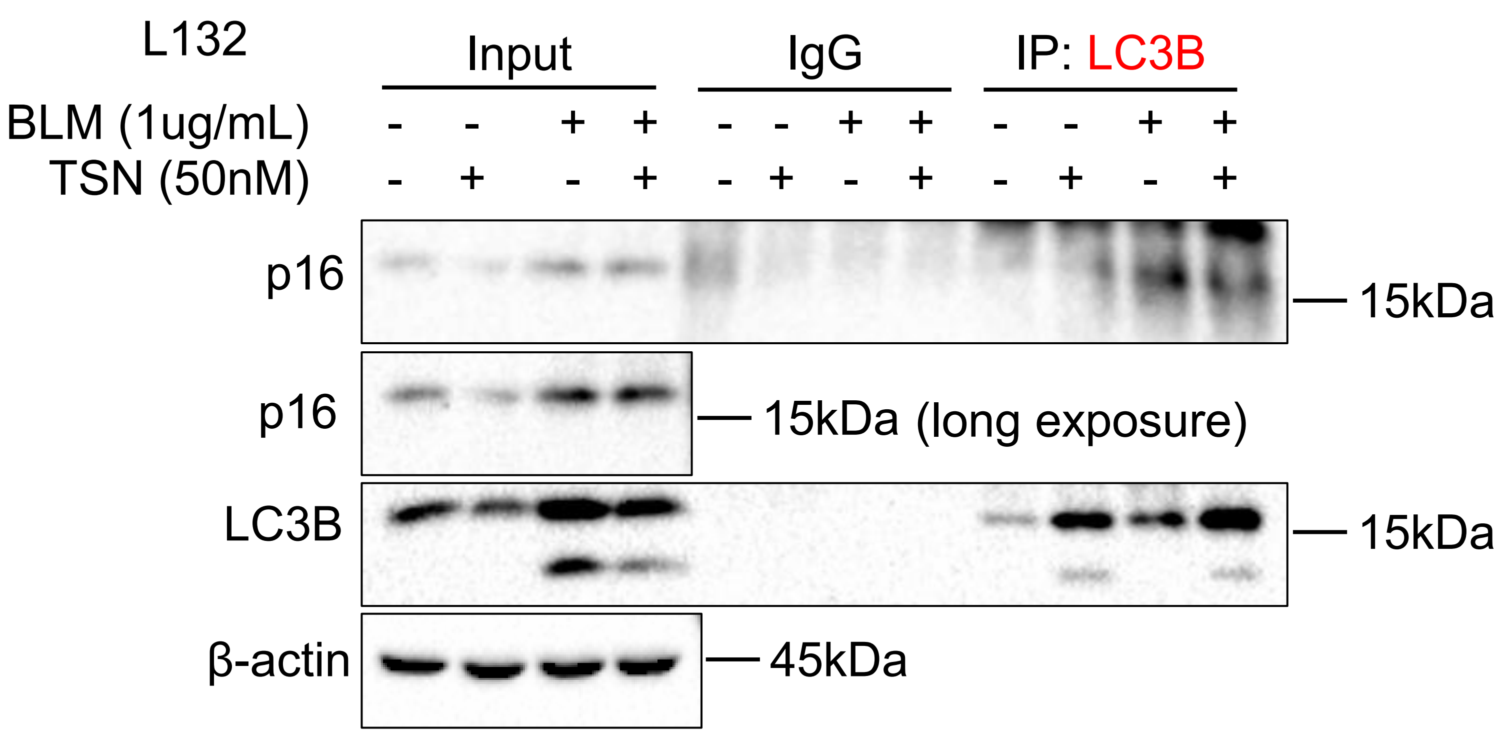

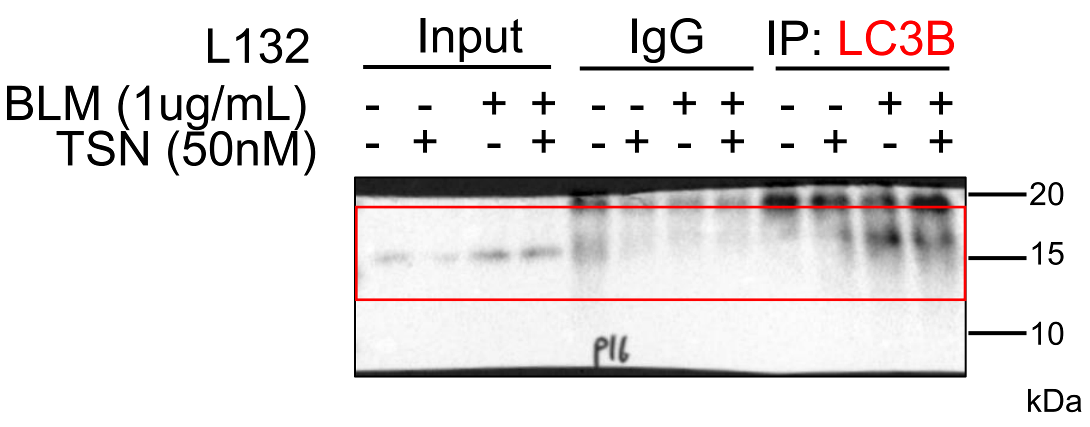

IB: p16 (16kDa)

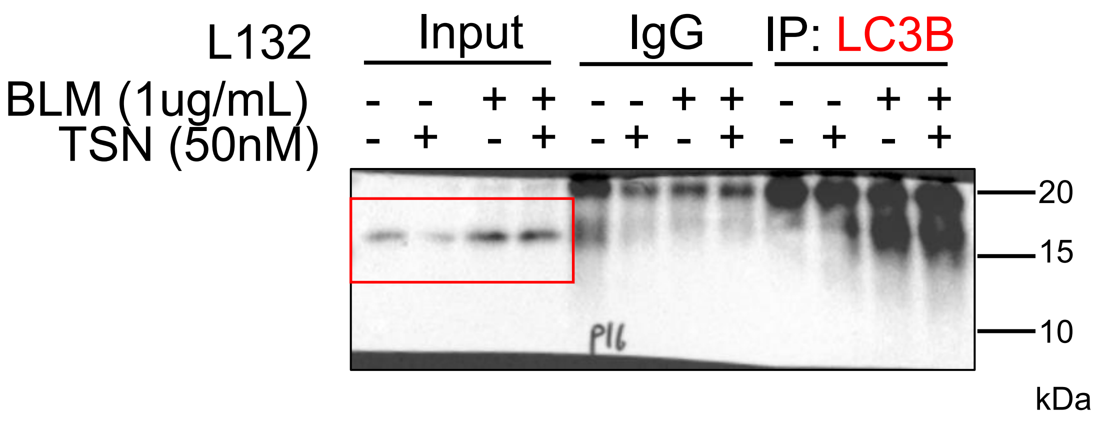

IB: p16 (16kDa) long exposure

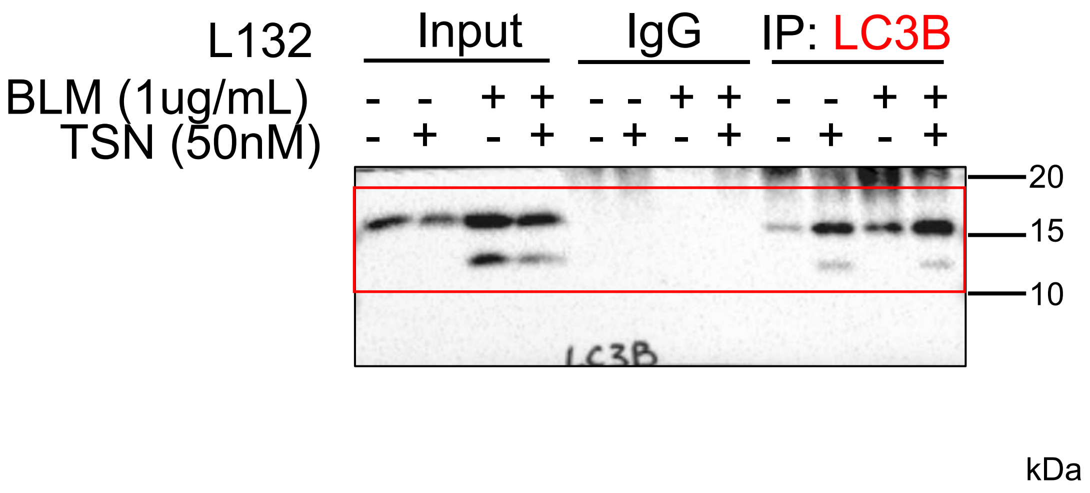

IB: LC3B (16, 14kDa)

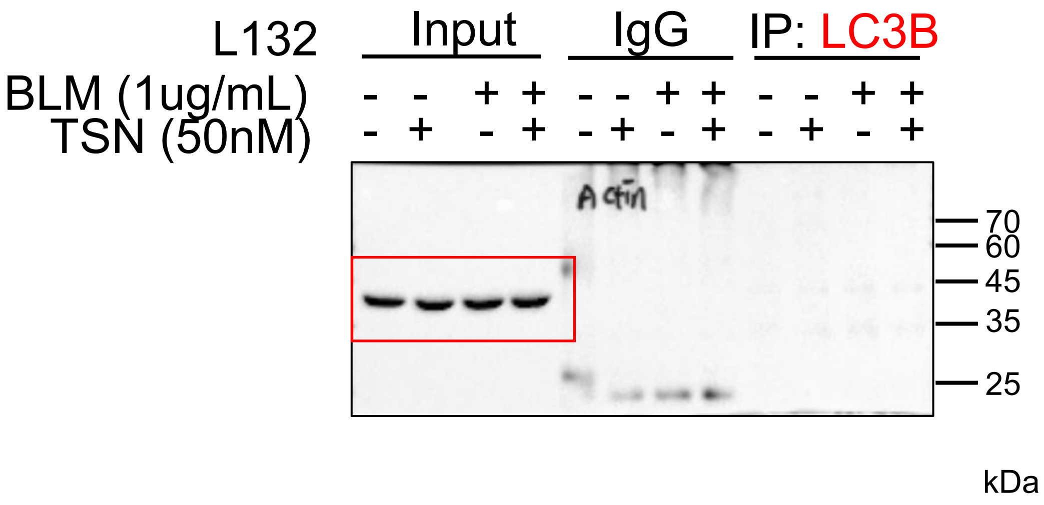

IB: β-actin (45kDa)

Supplementary Figure 8, N=3

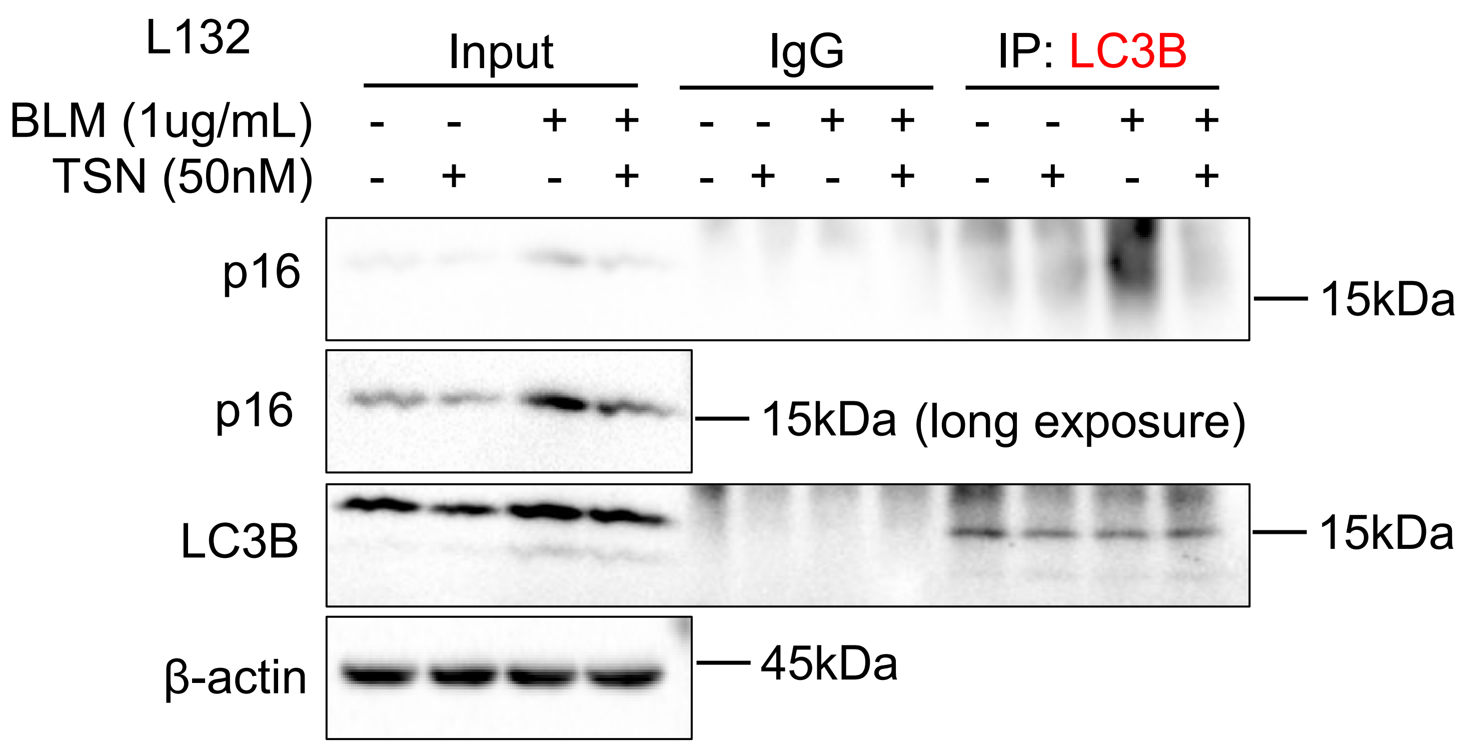

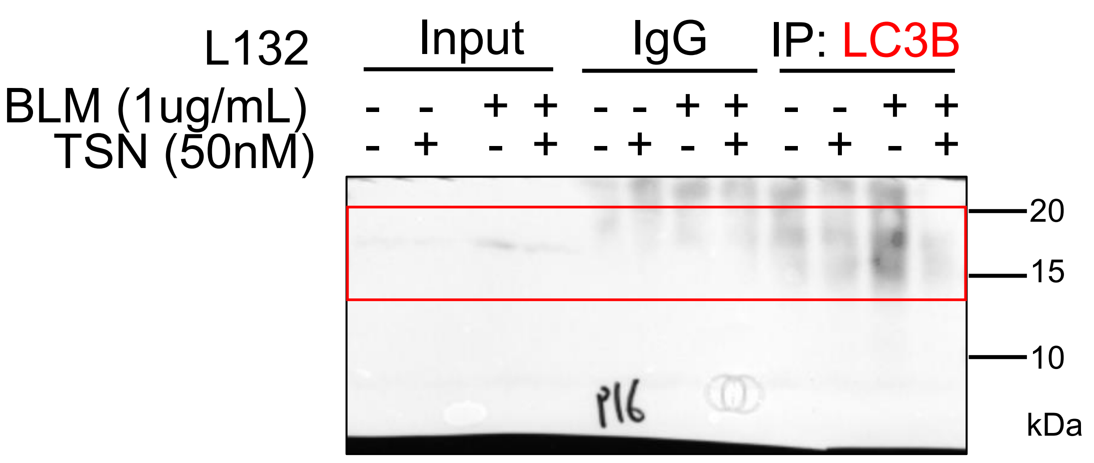

IB: p16 (16kDa)

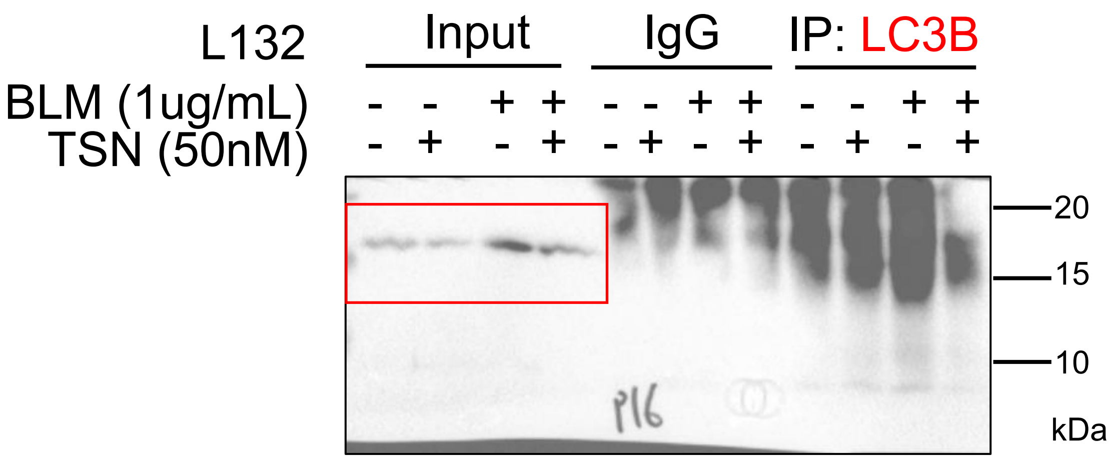

IB: p16 (16kDa) long exposure

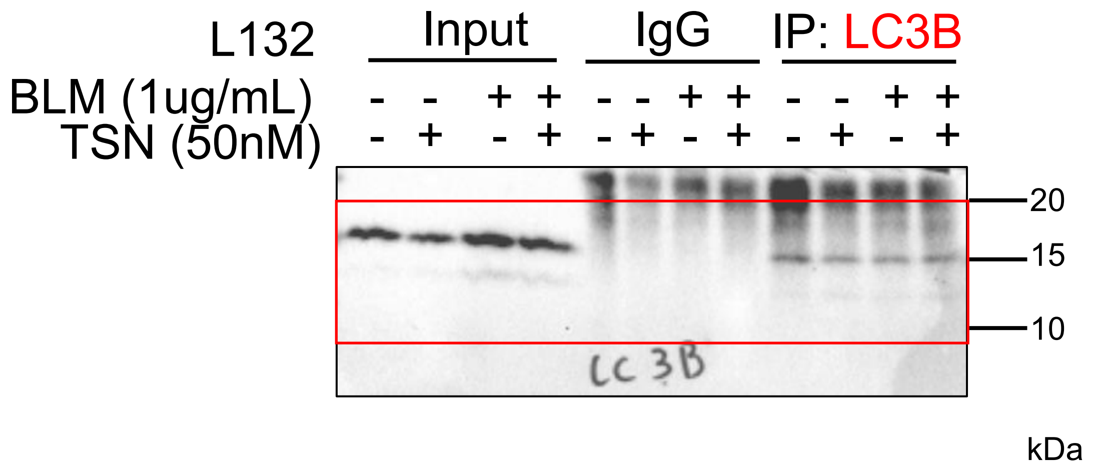

IB: LC3B (16, 14kDa)

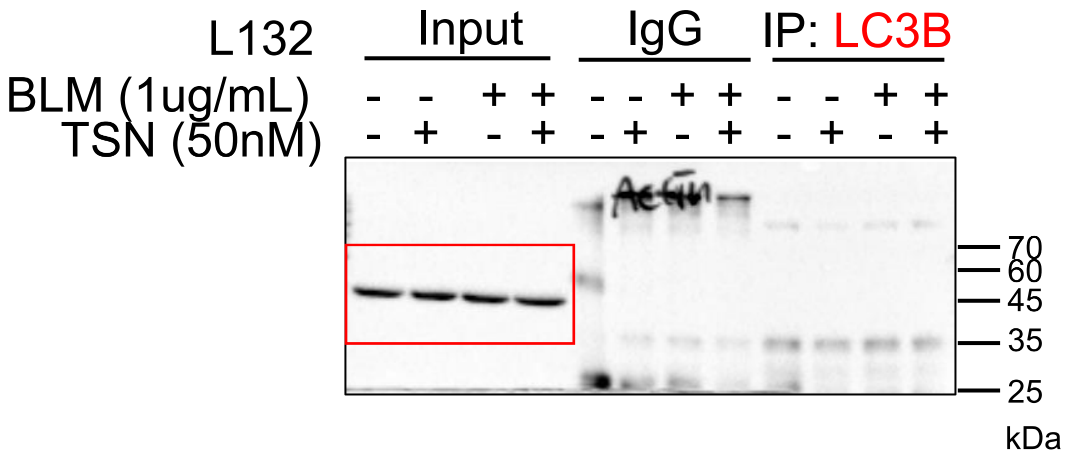

IB: β-actin (45kDa)

Supplementary Figure 8, N=4

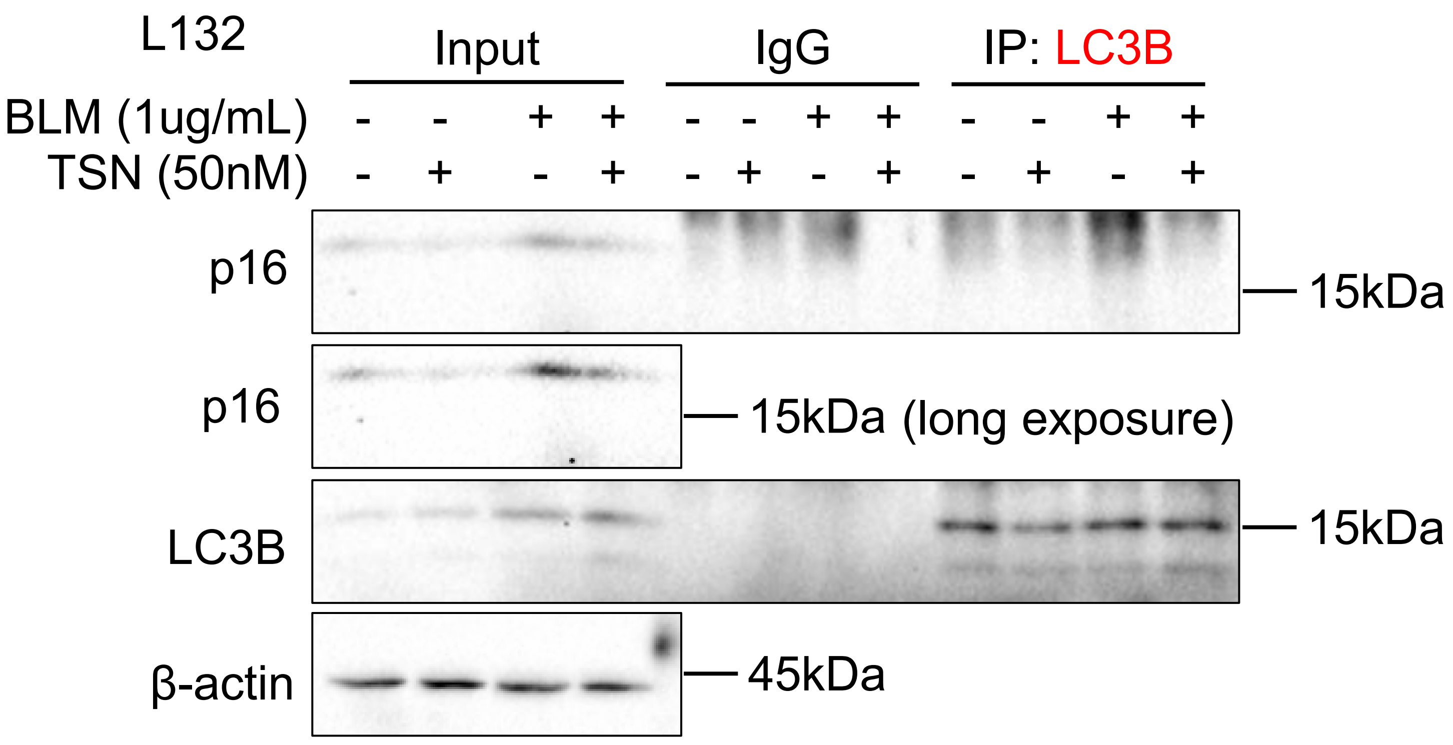

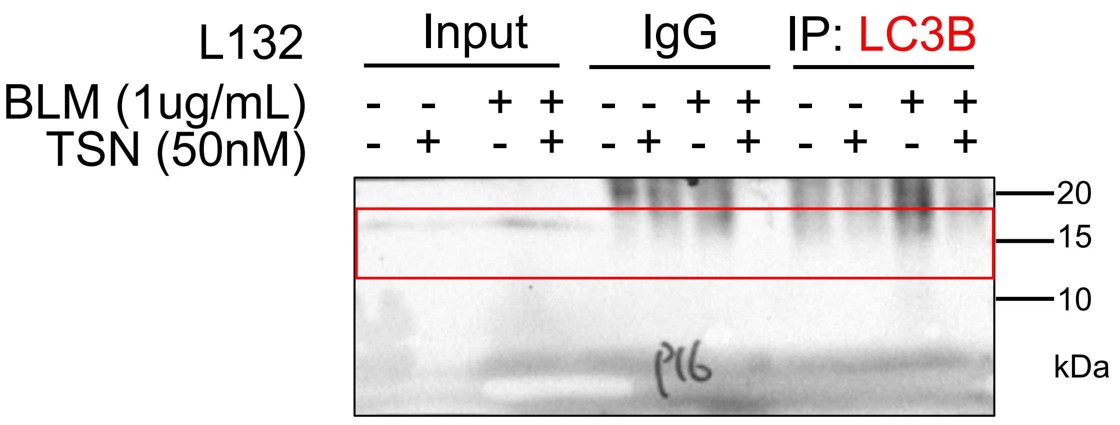

IB: p16 (16kDa)

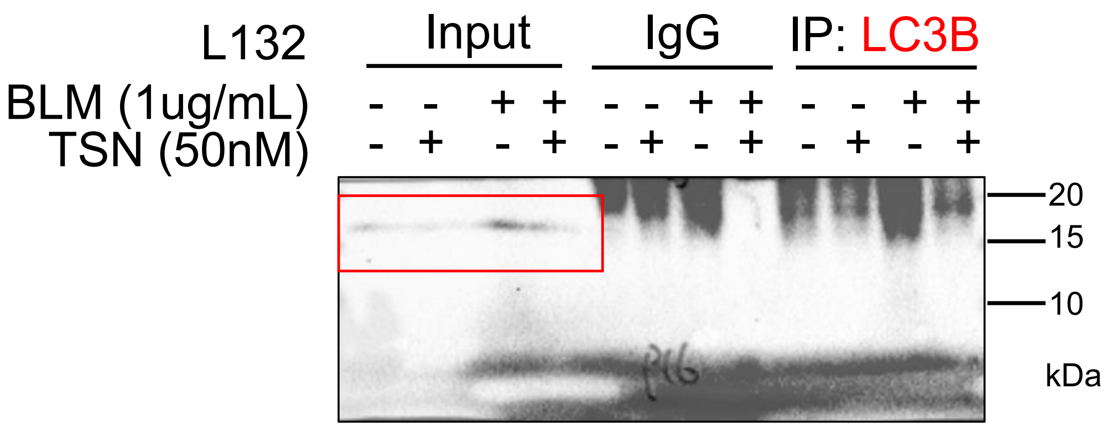

IB: p16 (16kDa) long exposure

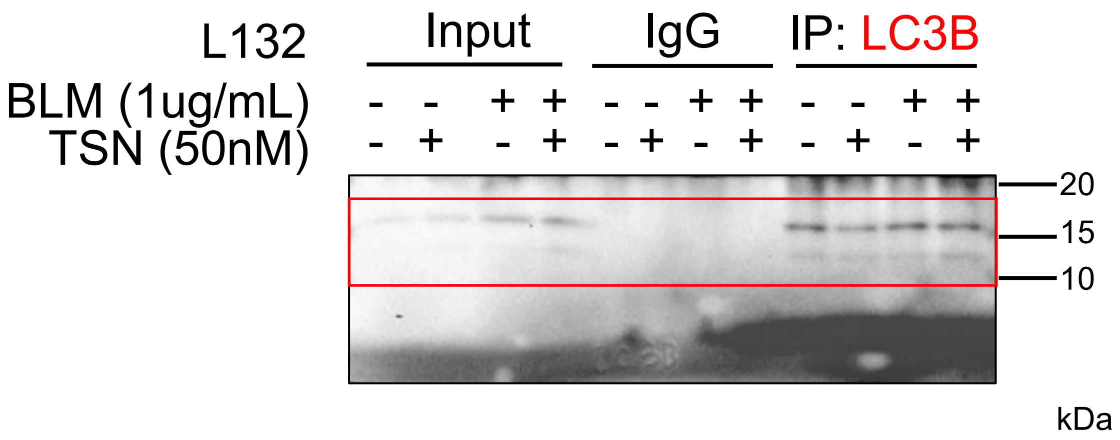

IB: LC3B (16, 14kDa)

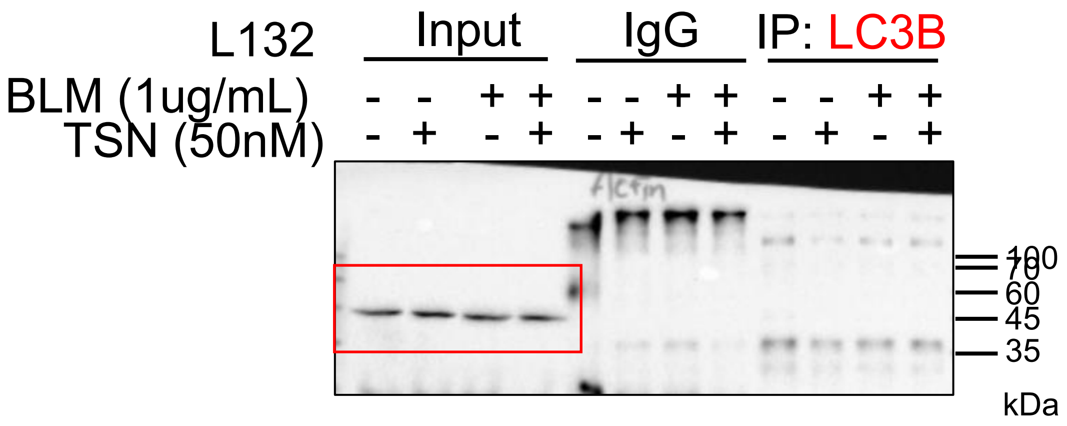

IB: β-actin (45kDa)
